# An isolable, crystalline complex of square-planar silicon(IV)

**DOI:** 10.1016/j.chempr.2021.05.002

**Published:** 2021-08-12

**Authors:** Fabian Ebner, Lutz Greb

**Affiliations:** 1 Anorganisch-Chemisches Institut, Ruprecht-Karls-Universität Heidelberg, Im Neuenheimer Feld 275, 69120 Heidelberg, Germany

**Keywords:** silicon, planar, p-block element, structural constraint, agostic interaction, bond activation, ligand-element charge transfer, element-ligand cooperativity

## Abstract

The structure and reactivity of silicon(IV), the second most abundant element in our Earth's crust, is determined by its invariant tetrahedral coordination geometry. Silicon(IV) with a square-planar configuration (*ptSi*^*IV*^) represents a transition state. Quantum theory supported the feasibility of stabilizing *ptSi*^*IV*^ by structural constraint, but its isolation has not been achieved yet. Here, we present the synthesis and full characterization of the first square-planar coordinated silicon(IV). The planarity provokes an extremely low-lying unoccupied molecular orbital that induces unusual silicon redox chemistry and CH-agostic interactions. The small separation of the frontier molecular orbitals enables visible-light ligand-element charge transfer and bond-activation reactivity. Previously, such characteristics have been reserved for d-block metals or low-valent p-block elements. Planarization transfers them, for the first time, to a p-block element in the normal valence state.

## Highlights

• The first square-planar silicon(IV) is isolated
• A low-lying LUMO provokes CH-agostic interaction and visible-light absorption
• Transition metals are mimicked without leaving the natural oxidation state

## The bigger picture

Tetrahedral silicon(IV) compounds are the building blocks of our Earth’s crust. Here, we describe the first species of silicon(IV) with a square-planar configuration. The structural deformation has substantial consequences for the compounds’ physicochemical properties and imparts features usually associated with transition metals. Upon planarization, the frontier molecular orbital gap shrinks by more than 50% and enables ligand-element charge transfer, CH-bond agostic interactions, and spontaneous reactivity with inert bonds. Small frontier molecular orbital gaps are critical for bond-activation reactivity, catalysis, and photochemistry with transition metals. Traditional approaches to mimic these characteristics with the more abundant p-block elements rely on unusual valence or oxidation states. With the realization of square-planar silicon(IV), these peculiarities start reaching p-block elements in their natural oxidation states.

## Graphical abstract

**Figure:**
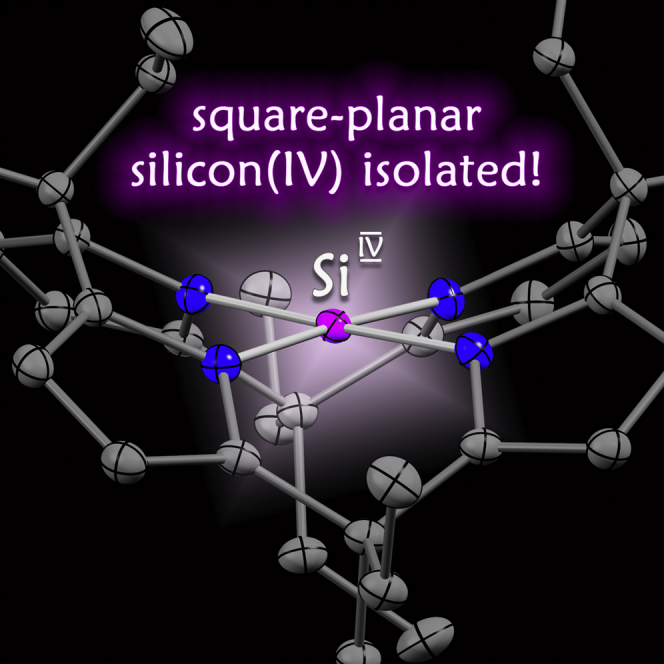

## Teaser

Our Earth’s crust is covered with compounds of silicon(IV). In each, tetracoordinated silicon(IV) arranges its four substituents in a tetrahedral fashion, while other environments are unknown. This work describes the isolation and properties of a molecular complex with square-planar silicon(IV). The flattened structural motif provokes a range of features that primes the second most abundant element for new applications in catalysis and materials science.

## Introduction

**Figure 1:**
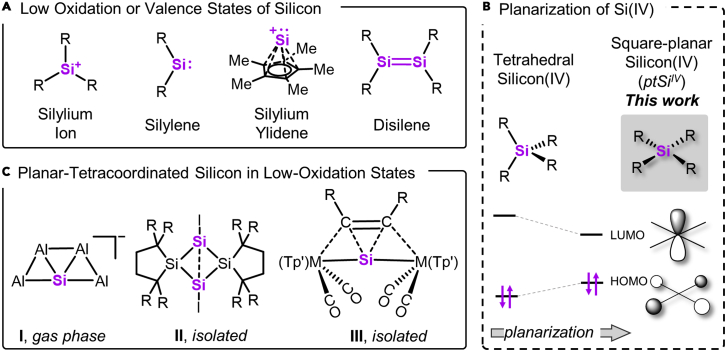
Molecular silicon complexes (A) Selection of previously isolated low-oxidation or low-valent states of silicon. (B) Reported compounds with planar-tetracoordinated silicon in low-oxidation states (M = W, Mo). (C) Planarization of normal-valent silicon(IV), the effected changes of the frontier molecular orbital energy, and the representation of HOMO and LUMO in *ptSi*^*IV*^.

Silicon is the second most abundant element in the Earth's crust, constituting 28 wt % as tetrahedral silicon(IV).^1^ During past decades, groundbreaking low-valent states of silicon, such as silylium ions,^2^^,^^3^ silylenes,^4^, ^5^, ^6^ silylium ylidenes,^7^, ^8^, ^9^, ^10^ disilenes,^11^ disilynes,^12^ trisilaallene,^13^ or silylones,^14^^,^^15^ have been isolated (Figure 1A).^16^ The modified electronic structure in those compounds compared with normal-valent silicon(IV) evolved into a linchpin for unique reactivities and catalytic applications.^16^, ^17^, ^18^, ^19^, ^20^, ^21^ Naturally, these achievements channeled the primary interest of modern silicon chemistry on low-valent states while pushing conventional silicon(IV) into a niche of being precursor with classical behavior only. However, it is not only the valence or oxidation state that changes the reactivity and properties of an element, but likewise the type and the spatial arrangement of its ligands.^22^

Structural constraint of group 13 and 15 p-block elements for reactivity enhancement is burgeoning in recent years^23^, ^24^, ^25^, ^26^, ^27^, ^28^, ^29^, ^30^ but remain less explored for group 14. For silicon *in unnatural oxidation states*, planar-tetracoordinated states (*ptSi*) have been observed in the gas phase (**I**, Figure 1B),^31^ in polysilanes (**II**),^32^^,^^33^ or in trapezoid complexes with transition metals (**III**).^34^ However, the isolation of planar-tetracoordinated silicon in the *intrinsically more stable, natural oxidation state IV* (*ptSi*^*IV*^) has not been achieved.^35^, ^36^, ^37^, ^38^ Indeed, the square-planar configuration of silicon(IV) constitutes a high-energy transition state during silicon inversion.^39^ Judged by the computed electronic structure of *ptSi*^*IV*^, fundamental changes can be expected upon planarization.^39^^,^^40^ The *ptSi*^*IV*^ has a silicon-centered *p*_*z*_-type lowest unoccupied molecular orbital (LUMO) of much lower energy than the tetrahedral state and a ligand-centered highest occupied molecular orbital (HOMO) raised in energy (Figure 1C).^39^^,^^41^ However, further computational studies supported the feasibility of stabilizing *ptSi*^*IV*^ into a ground state by appropriate substituents.^42^^,^^43^ If incorporated into extended 2D-structures, unique electronic properties were attributed to *in silico*-designed *ptSi*^*IV*^*-*materials that await experimental realization.^41^^,^^44^, ^45^, ^46^ More recent computational studies on molecular complexes of *ptSi*^*IV*^ even proposed carbenoid character to the square-planar silicon(IV).^47^ Ultimately, it is the small HOMO-LUMO gap that renders d-block metals or p-block elements in the low-valence states as suitable for bond activation.^48^ Hence, planarization might project those beneficial features to silicon—while staying in the normal oxidation state. This assumption is verified in this work using the macrocyclic calix[4]pyrrolato ligand.^49^^,^^50^

## Results and discussion

### Synthesis and characterization of the planar-tetracoordinated silicon complex

**Figure 2:**
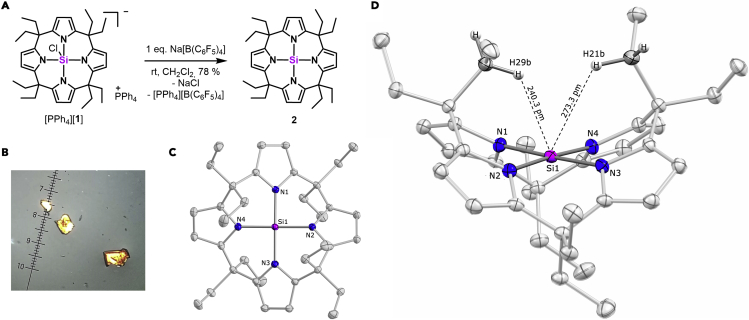
Synthesis and molecular structure of **2** (A) Synthesis of **2**. (B) Microscopic photograph of crystals of **2**. (C) Top-view of molecular structure of **2** as obtained by SCXRD/Hirshfeld atom refinement. (D) Side-view of the molecular structure of **2**, showing hydrogen-silicon distances. Hydrogen atoms are refined freely and anisotropically, depicted at fixed positions, other atoms as thermal displacement ellipsoids of 50% probability. Selected bond distances (pm) and angles (°): N1–Si 178.84(5), N2–Si 179.26(5), N3–Si 179.90(4), N4–Si 178.81(4), H21b–Si 273.3(8), H29b–Si 240.3(8), H27b–Si 258.5(8), H35b–Si 296.3(8), N1–Si–N3 178.19(2), N2–Si–N4 176.08(2), N1–Si–N2 90.15(2), N2–Si–N3 89.28(2), N3–Si–N4 90.43(2), N4–Si–N1 90.26(2).

The reaction of the tetra lithium salt of *meso*-octaethyl-calix[4]pyrrole with SiCl_4_ in dimethoxyethane and subsequent salt metathesis with PPh_4_Cl provided the *meso*-octaethyl-calix[4]pyrrolato chlorido silicate [PPh_4_][**1**] in an overall isolated yield of 36% up to 600 mg (Figure 2A). A ^29^Si-NMR chemical shift of —129 ppm in CD_2_Cl_2_ and single-crystal X-ray diffraction analysis (SCXRD, Figure S24) verified the structure of the first anionic SiN_4_Cl motif, showing structural parameters similar to the hydridosilicate reported previously.^40^ The reaction of [PPh_4_][**1**] with sodium tetrakis(pentafluorophenyl)borate in CH_2_Cl_2_ afforded a macrocyclic, tetrameric sodium salt of the chlorido silicate [Na]_4_[**1**]_4_ by salt metathesis (see Figure S25 for SCXRD). Stirring a solution of [Na]_4_[**1**]_4_ in *n*-hexane induced the transformation into a new, clean reaction product **2**, isolated in 78% yield. The ^29^Si-NMR chemical shift of **2** (−55.6 ppm) indicates a tetracoordinated silicon center. The occurrence of only one peak for the pyrrole ring protons in the ^1^H NMR spectrum, and a 2-fold set of signals for the ethyl group, illustrates a highly symmetric species in line with effective *D*_*2d*_ symmetry on the NMR time scale. Upon storing a solution of **2** in CH_2_Cl_2_ for 7 days at −40°C, block-shaped orange crystals developed (Figure 2B). SCXRD analysis revealed a tetracoordinated silicon with typical Si–N bond lengths (178.7(1) to 179.7(1) pm) and an almost ideal square-planar coordination geometry (N1–Si–N3 = 178.2(1)°, N2–Si–N4 = 176.1(1)°, N1–Si–N2 = 90.2°, Figures 2C and 2D).

The location of all protons in the difference Fourier map and structure solution by Hirshfeld atom refinement^51^ (R_1_ = 2.03; wR_2_ = 4.36) disclosed another structural peculiarity: two of the four inward-oriented methylene groups tilt toward the silicon center, reflected by varying CH^….^Si distances, e.g., Si–H21b (273.3(8) pm) versus Si–H29b (240.3(8) pm) (Figure 2D). In the solid-state crystal packing, neither intermolecular contacts can be made responsible for this distortion nor does it occur in the isoelectronic aluminate.^52^ Hence, an attractive intramolecular CH^….^Si interaction was suspected as the cause. ^1^H coupled ^13^C-NMR spectroscopy shows a coupling constant for the inward-oriented methylene groups (^1^J_C–H_ = 123 Hz, Figure S13) that is effectively reduced by 32 Hz (averaged over the 8 protons, absolute reduction 4 Hz) compared with the outward CH_2_-groups or the protonated, free ligand (^1^J_C–H_ = 127 Hz, Figure S14). A further spectroscopic indication for an interaction is provided by FTIR-vibrational spectroscopy, revealing a shoulder in the C–H vibrational band for **2**, red-shifted by 57 cm^−1^, which is absent in the protonated, free ligand or the chlorido silicate **1**^**−**^ (Figure S32). Such observations, although weak in this case, are characteristic for C–H agostic interactions in transition metal complexes^53^^,^^54^ but unusual as the source of structural deformation in p-block element chemistry. E–H σ-bond donor-acceptor interactions in the p-block were only observed for combinations of more hydridic bonds with the most Lewis acidic boranes,^55^^,^^56^ silylium ions,^2^^,^^57^ anagostic,^9^ or structurally enforced.^58^

**Figure 3:**
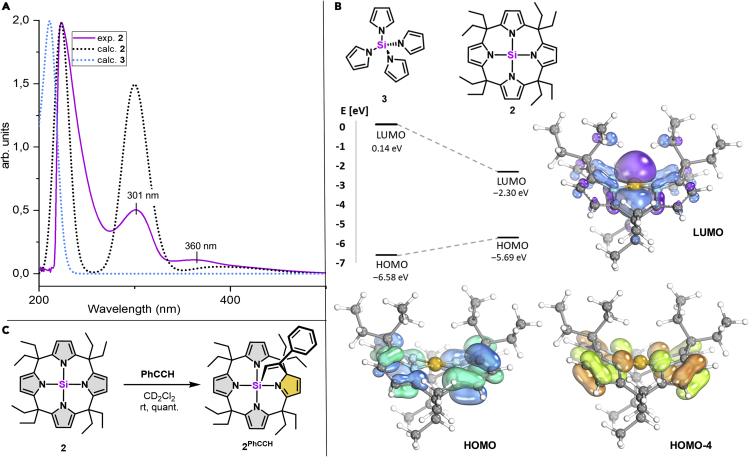
Experimental and computed properties of **2** (A) Experimental UV-vis spectrum of **2** (purple curve) in CH_2_Cl_2_ (69.5 μM), calculated (CAM-B3LYP/def2-TZVPP, shifted by +22 nm) curves of **2** (black curve), and **3** (blue curve). (B) Energies and isodensity plots of the frontier molecular Kohn-Sham orbitals of **2** and **3**. (C) Reactivity of **2** observed with phenylacetylene.

The UV-vis spectrum of **2** in CH_2_Cl_2_ (69.5 μM) showed strong absorption bands at 301 and 360 nm, with a pronounced shoulder reaching into the visible range (Figure 3A). This observation contrasts with the non-chromophoric nature of every other tetracoordinated silane, e.g., the literature known tetra(N-pyrrolyl)silane **3** (Figure 3B),^59^ and even with that of tris(N-pyrrolyl)borane,^60^ but indicates an unusually small HOMO-LUMO gap.

### Redox chemistry and bond activation with 2

Cyclic voltammetry revealed an irreversible reduction peak for **2** at a potential of ‒1.83 V versus Fc/Fc^+^, which was assigned to the formation of the silicon-centered radical anion **2**^**⋅−**^ (Figure S36). This reduction potential is exceptionally mild for silicon(IV), but in the range of electron-deficient boranes such as B(C_6_F_5_)_3_.^61^^,^^62^ Indeed, tetracoordinated silicon radical anions remain elusive as they are presumed to be powerful electron donors, even stronger than alkali metals.^63^ A chemical reduction of **2** was attempted with decamethylcobaltocene (−1.9 V versus Fc/Fc^+^) as a reducing agent to support the electroanalytical results. Performed in C_6_D_6_, a rapid reaction to a mixture of unidentified products indicated a redox process to happen. Performed in CD_2_Cl_2_ as the solvent, conversion to the chlorido silicate [CoCp^∗^_2_][**1**] was observed (Figure S16). This finding supports the formation of a silicon-centered radical anion that abstracts a chlorine atom from the solvent (see Figure S39 for the computed spin density of **2**^**⋅−**^). Hence, planarization enables silicon-based radical chemistry under mildly reducing conditions.

To probe the potentially small HOMO-LUMO gap of the planar silane in bond activation, a solution of **2** in CD_2_Cl_2_ was subjected to phenylacetylene at room temperature. A rapid and quantitative silicon-ligand cooperative 1,2-addition of **2** to the carbon-carbon triple bond was verified by the occurrence of characteristic ^1^H- and ^29^Si-NMR spectroscopic patterns and mass spectrometry (Figures 3C and S19–S23). This interpretation was corroborated computationally for the model octamethyl-calix[4]pyrrolato silane by favorable thermodynamics (ΔG = −117 kJ mol^−1^) and a low reaction barrier (ΔG^‡^ = 61 kJ mol^−1^) (see Figure S46). It represents an unprecedented, spontaneous carbosilylation of an alkyne with high synthetic potential, and the first example of silicon-element ligand cooperativity in its classical sense.^64^

### Quantum chemical analysis of the electronic structure and spectroscopic features of 2

All experimental and spectroscopic results point to a unique electronic structure of **2**, which was thus analyzed by computational methods. Structure optimization of **2** validates the square-planar silicon (N–Si–N = 177°) as the energetic minimum. The LUMO in **2** has a strongly localized p_z_-character at silicon with an energy lower by 2.4 eV than the tetrahedral **3** (Figure 3B). This low-lying LUMO explains the experimentally observed uptake of an electron in the redox studies. Indeed, the computed electron affinity for **2** (165 kJ mol^−1^) is substantially higher than for tetrahedral **3** (6 kJ mol^−1^). The HOMO of **2** is located at the ligand's π-system, and its energy is higher in comparison with **3** by 0.9 V. The reduced HOMO-LUMO gap rationalizes the light absorption in the visible range, and the time-dependent density functional theory (TDDFT) computed UV-vis spectrum is in line with the experimental one (Figure 3A). Inspection of the natural transition orbitals assigns the band at 360 nm to a *π*(C=C_pyrrole_) → Si(p_z_) charge transfer (HOMO/LUMO) and the more intense band at 301 nm as N(LP) → Si(p_z_) charge transfer (HOMO-4/LUMO) (Figures 3B and S45). Those transitions are absent in the UV-vis spectrum of tetrahedral **3**, which shows ligand-based *π*→*π∗* excitation as the lowest energy transition (190 nm). Such a ligand-element charge transfer is very unusual for light p-block elements and underscores the π-donor stabilization in the square-planar silicon state.^39^^,^^42^ This effect is also corroborated by natural bond orbital (NBO) analysis, which finds substantial *π*-delocalization of the nitrogen atom lone pair into the Si(p_z_) acceptor orbital, amounting to roughly 10% of the total Si–N bond interaction energy (Table S6).

Last, the agostic or σ-donor type interaction of the endo-methylene C–H bonds with the square-planar silicon in **2** was considered computationally. Although the experimentally observed methylene group tilting was not found in the DFT-computed global minimum structure, the red-shifted IR bands for the endo-methylene C–H bonds were reproduced in the computed IR frequencies (Figure S38). Moreover, quantum theory of atoms in molecules (QTAIM) on the experimental SCXRD-HAR structure's electron density revealed a bond critical point between the inwards tilted H29b and the silicon center (*ρ*(**r**) = 0.02 a.u., Figure S42), in line with weak but traceable interactions also found for d^0^-metals.^54^ Further, NBO analysis disclosed significant σ_CH_ → Si(p_z_) second-order perturbation energies (38 kJ mol^−1^) and systematically depleted NBOs of the inward σ_CH_ bond in comparison with the other methylene CH-bond (Figure S40; Table S5). Ultimately, DLPNO-CCSD(T)/def2-QZVPP computations yielded an interaction enthalpy of −14 kJ mol^−1^ between ethane and the hypothetical octaprotio-calix[4]pyrrolato silane **4** (Figure S43; Table S7). The very low lying, silicon-centered LUMO in **2** provides a strong driving force for this orbital-controlled interaction, potentially supported by London dispersive attraction.^65^

### Conclusion

The first square-planar silicon(IV) species **2** is isolated and characterized. The compound undergoes unprecedented silicon redox chemistry, light-induced ligand-element charge transfer, and bond activation in element-ligand cooperative fashion. Hence, the structural-, spectroscopic-, and reactivity features of **2** confirm a LUMO substantially lowered in energy and a HOMO-LUMO gap reduced by 50%, provoked by forcing a Si(IV)-tetrahedron into a planar structure. Small HOMO-LUMO gaps are the basis for the peculiar reactivity of d-block metals, low-valent p-block compounds, or frustrated Lewis pairs.^48^ This work establishes a fourth pillar to reach this goal. It endows the second most abundant element with new potential for catalysis, photochemistry, and materials science, while staying in the oxidation state that seemed exhaustively understood.

## Experimental procedures

### Resource availability

#### Lead contact

Further information and requests for resources and reagents should be directed to and will be fulfilled by the lead contact, Lutz Greb (greb@uni-heidelberg.de).

#### Materials availability

All unique/stable reagents generated in this study are available from the lead contact with a completed materials transfer agreement.

#### Data and code availability

Crystallographic data have been deposited in the Cambridge Crystallographic Data Center (CCDC) under accession numbers CCDC: 2042938, 2042939, 2042940, and 2042941. These data can be obtained free of charge from the CCDC at http://www.ccdc.cam.ac.uk/data_request/cif.

### Synthesis and analytical data of 2

Rigerously donor free sodium tetrakis(pentafluorophenyl)borate (68.4 mg, 28.5 μmol, 1.10 equiv) was added to a solution of [PPh_4_][**1**] (66.0 mg, 70.2 μmol, 1.00 equiv) in 4 ml CH_2_Cl_2_ at ambient temperature, and stirred for further 12 h, during which it turned yellow. The solvent was removed under reduced pressure, the residue suspended in 10 ml *n*-hexane, stirred for 2 h, and filtered. The solvent of the filtrate was removed, and the residue was dissolved in 10 ml *n*-hexane, stirred for 24 h and, filtered. The clear yellow solution was stirred for another 24 h to drive the elimination of NaCl to completeness. The yellow solution was filtered again, and the solvent was removed. After drying *in vacuo,* the neutral silane **2** was obtained as a yellow solid 78% yield (30.9 mg, 54.7 μmol). Orange crystals of **2** were obtained by storing a concentrated solution of **2** in CH_2_Cl_2_ for 7 days at -40°C. Slow decomposition in CD_2_Cl_2_ and C_6_D_6_ was observed over 4 days at room temperature. Remark: the first step in this reaction is the cation exchange from PPh_4_^+^ to Na^+^. The SCXRD of this intermediate [Na]_4_[**1**]_4_ was obtained by storing a concentrated solution of [PPh_4_][**1**] and NaBArF_20_ in DCM for 5 days at room temperature without further workup (CCDC: 2042940). ^**1**^**H NMR** (CD_2_Cl_2_, 400 MHz, 295 K) δ[ppm]: 5.89 (s, 8H), 2.04 (q, ^3^*J*_HH_ = 7.4 Hz, 8H, *CH*_*2 Ethyl*_), 1.93 (q, ^3^*J*_HH_ = 7.4 Hz, 8H, *CH*_*2 Ethyl*_), 1.09 (t, ^3^*J*_HH_ = 7.4 Hz, 12H, *CH*_*3 Ethyl*_), 0.66 (t, ^3^*J*_HH_ = 7.4 Hz, 12H, *CH*_*3 Ethyl*_). ^**13**^**C{**^**1**^**H}NMR** (CD_2_Cl_2_, 100 MHz, 295 K)δ[ppm]: 143.9 (*C1*), 106.5 (*C2*), 45.9 (*CH*_*2 Ethyl*_), 42.4 (*C3*), 24.8 (*CH*_*2 Ethyl*_), 9.4 (*CH*_*3 Ethyl*_*)*, 9.3 (*CH*_*3 Ethyl*_*)*. ^**29**^**Si HMBC NMR** (CD_2_Cl_2_, 400 MHz, 295 K): −55.6 ppm.

### Mass spectrometry

[ESI]: C_36_H_48_N_4_Si∗OH^−^: calc. 581.3675 exp. 581.3761. [MALDI, DFTB-Matrix]: C_36_H_48_N_4_Si∗H_3_O^+^: calc. 583.383 exp. 583.397.

### IR spectroscopy ṽ[cm^−1^]

3,112 (w), 2,963 (s), 2,931 (s), 2,872 (s), 2,815 (w), 1,344 (w), 1,514 (m), 1,455 (s), 1,378 (m), 1,364 (m), 1,322 (w), 1,297 (w), 1,278 (w), 1,234 (s), 1,132 (s), 1,087 (s), 973 (s), 953 (m), 926 (w), 857 (w), 736 (s), 713 (m), 681 (w).

### Crystal data for [2]

C_36_H_48_N_4_Si, M = 564.87, monoclinic, P2_1_/n (no. 14), a = 10.3052(4), b = 19.3263(7), c = 14.9876(7) Å, α = 90, β = 94.396(2), γ = 90, V = 2,976.2(2) Å^3^, Z = 4, D_c_ = 1.261 g cm^–3^, μ(Mo-Kα) = 0.112 mm^–1^, 2θ_max_ = 61°, T = 100 K, orange blocks, Bruker APEX-II CCD; 9,086 independent measured reflections (R_int_ = 0.0505), R_1_(obs) = 0.0380, wR_2_(all) = 0.1046, 406 parameters. NoSphereA2-solution: R_1_(obs) = 0.0203, wR_2_(all) = 0.0436, 802 parameters. CCDC: 2042941(ShelXl solution)/2042939 (HAR/NoSpherA2-solution).

## Supplemental information

Document S1. Supplemental experimental procedures, Figures S1–S46, Tables S1–S7, and supplemental references

Document S2. Article plus supplemental information
