# Supplementary material for: An isolable, crystalline complex of square-planar silicon(IV)
Source: Chem. 2021 Aug 12;7(8):2151–9. doi: 10.1016/j.chempr.2021.05.002 (PMC8367297; doi:10.1016/j.chempr.2021.05.002)
Supplement: Document S1. Supplemental experimental procedures, Figures S1–S46, Tables S1–S7, and supplemental references [file mmc1.pdf]

Chem, Volume *7*

Supplemental information

An isolable, crystalline complex of square-planar silicon(IV)

Fabian Ebner and Lutz Greb

Supplemental Analytic Data

NMR-Spectra

(*residual solvent peaks)

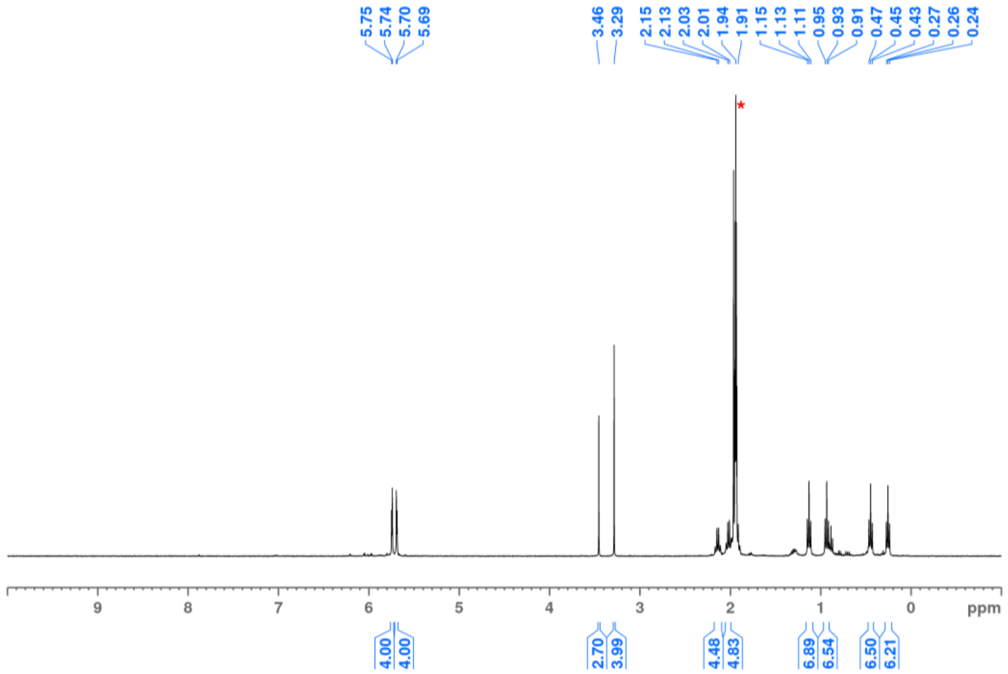

Figure S 1: ^{1}H NMR (ACN-d_3, 600 MHz, 295 K) spectrum of [Li(dme)][**1**].

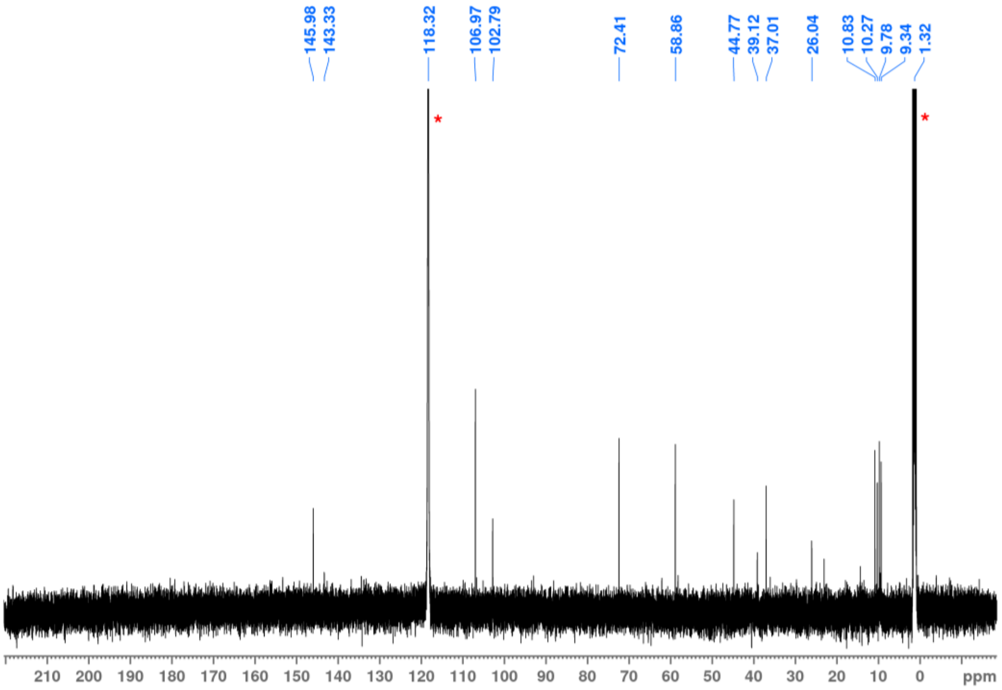

Figure S 2: ^{13}C NMR (ACN-d_3, 150 MHz, 295 K) spectrum of [Li(dme)][**1**].

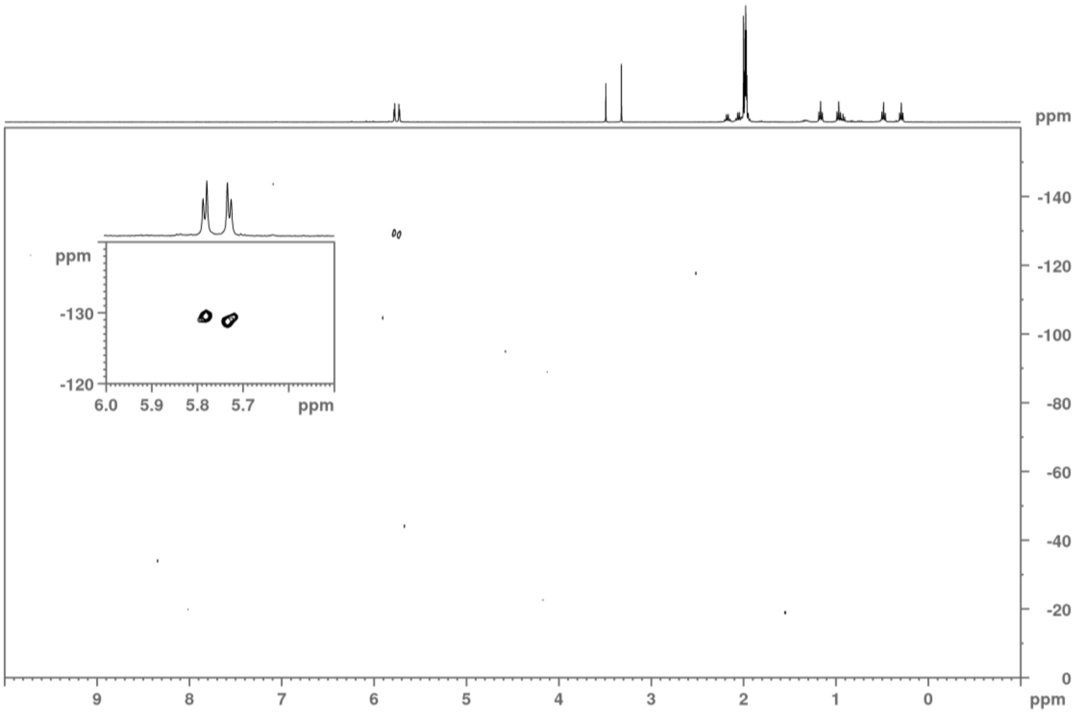

Figure S 3: ^{29}Si HMBC NMR (ACN-d_3, 600 MHz, 295 K) spectrum of [Li(dme)][**1**].

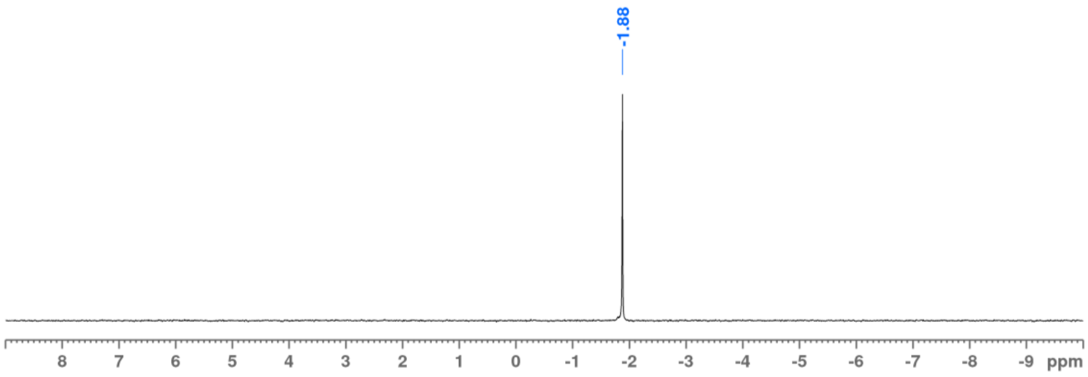

Figure S 4: ^{7}Li NMR (ACN-d_3, 155 MHz, 295 K) spectrum of [Li(dme)][**1**].

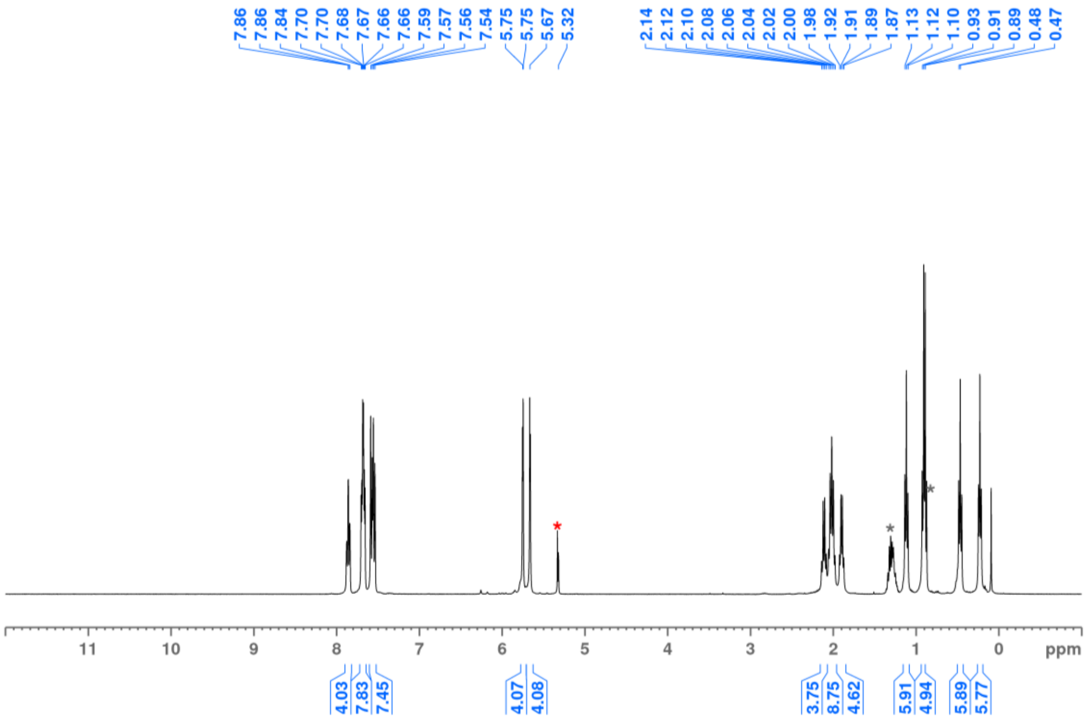

Figure S 5: ^{1}H NMR (DCM-d_2, 400 MHz, 295 K) spectrum of [PPh_4][**1**]. Gray asterisks mark residues of *n*-pentane.

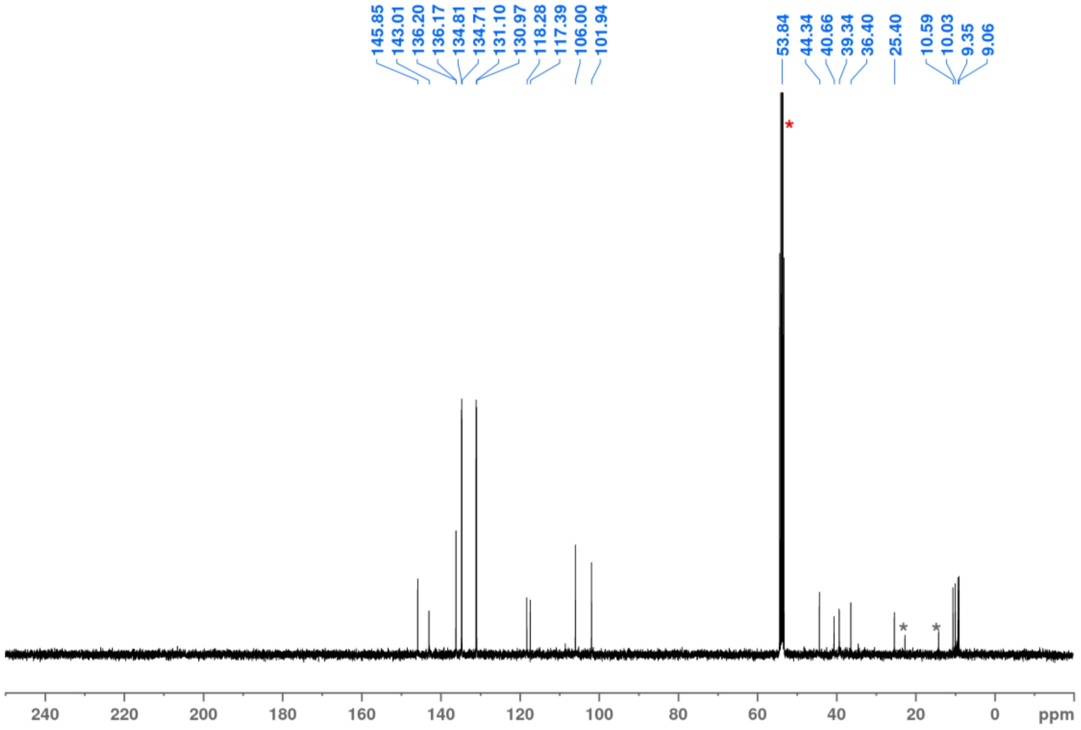

Figure S 6: ^{13}C NMR (DCM-d_2, 100 MHz, 295 K) spectrum of [PPh_4][**1**]. Gray asterisks mark residues of *n*-pentane.

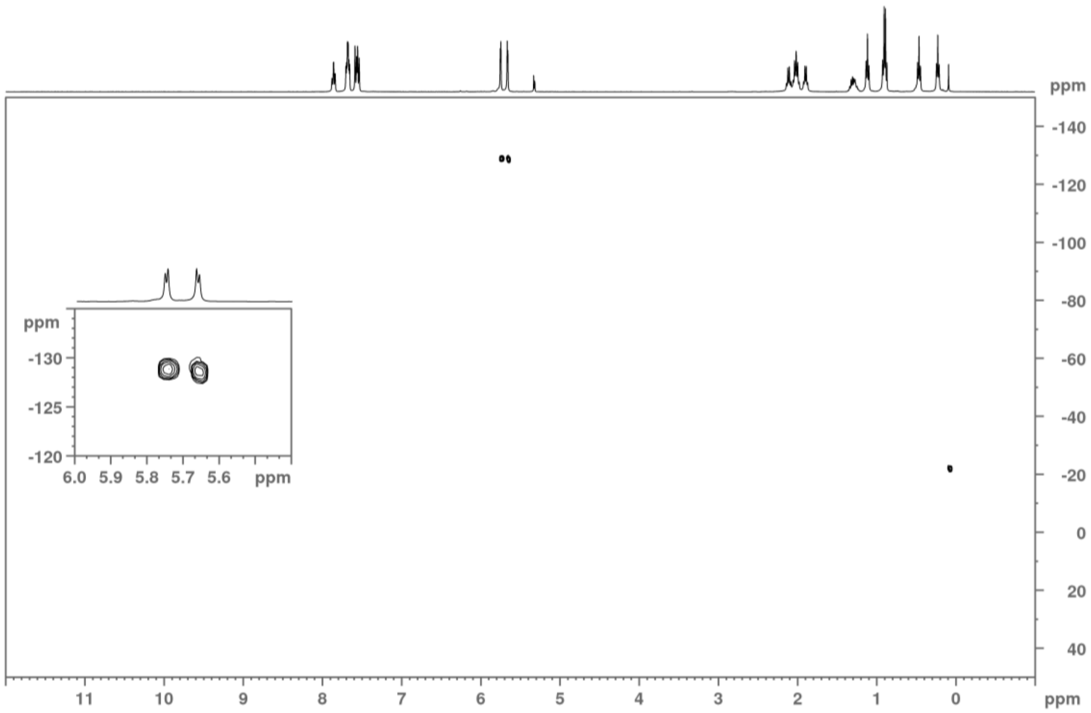

Figure S 7: ^{29}Si HMBC NMR (DCM-d_2, 400 MHz, 295 K) spectrum of [PPh$_4$][**1**]. Cross peak at δ_{29Si} = -23 ppm belongs to H grease.

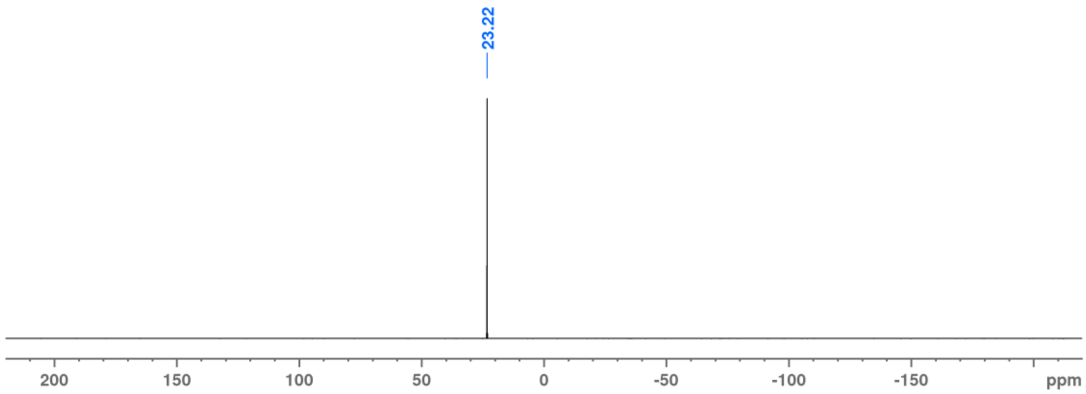

Figure S 8: ^{31}P NMR (DCM-d_2, 161 MHz, 295 K) spectrum of [PPh$_4$][**1**].

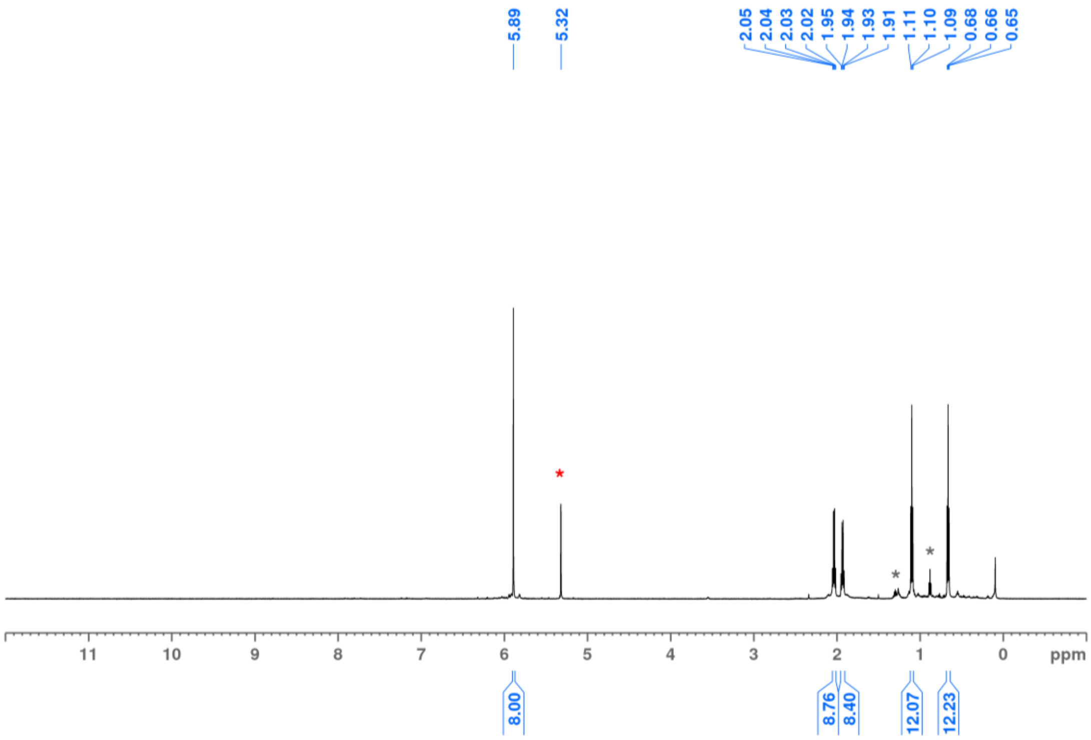

Figure S 9: ^{1}H NMR (DCM-d_2, 400 MHz, 295 K) spectrum of **2.** Gray asterisks mark residues of *n*-pentane.

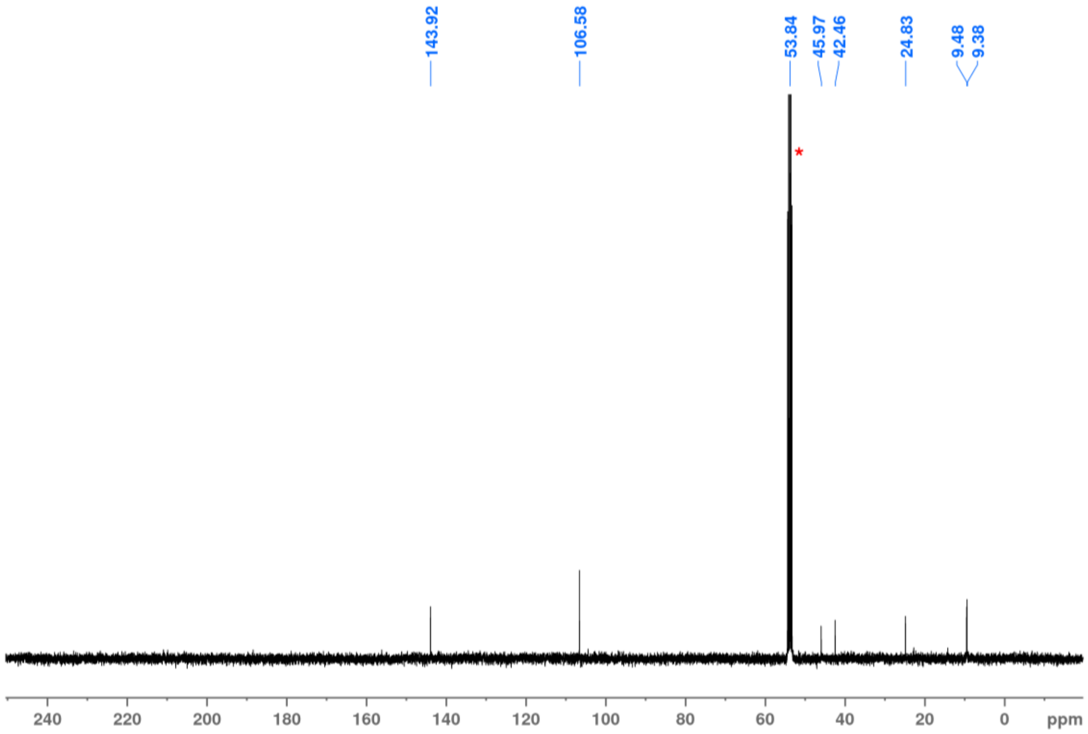

Figure S 10: ^{13}C NMR (DCM-d_2, 100 MHz, 295 K) spectrum of **2.**

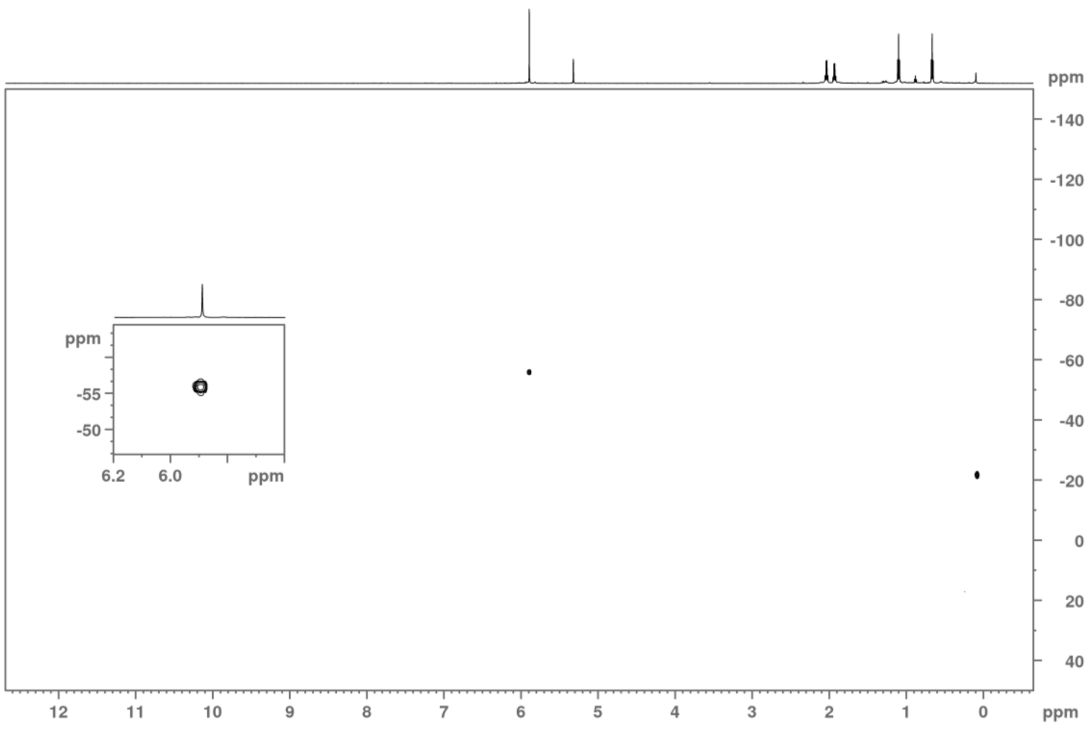

Figure S 11: ^{29}Si HMBC NMR (DCM-d$_2$, 400 MHz, 295 K) spectrum of **2**. Cross peak at δ_{29Si} = -23 ppm belongs to H grease.

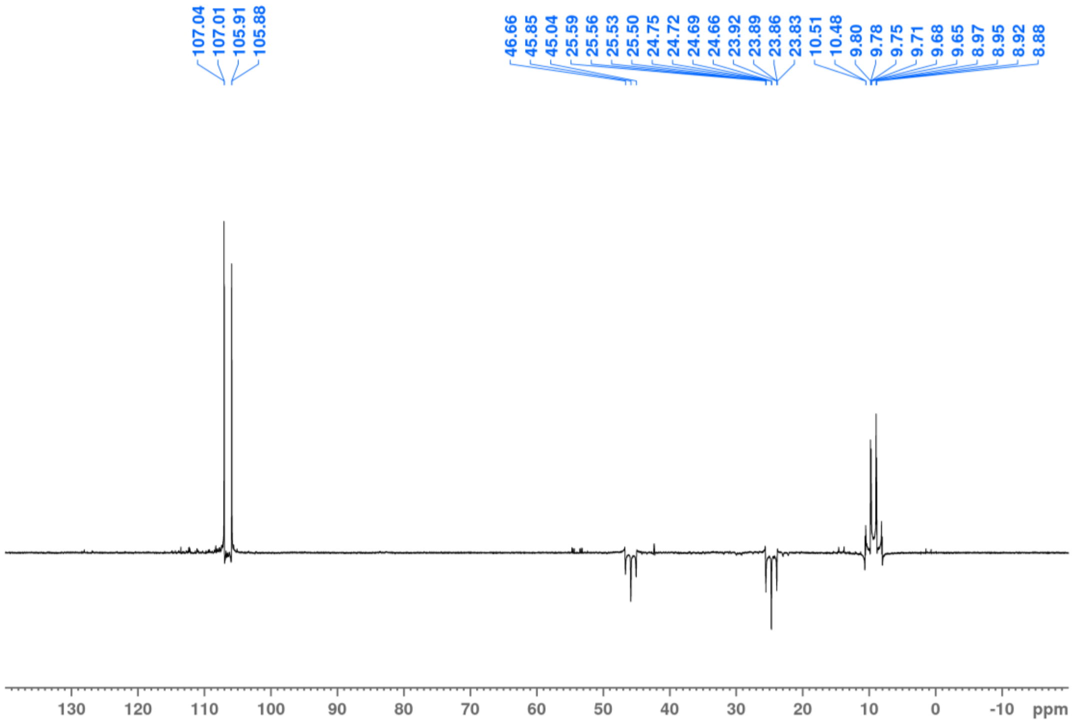

Figure S 12: ^{13}C DEPT135 ^{1}H coupled NMR spectrum (DCM-d$_2$, 150 MHz, 295 K) of **2**.

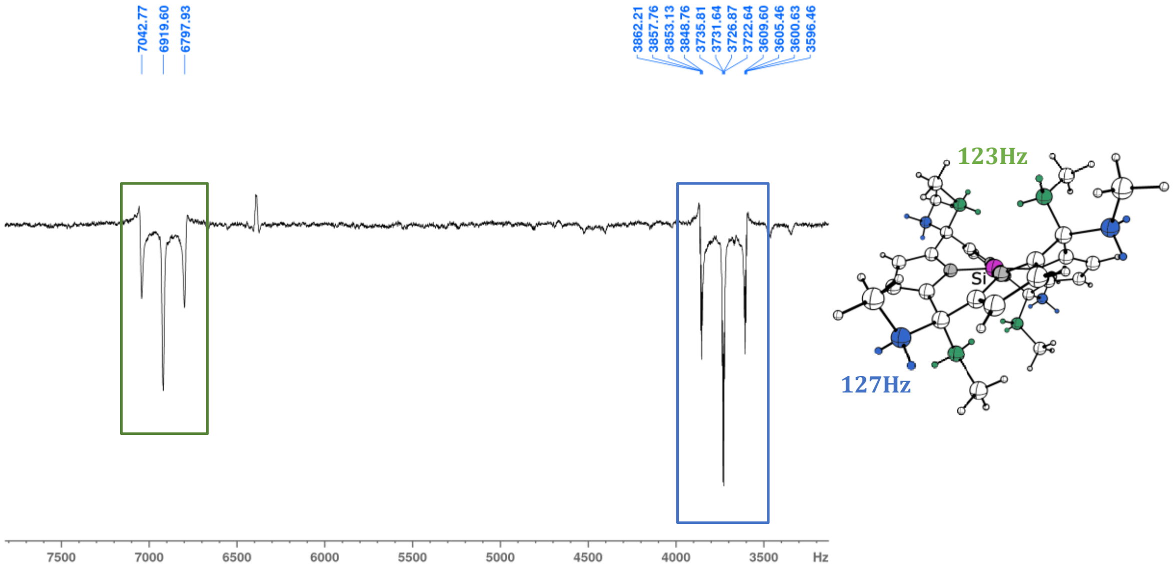

Figure S 13: Section of relevant peak splitting of the ^{13}C DEPT135 ^{1}H coupled NMR spectrum (DCM-d_2, 150 MHz, 295 K) of **2** shown in Figure S 12. Green color marks the endo-methylene group signals, blue color marks the exo-methylene group signals. Difference in coupling constant between exo- and endo-methylene groups is 4 Hz.

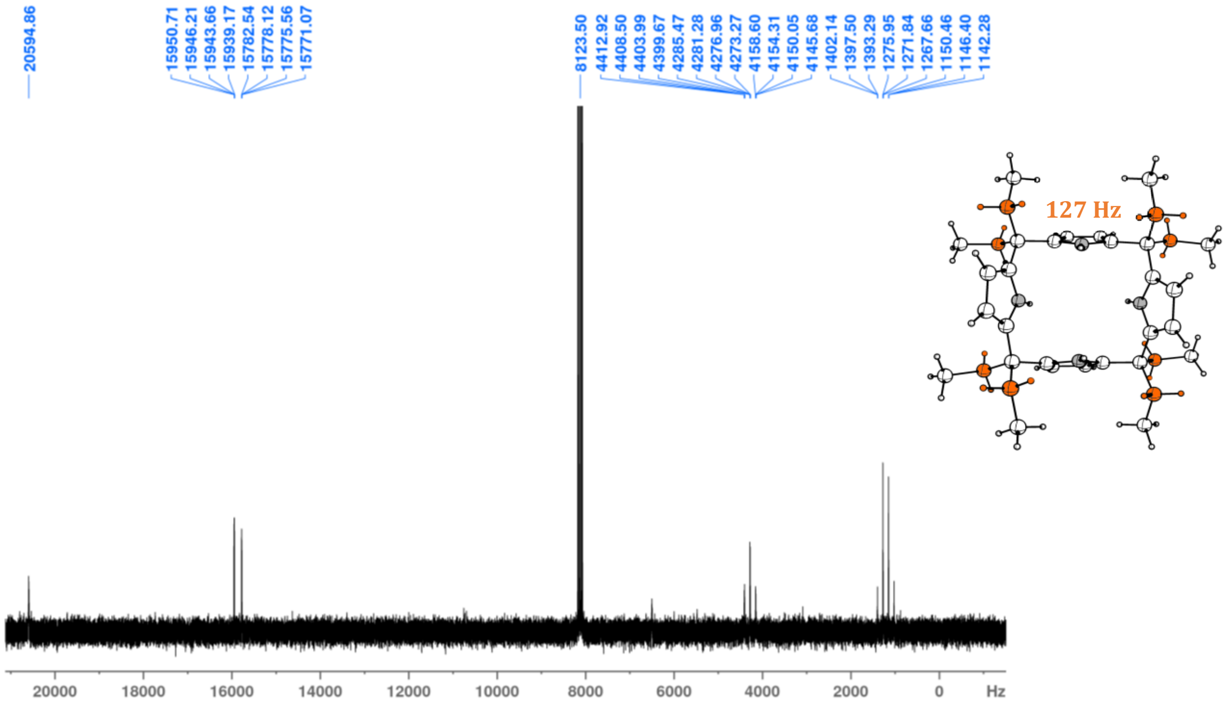

Figure S 14: ^{13}C ^{1}H coupled NMR spectrum (DCM-d_2, 150 MHz, 295 K) of free octaethyl-calix[4]pyrrole ligand.

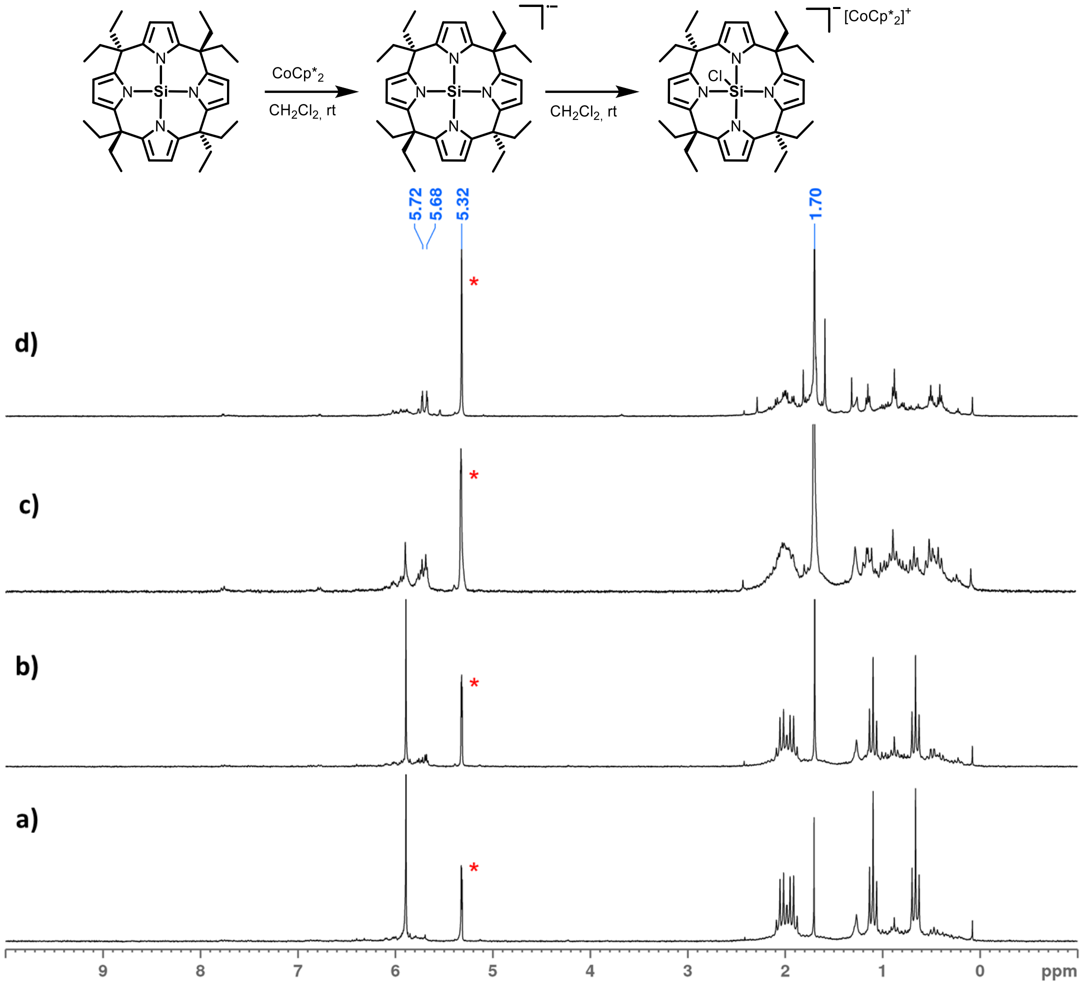

Figure S 15: ^{1}H NMR stack (DCM-d$_2$, 200 MHz, 295 K) of reduction progress after dddition of a) 0.1 eq, b) 0.2 eq, c) 0.6 eq and d) 1.0 eq of CoCp$_2^*$ to **2**. Formation of [**1**]$^-$ as the major product was indicated by the signals at δ = 5.7 ppm.

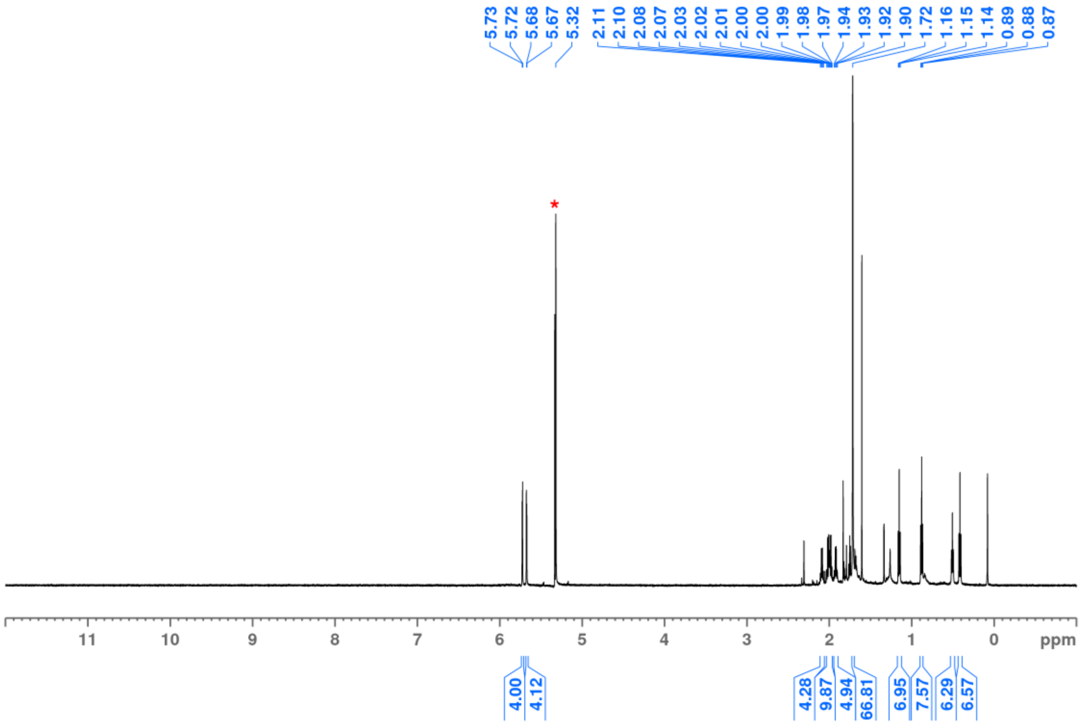

Figure S 16: ^{1}H NMR spectrum (DCM-d_2, 600 MHz, 295 K) of **2** and a slight excess of decamethylcobaltocene in DCM-d_2 at room temperature showing the selective formation of the chlorido silicate [$CoCp_2$*][**1**].

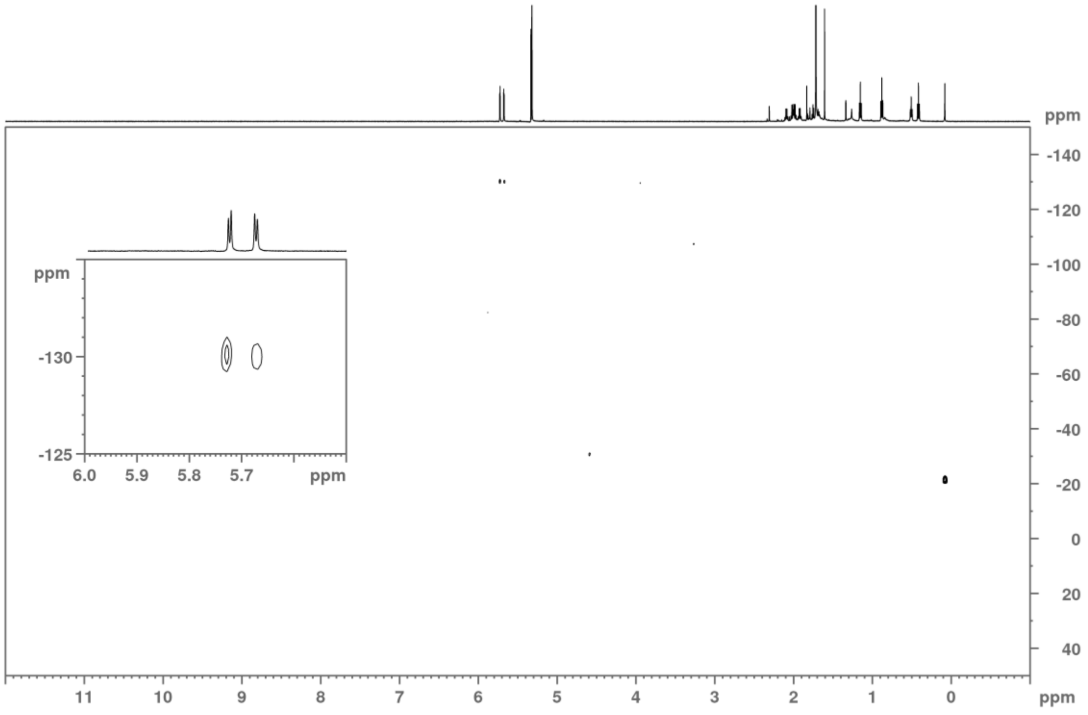

Figure S 17: ^{29}Si HMBC (DCM-d_2, 600 MHz, 295 K) spectrum of [$CoCp_2$*][**1**] formed by the reduction of **2** with decamethylcobaltocene in DCM-d_2.

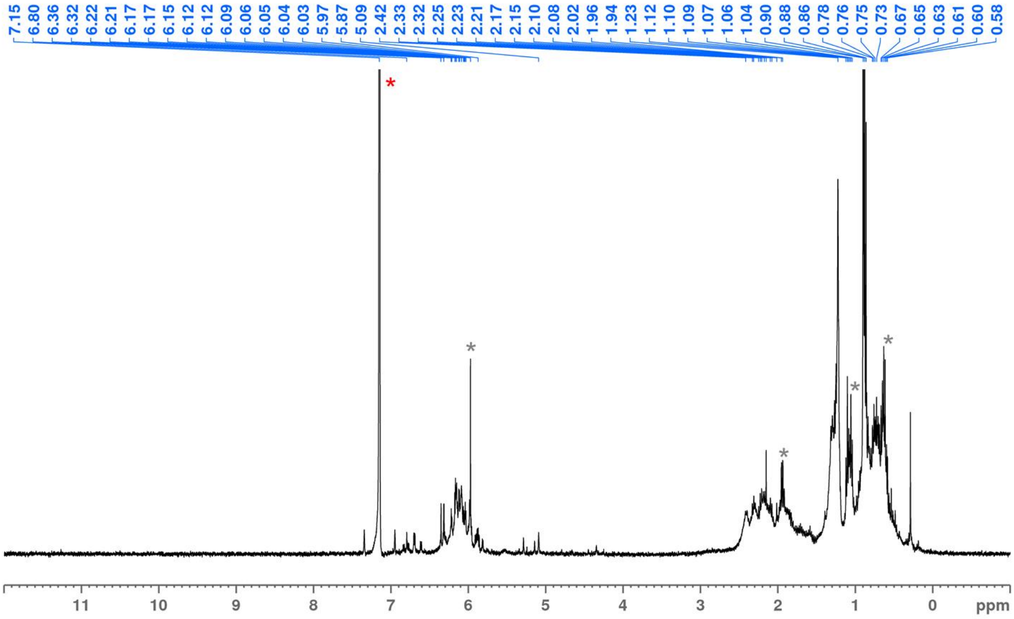

Figure S 18: ^{1}H NMR (C_6D_6, 600 MHz, 295 K) spectrum of **2** and slight shortage of decamethylcobaltocene in C_6D_6 at room temperature showing the successful reduction and formation of various unidetified products.Gray asterisks mark residual **2**.

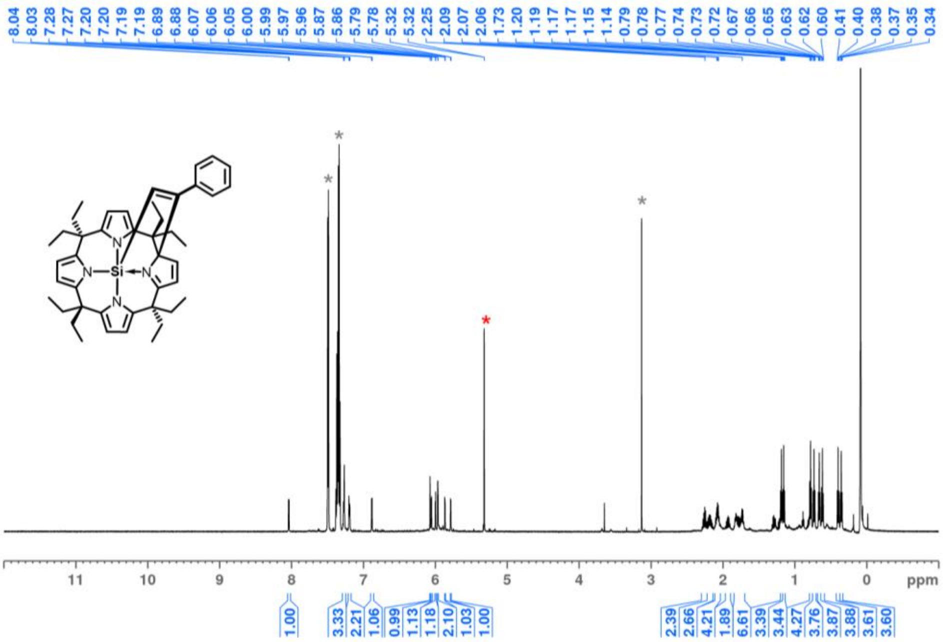

Figure S 19: ^{1}H NMR (DCM-d_2, 600 MHz, 295 K) spectrum of the phenylacetylene activation product by the planar silane **2**. Gray asterisks mark some excess of substrate.

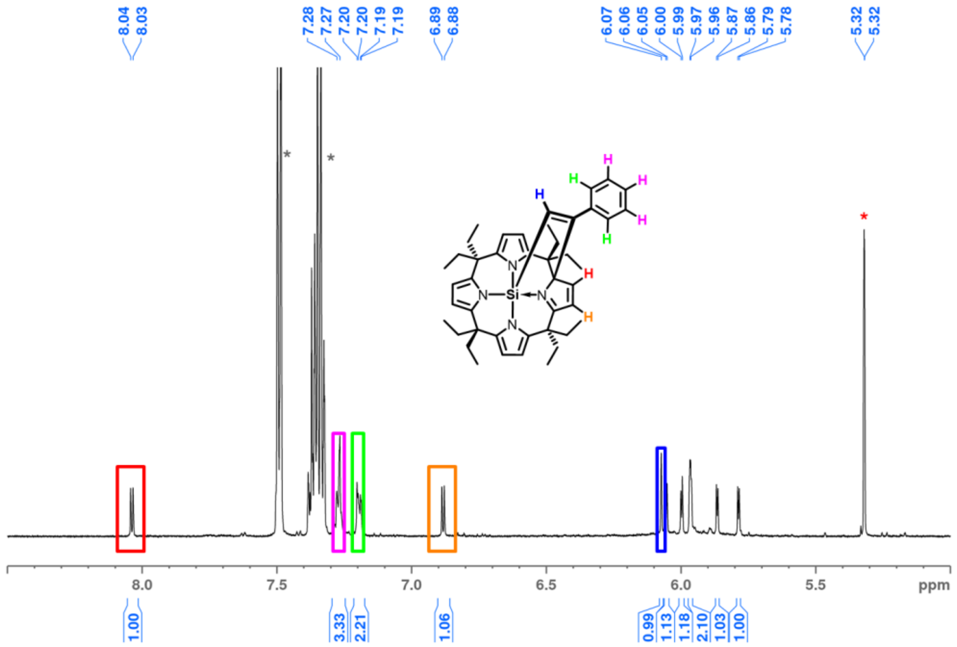

Figure S 20: Zoomed in area of the ^{1}H NMR (DCM-d_2, 600 MHz, 295 K) spectrum of the phenylacetylene activation product by the planar silane **2** showing the characteristic aromatic area and characteristic proton signals indicated by colors. Gray asterisks mark some excess of substrate.

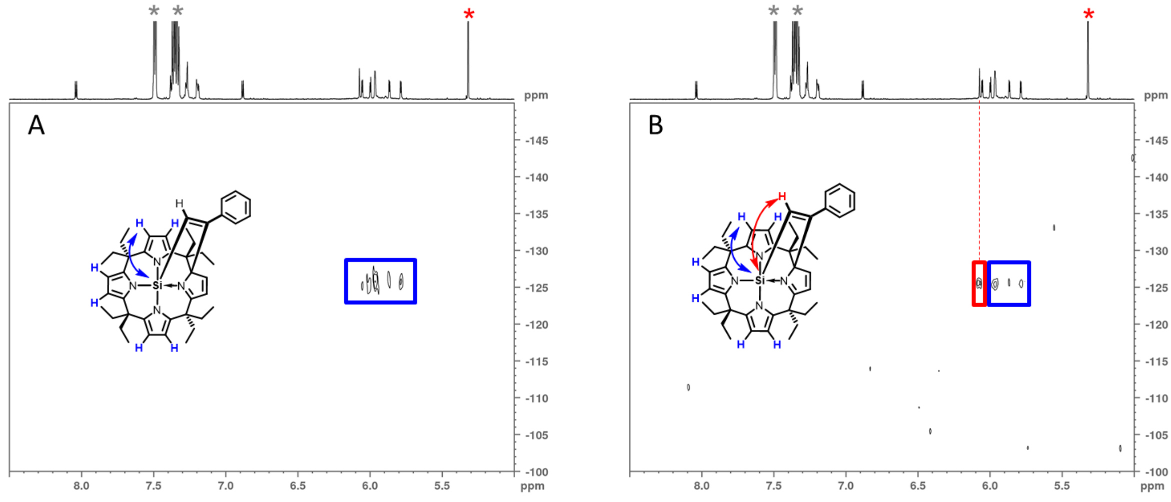

Figure S 21: ^{29}Si HMBC NMR (DCM-d_2, 600 MHz, 295 K) spectrum of the activation product of phenylacetylene by the planar silane **2**. The ^{29}Si shift of the activated species is δ = -126.0 ppm. A) Spectrum was measured with cnst13 = 3 Hz; B) Spectrum was measured with cnst13 = 7 Hz. Gray asterisks mark some excess of substrate.

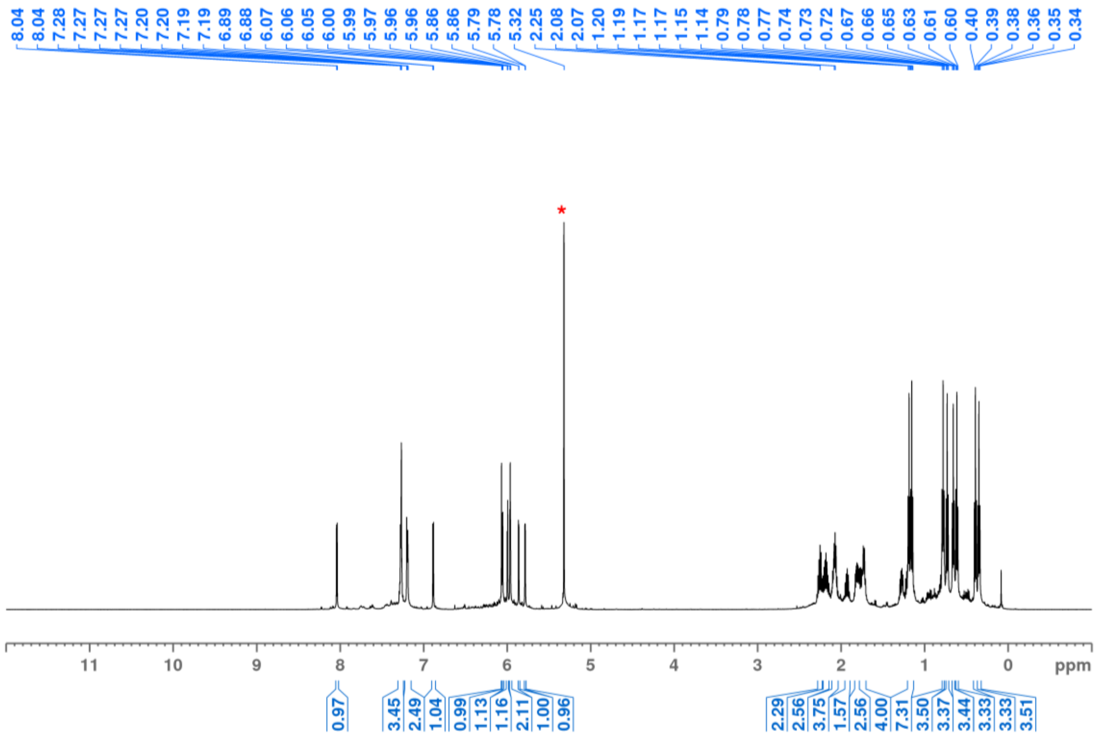

Figure S 22: ^{1}H NMR (DCM-d_2, 600 MHz, 295 K) spectrum of the isolated phenylacetylene activation product by the planar silane **2**.

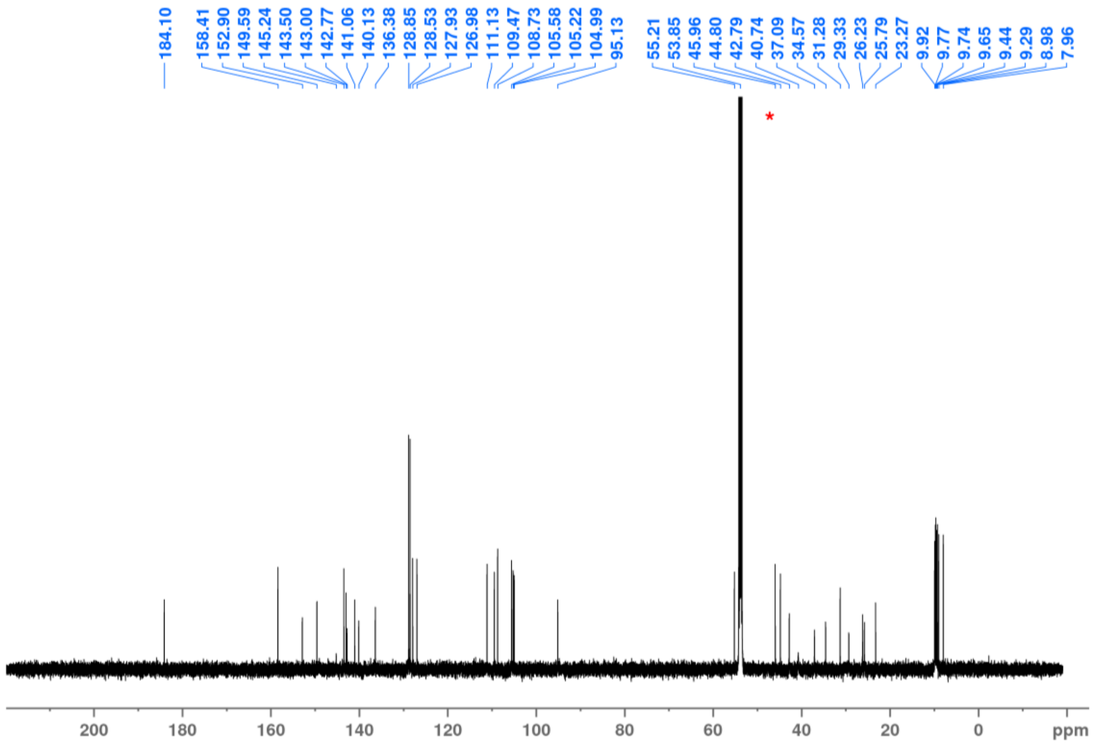

Figure S 23: ^{13}C NMR (DCM-d_2, 150 MHz, 295 K) spectrum of the isolated phenylacetylene activation product by the planar silane **2**.

Single crystal X-ray diffraction

Suitable crystals for single-crystal structure determination were taken directly from the mother liquor, covered with perfluorinated polyether oil and fixed on a cryo loop. Full shells of intensity data were collected at low temperature with a Bruker D8 Venture diffractometer, dual source (Mo-or Cu-K_α radiation, microfocus X-ray tube, Photon III detector). Data were processed with the standard Bruker (SAINT, APEX3) software package.[1, 2] Multiscan absorption correction was applied using the SADABS program.[3, 4] The structures were solved by intrinsic phasing[5, 6] and refined using the SHELXTL software package (Version 2018/3).[7-10] Graphical handling of the structural data during solution and refinement was performed with OLEX2.[11]

For the ShelXL SCRXD structure refinement of **2**, all hydrogen atoms were found in the residual density map and refined isotropically without restraints. All non-hydrogen atoms were given anisotropic displacement parameters.

The structure solution of **2** was further improved by Hirshfeld atom refinement,[12, 13] as implemented by the NoSphereA2 module in Olex2 version 1.3 alpha.[14] The method provides aspherical atomic electron densities created from the crystal-field embedded quantum-chemically derived electron density using the Hirshfeld partitioning. In the present case, the quantum chemically derived electron density was obtained at the PBE0/def2-TZVPP level of theory from ORCA. Coordinates of all atoms, including hydrogen atoms, were refined freely and anistropically. Hence, also for the hydrogen atoms, aspherical displacement parameters were obtained.

Crystallographic data for the structures reported in this article are deposited within the Cambridge Crystallographic Data Centre as supplementary publication no. CCDC 2042938-2042941 and can be obtained free of charge. For compound **2**, both the ShelXL refinement solution (CCDC 2042941) and the NoSphereA2 refinement solution ($\mathbf{2}_{\mathbf{SCXRD\text{-}HAR}}$, CCDC 2042939) were deposited. Crystal data and structure are summarized in the tables under the crystallographic figure.

Additional proof for the correct determination of the hydrogen atom positions via SCXRD/NoSpherA2 HAR method was obtained by DFT-theoretical reoptimization of the H-atom positions with PBEh-3c (see computational section for further details), starting from the $\mathbf{2}_{\mathbf{SCXRD\text{-}HAR}}$ refined structure. A shifting of max 2 pm was observed for the protons, as shown in table S1, which compares C-H bond lengths for the methylene groups.

Table S 1: Comparison of C-H bond lengths [pm] of **2** obtained by SCXRD and subsequent HAR with NoSpherA2 (PBE0/def2-TZVPP) and the “ab initio” results computed by PBEh-3c starting from the conventionally refined SCXRD structure.

	SCXRD-HAR	PBEh-3c	deviation
C27-H27b (in)	110.9	109.70	-1.2
C27-H27a (out)	109.8	109.30	-0.5
C35-H35b (in)	110.0	109.40	-0.6
C35-H35b (out)	110.1	109.40	-0.7
C29-H29b (in)	112.1	109.80	-2.3
C29-H29a (out)	108.1	109.20	1.1
C21-H21b (in)	110.2	109.30	-0.9
C21-H21b (out)	111.3	109.30	-2.0

Figures of molecular structures

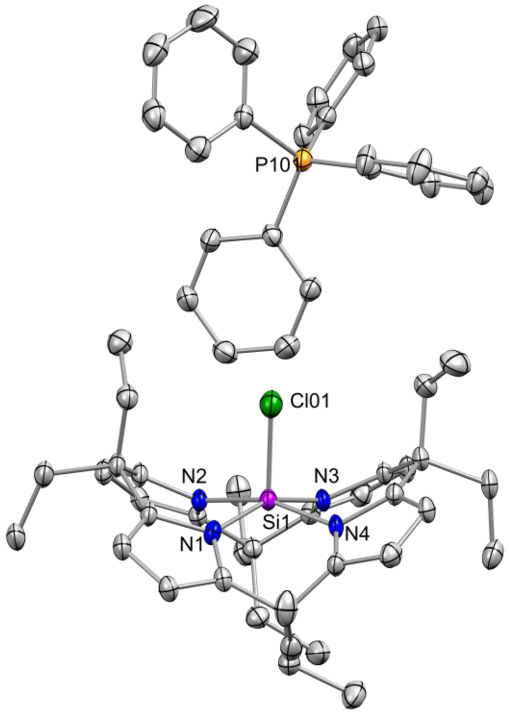

Figure S 24: Molecular structure of [**1**][PPh_4]. Hydrogen atoms are omitted for clarity. Displacement ellipsoids are drawn with a probability of 50 %. Selected bond distances (pm): Si(1)-N(1) 187.0(3), Si(1)-N(2) 186.6(3), Si(1)-N(3) 185.5(3), Si(1)-N(4) 185.0(3), Si(1)-Cl(01) 206.82(13). Selected bond angles (deg): N(1)-Si(1)-N(3) 157.23(13), N(2)-Si(1)-N(4) 161.52(13).

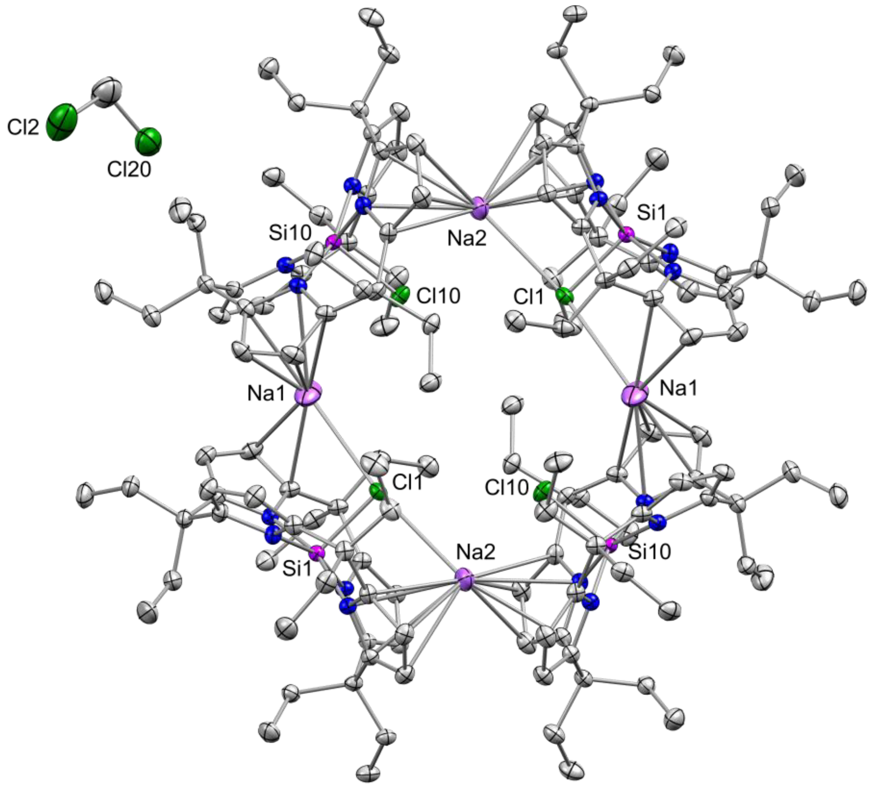

Figure S 25: Molecular structure of [**1**]$_4$[Na]$_4$. Hydrogen atoms are omitted for clarity. Displacement ellipsoids are drawn with a probability of 50 %. Two cell units are shown. For structural parameter see Figure S 26.

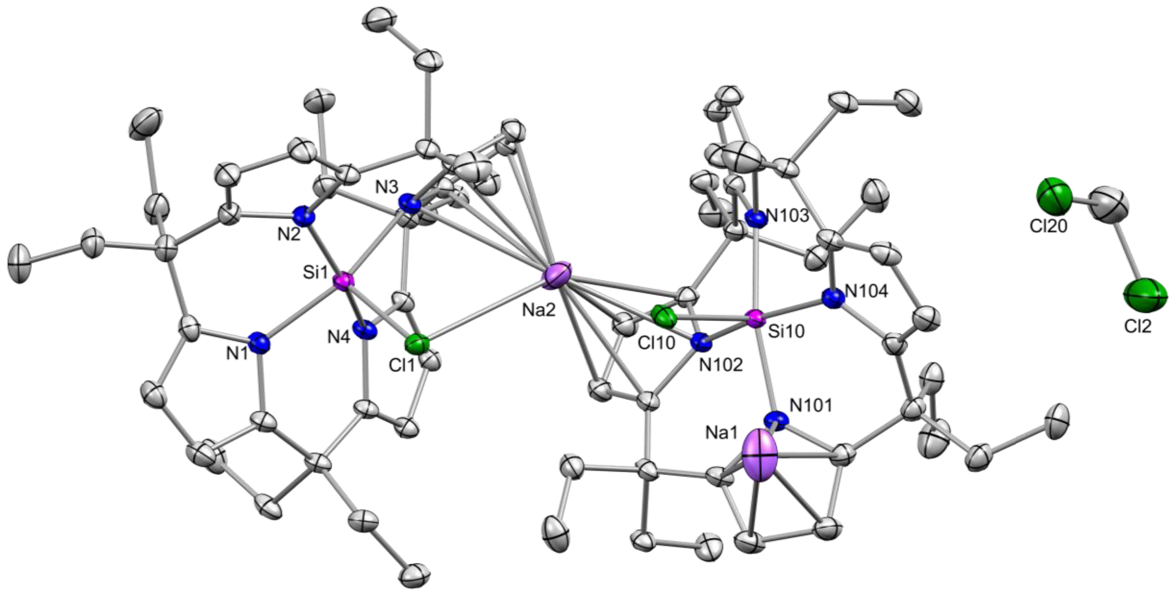

Figure S 26: Part of the molecular structure of [**1**]$_4$[Na]$_4$. Hydrogen atoms are omitted for clarity. Displacement ellipsoids are drawn with a probability of 50 %. Selected bond distances (pm): Si(1)-N(1) 186.15(13), Si(1)-N(2) 183.89(13), Si(1)-N(3) 186.53(13), Si(1)-N(4) 184.85(14), Si(1)-Cl(1) 213.47(6), Na(2)-Cl(1) 309.18(10), Si(10)-N(101) 185.72(13), Si(10)-N(102) 185.95(13), Si(10)-N(103) 182.73(13), Si(10)-N(104) 183.12(13), Si(10)-Cl(10) 213.5(2), Na(1)-Cl(10) 335.0(4). Selected bond angles (deg): N(1)-Si(1)-N(3) 163.40(6), N(2)-Si(1)-N(4) 162.38(7), N(101)-Si(1)-N(103) 163.58(6), N(102)-Si(1)-N(104) 162.13(6).

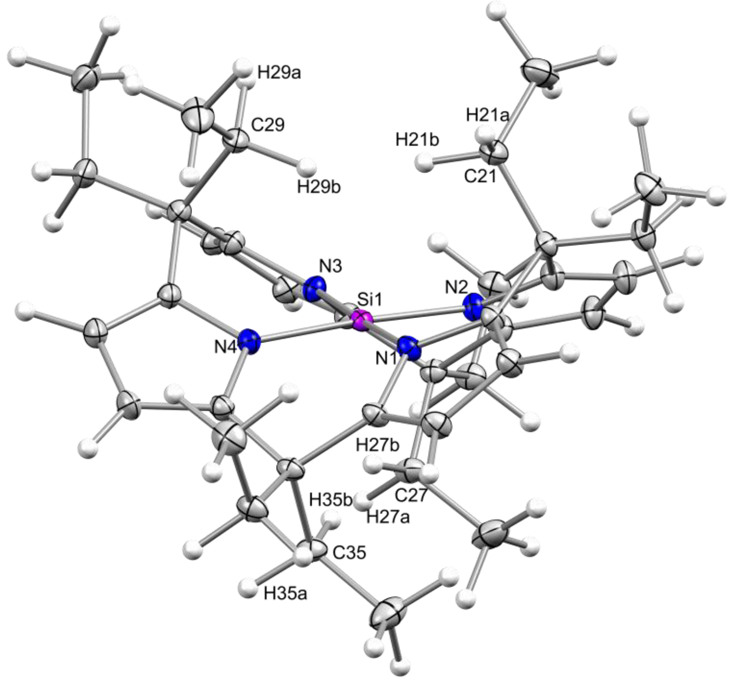

Figure S 27: Molecular structure of **2** (HAR). Displacement ellipsoids are drawn with a probability of 50 %. Selected bond distances (pm): Si(1)-N(1) 178.84(5), Si(1)-N(2) 179.26(5), Si(1)-N(3) 179.90(4), Si(1)-N(4) 178.81(4), C(29)-H(29a) 108.1, C(29)-H(29b) 112.1, C(21)-H(21a) 111.2, C(21)-H(21b) 110.2, C(27)-H(27a) 109.8, C(27)-H(27b) 111.0, C(35)-H(35a) 110.1, C(35)-H(35b) 109.9. Selected bond angles (deg): N(1)-Si(1)-N(3) 178.19(2), N(2)-Si(1)-N(4) 176.08(2), N(1)-Si(1)-N(2) 90.15(2), N(2)-Si(1)-N(3) 89.28(2), N(3)-Si(1)-N(4) 90.43(2), N(4)-Si(1)-N(1) 90.26(2).

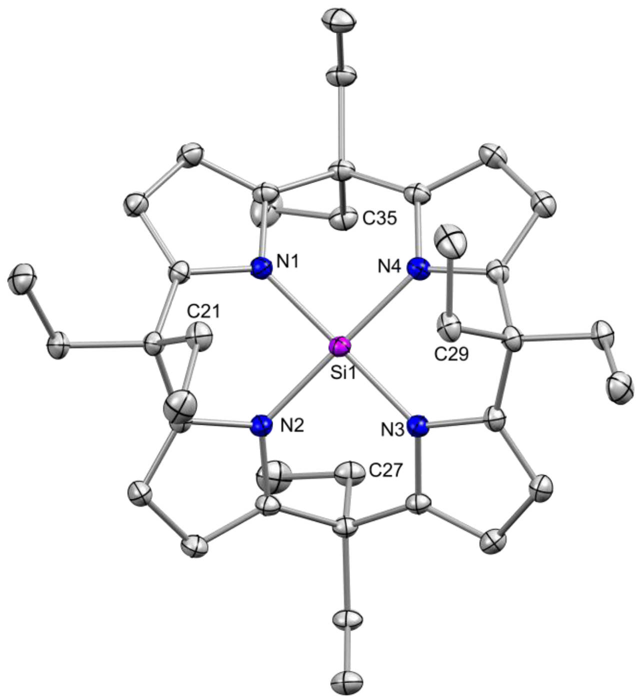

Figure S 28: Top-down view of molecular structure of **2**. Hydrogen atoms are omitted for clarity. Displacement ellipsoids are drawn with a probability of 50 %. For structural parameter see Figure S 27.

Table S 2: Crystallographic data

Compound	[PPh_4][**1**]	$[Na]_4[\mathbf{1}]_4$	**2** (refined with ShelXL)	**2** (refined with NoSphereA2)
Identification code	mo_fe744_0m	mo_fe754_01	mo_fe736_0m	mo_fe736_0m_NoSpherA2
Empirical formula	$C_{60}H_{68}ClN_4PSi$	$C_{73}H_{98}Cl_4N_8Na_2Si_2$	$C_{36}H_{48}N_4Si$	$C_{36}H_{48}N_4Si$
Formula weight	939.69	1331.55	564.87	564.895
Temperature/K	100.0	100.0	100.0	100.0
Crystal system	triclinic	triclinic	monoclinic	monoclinic
Space group	P-1	P-1	$P2_1/n$	$P2_1/n$
a/Å	12.0514(9)	14.099(2)	10.3052(4)	10.3052(4)
b/Å	13.9832(10)	15.651(2)	19.3263(7)	19.3263(7)
c/Å	16.6437(13)	16.334(3)	14.9876(7)	14.9876(7)
α/°	78.997(3)	77.122(6)	90	90
β/°	73.403(3)	84.014(6)	94.396(2)	94.396(2)
γ/°	69.275(3)	76.639(6)	90	90
Volume/$Å^3$	2501.3(3)	3413.1(9)	2976.2(2)	2976.2(2)
Z	2	2	4	4
$\rho_{calc}g/cm^3$	1.248	1.296	1.261	1.261
μ/mm^{-1}	0.177	0.271	0.112	0.112
F(000)	1004.0	1420.0	1224.0	1224.7
Crystal size/mm^3	0.135 × 0.116 × 0.102	0.12 × 0.103 × 0.095	0.191 × 0.189 × 0.184	0.191 × 0.189 × 0.184
Radiation	MoKα (λ = 0.71073)	MoKα (λ = 0.71073)	MoKα (λ = 0.71073)	Mo Kα (λ = 0.71073)
2Θ range for data	3.72 to 53.996	3.816 to 57.998	4.636 to 61.09	4.64 to 61.1
Index ranges	-15 ≤ h ≤ 15, -17 ≤ k ≤ 17, -	-19 ≤ h ≤ 19, -21 ≤ k ≤ 21, -22	-14 ≤ h ≤ 14, -27 ≤ k ≤ 27, -21 ≤ l	-14 ≤ h ≤ 14, -27 ≤ k ≤ 27, -21 ≤ l
Reflections collected	75362	201842	113758	113758
Independent reflections	10910 [R_{int} = 0.1784,	18139 [R_{int} = 0.0464, R_{sigma} =	9086 [R_{int} = 0.0505, R_{sigma} =	9082 [R_{int} = 0.0505, R_{sigma} =
Data/restraints/parameter	10910/0/612	18139/0/828	9086/0/406	9082/0/802
Goodness-of-fit on F^2	1.017	1.039	1.051	1.089
Final R indexes [I>=2σ (I)]	R_1 = 0.0650, wR_2 = 0.1338	R_1 = 0.04446, wR_2 = 0.1131	R_1 = 0.0380, wR_2 = 0.0979	R_1 = 0.0203, wR_2 = 0.0399
Final R indexes [all data]	R_1 = 0.1321, wR_2 = 0.1679	R_1 = 0.0528, wR_2 = 0.1195	R_1 = 0.0459, wR_2 = 0.1046	R_1 = 0.0280, wR_2 = 0.0436
Largest diff. peak/hole / e	0.52/-0.64	1.00/-0.76	0.42/-0.29	0.24/-0.16
CCDC Database number	2042938	2042940	2042941	2042939

ATR-IR spectra

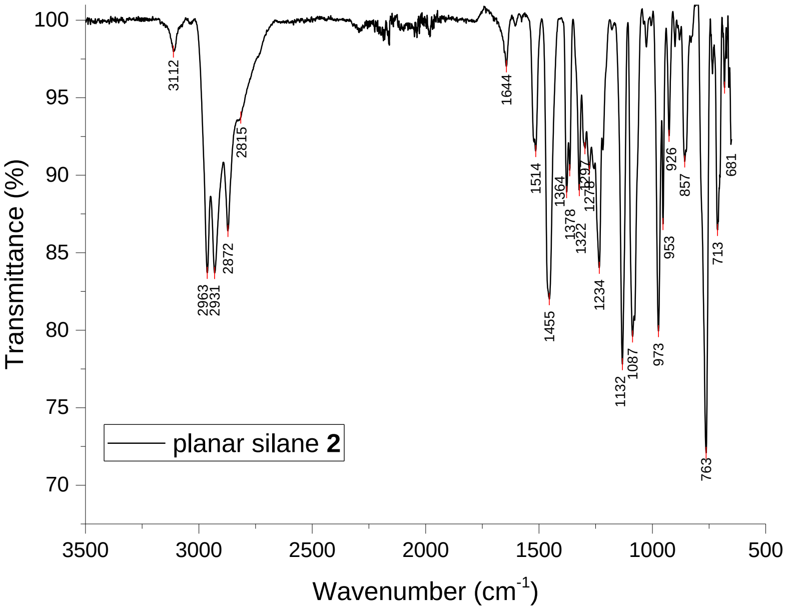

Figure S 29: IR spectrum of **2**.

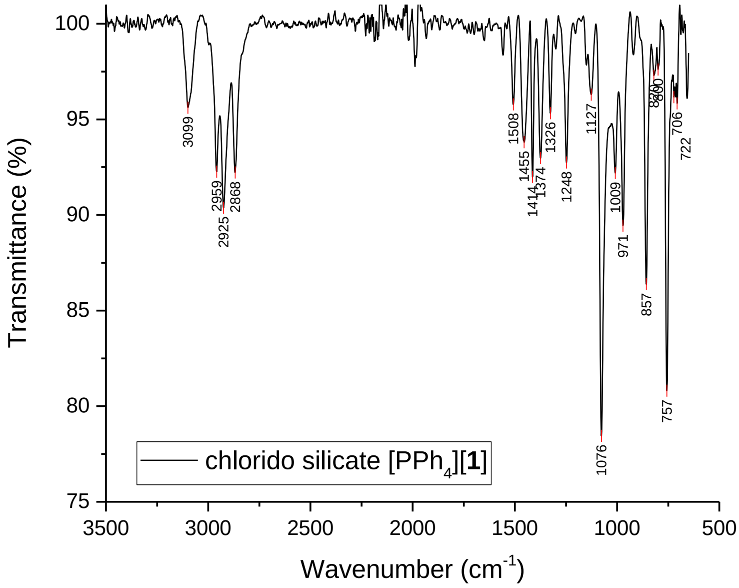

Figure S 30: IR spectrum of [PPh$_4$][**1**].

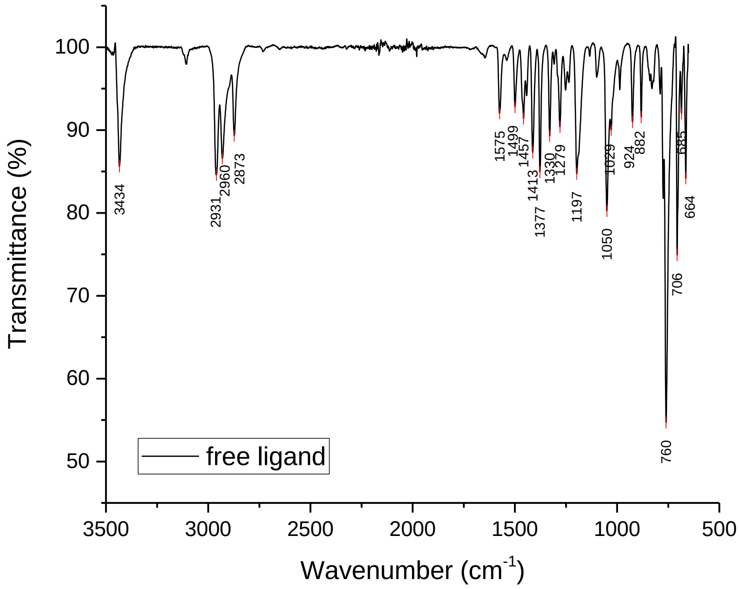

Figure S 31: IR spectrum of free *meso*-Octaethylcalix[4]pyrrole ligand as reference.

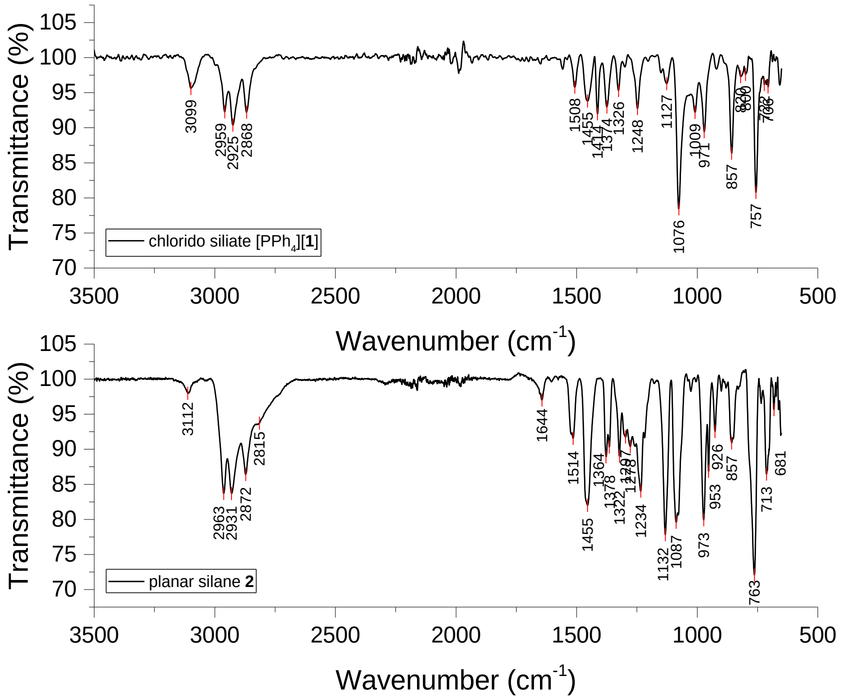

Figure S 32: Comparison of the IR spectra of [PPh4][**1**] and **2**. The shoulder at 2815 cm^{-1} indicates the agostic interaction in the planar silane **2**.

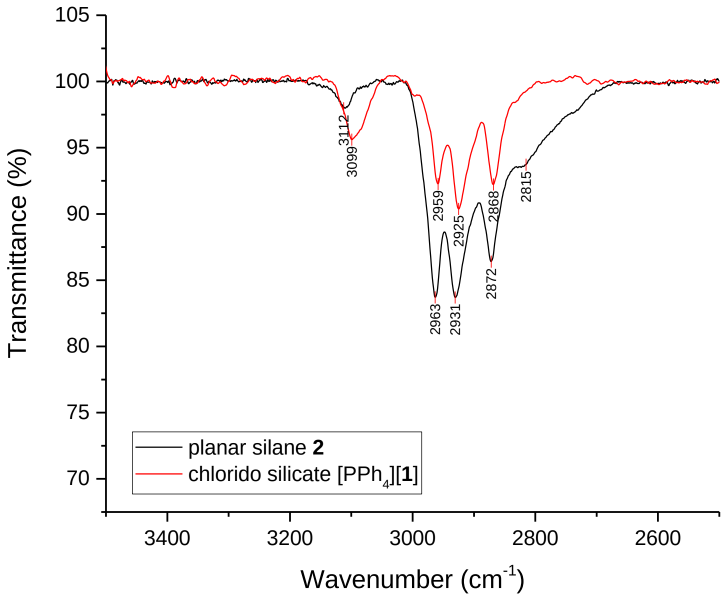

Figure S 33: Zoom of Figure S 32 from 3500 – 2500 cm^{-1} showing the band at 2815 cm^{-1} assigned to the agostic interactions in the planar silane **2**.

UV-Vis spectra

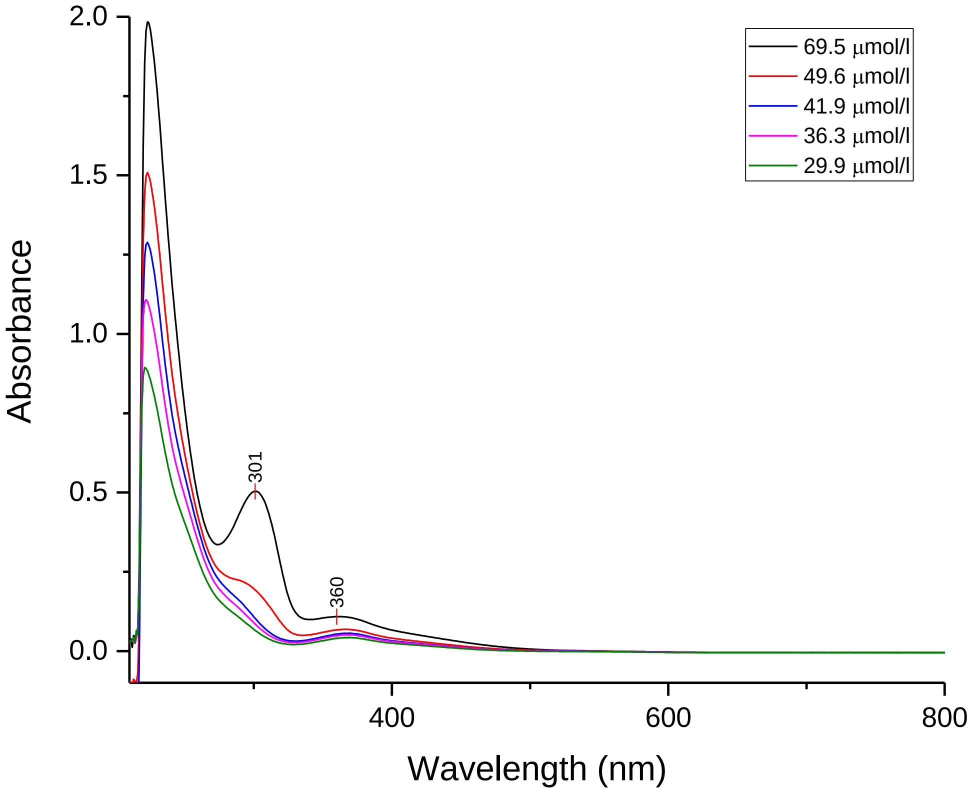

Figure S 34: UV-Vis spectrum of **2** in DCM with d = 0.2 cm.

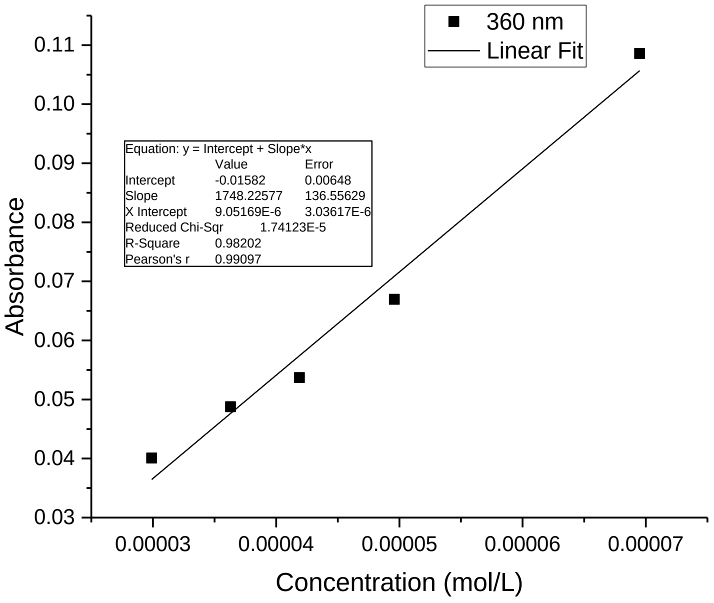

Figure S 35: Determination of the extinction coefficient of **2** from data of Figure S 34: ε(360 nm) = 8740 ± 680 L mol^{-1} cm^{-1}.

CV measurements

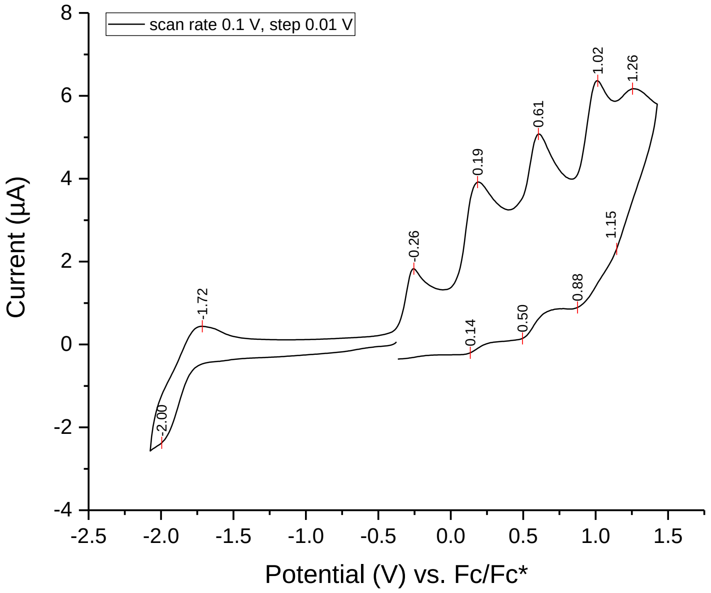

Figure S 36: CV experiment of **2** (1 mM) in DCM with 0.1 V/s NBu_4BArF_{20} (0.1 M).

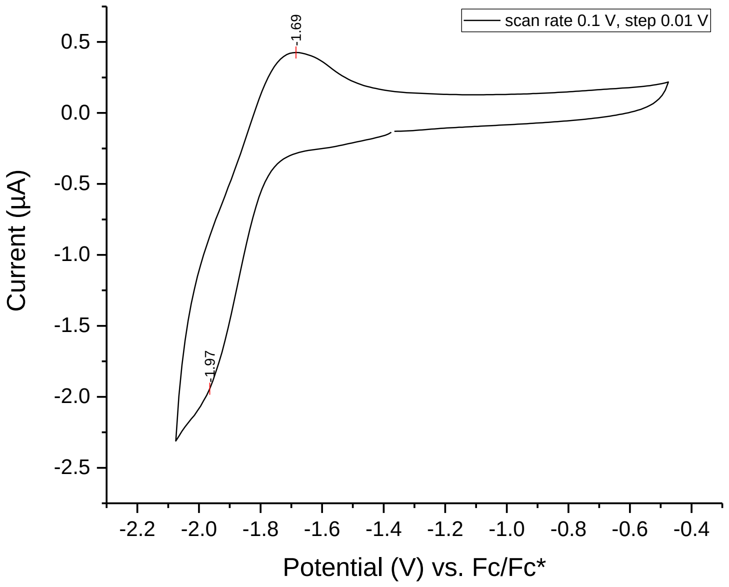

Figure S 37: CV experiment of **2** (1 mM) in DCM with 0.1 V/s NBu_4BArF_{20} (0.1 M).

Supplemental Computational Data

For all quantum chemical calculations, Orca 4.1.2 or 4.2.1[15] was employed. Ball and stick representations were rendered with Chemcraft 1.8 or IBOView.[16]

Structure optimization

All structures were optimized with the PBEh-3c model[17] and confirmed to possess only positive Hessian matrix eigenvalues. PBEh-3c is a composite electronic structure method based on a hybrid Perdew-Burke-Ernzerhoff (PBE) exchange-correlation functional combined with polarized valence-double zeta Gaussian atomic orbital basis sets (def2-mSVP). It treats basis set superposition errors with the geometrical counterpoise scheme (gCP)[18], and London dispersion interactions with the Becke-Johnson-damped D3 correction[19, 20]. PBEh-3c produces results of similar structural accuracy to those obtained with MP2/def2-TZVPP.[21] Comparison of selected computed bond lengths with the SCXRD derived data of **2** and **3** is presented in Table S 3. It illustrates the very good performance of the computational model, with a maximum bond length deviation of 1.7 pm and bond angle deviation of 1° for **2** and **3** (Si(pyrrole)$_4$).

Table S 3: Comparison of PBEh-3c computed metric parameters with SCXRD-HAR derived values of **2** and the literature SCXRD values of **3**.[22]

bond length [pm]	**PBEh-3c**	**SCXRD**	**Deviation**
bond angles (°)			
**[2]**			
Si-N1	180.11	178.85	1.3
Si-N2	179.93	179.26	0.7
Si-N3	180.12	179.90	0.2
Si-N4	179.89	178.82	1.1
N1-C1	139.43	141.11	-1.7
C1-C2	135.99	136.49	-0.5
N1-Si-N3	177.0	178.2	-1.2
N1-Si-N2	175.3	176.1	-0.8
**[3]**		**SCXRD[22]**	
Si-N1	173.62	172.50	1.12
N1-C1	138.10	139.30	-1.20
C1-C2	136.10	135.70	0.40
N1-Si-N2	111.0	110.5	0.50

Figure S 38 shows the computed PBEh-3c minimum structure of **2**, with (unscaled) IR stretching frequencies of the assigned C-H bonds (major contributions). Although the values are systematically shifted compared to the experimental data, they show the same trend → the smallest for the "agostic" C-H-bonds (endo-in).

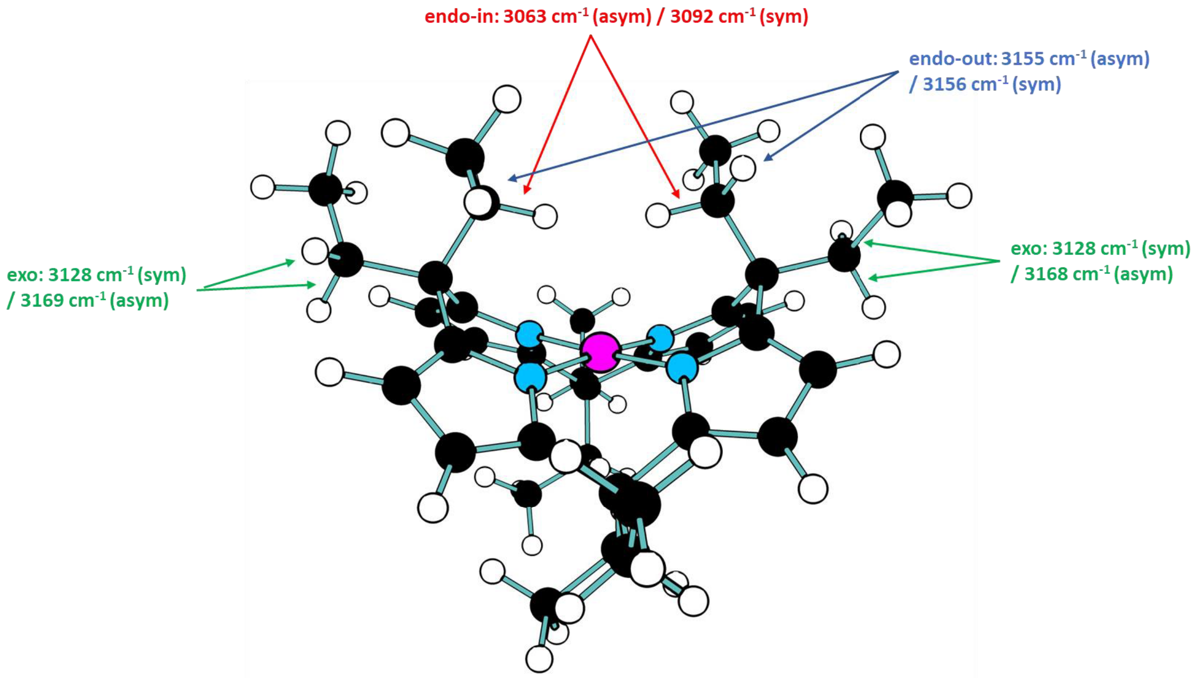

Figure S 38: Computed PBEh-3c minimum structure of **2** and the computed vibrational frequencies.

Single point computation for electron affinities and frontier molecular energies

The gas phase single point computations for the discussion of frontier molecular orbital energies and electron affinities were calculated with the hybrid meta exchange-correlation functional PW6B95-D3(BJ), that was shown to be very reliable for the computation of electron affinities,[23] in combination with the extensive def2-QZVPP basis set.[24] SCF settings were tightSCF and integration was performed at a very fine grid7. The RIJCOSX Fock-matrix formation algorithm was used as implemented in Orca along with the respective automatically generated auxiliary basis sets (AutoAux)[25]. The RIJCOSX scheme combines the chain of spheres exchange approximation (COSX)[26] for the computation of the exchange matrix with the Split-RI-J algorithm[27] for the calculation of the Coulomb matrix. Enthalpies at 298.15 K were calculated with the total thermal and zero-point energy correction from the PBEh-3c calculation combined with the electronic single point energies obtained on the PW6B95-D3(BJ)/def2-QZVPP level of theory.

Table S 4: PBEh-3c derived enthalpy correction and PW6B95-D3(BJ)/def2-QZVPP derived single point energy, enthalpy and FMO energies.

	**Thermal + ZPE correction from PBEh-3c [kJ mol^{-1}]**	**E [Hartree] PW6B95-D3(BJ)/def2-QZVPP**	**E [kJ mol^{-1}]**	**H [kJ mol^{-1}]**	HOMO [eV]	LUMO [eV]
**2**	2199.9	-1912.4490	-5021134.8	-5018935.0	-5.69	-2.3
**2_radical_anion**	2195.3	-1912.5102	-5021295.6	-5019100.3		
**3**	842.6	-1129.6732	-2965956.9	-2965114.3	-6.58	0.14
**3_radical anion**	836.4	-1129.6729	-2965956.1	-2965119.8		

			E_{rad_anion} – $E_{neutral}$	H_{rad_anion} – $H_{neutral}$		
	**Electron Affinity**	**for 2**	160.8	165.3		
		**for 3**	-0.8	5.5		

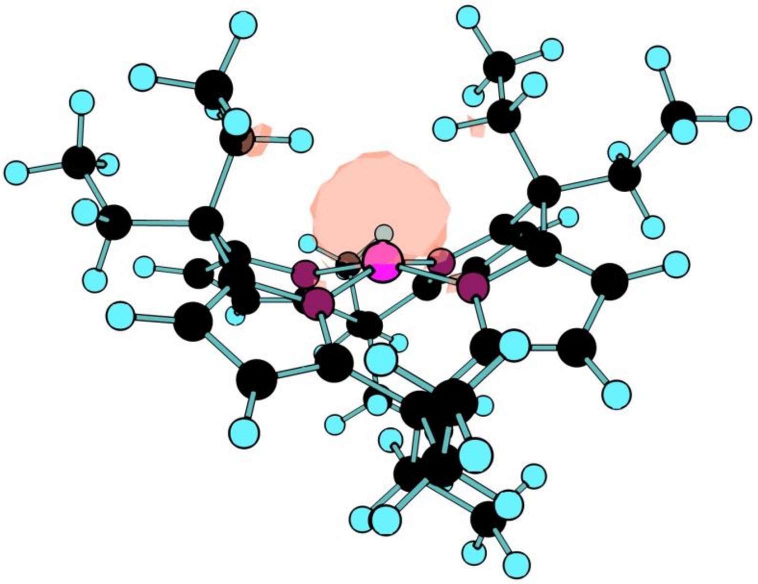

Figure S 39: Spin Density Distribution of **2_radical_anion** (PBEh-3c, isodensity value 0.005 au.

Electron density evaluation of the agostic interaction by NBO and QTAIM

Natural bond orbitals (NBO) [28, 29] or the quantum theory of atoms in molecules (QTAIM) [30] both analyze the electron density distribution. In recent work, it was shown that the hybrid PBE0 functional [31, 32] is among the best performing functionals for the accurate description of the electron density distribution, referenced against exact all-electron coupled-cluster singles and doubles densities.[33] Hence, the wavefunction for the gas-phase PBEh-3c optimized structure [$\mathbf{2}_{\mathbf{PBE\text{-}Gas}}$] was recomputed with PBE0/def2-TZVPP at fine grid6 settings, and analyzed with NBO 7.0 [34] and AIMAll Version 19.10.12.[35] Besides, the wavefunction for the molecular structure obtained by SCXRD + Hirshfeld Atom Refinement, named [$\mathbf{2}_{\mathbf{SCXRD\text{-}HAR}}$], was computed at PBE0/def2-TZVPP and analyzed accordingly. The NBO results of relevance for the description of the agostic interaction can be found in Table S 5.

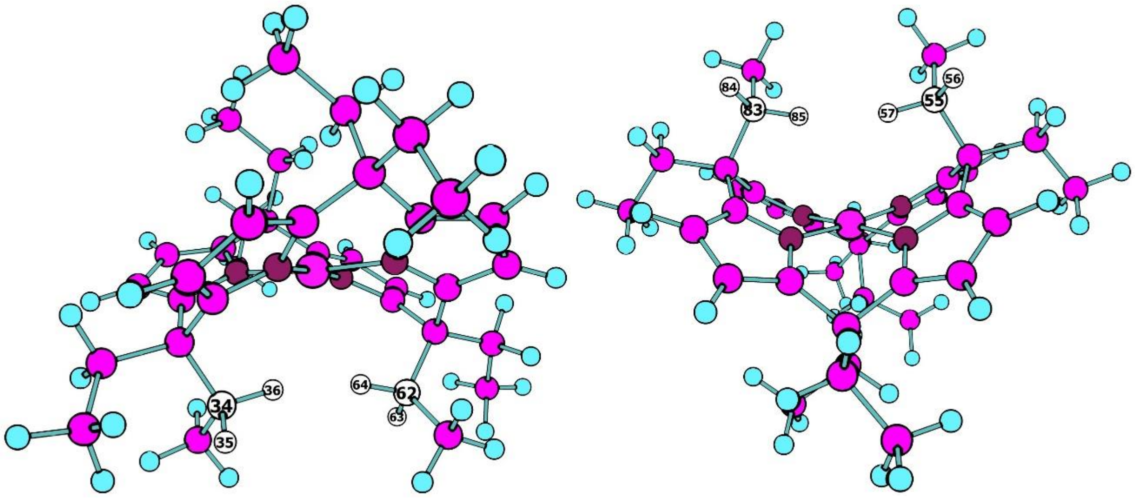

Table S 5: Relevant NBO parameters and metrical data for the gas-phase PBEh-3c optimized structure [**2**$_{PBE-Gas}$] and the SCXRD-HAR derived experimental structure [**2**$_{SCXRD-HAR}$], obtained from the PBE0/def2-TZVPP electron density. For the "inward, agostic" methylene protons: **col1)** number of the considered CX-HY NBO, **col2)** H-Si distance, **col3)** occupation number of the C-H natural bond orbital, **col4)** second-order perturbation energy for the $\sigma_{C-H} \rightarrow Si(p_z)$

Col1	Col2	Col3	Col4
**NBO of (C X)-(H Y)**	**Si-H dist. [pm]**	**σ(C-H) NBO occ. num.**	**2nd ord. pert. [kcal mol^{-1}]**
[**2**$_{PBE-Gas}$]			
C 34- H 35		1.976	
C 34- H 36	263.2	1.953	5.42
C 55- H 56		1.977	
C 55- H 57	283.4	1.962	3.29
C 62- H 63		1.976	
C 62- H 64	263.9	1.954	4.73
C 83- H 84		1.976	
C 83- H 85	282.7	1.961	3.35
[**2**$_{SCXRD-HAR}$]			
C 34- H 35		1.976	
C 34- H 36	273.3	1.960	3.53
C 55- H 56		1.978	
C 55- H 57	258.6	1.950	6.73
C 62- H 63		1.976	
C 62- H 64	240.3	1.935	9.26
C 83- H 84		1.977	
C 83- H 85	296.3	1.967	1.91

As can be seen in the Table S 5, the shorter the Si-H bond distance (**col2**), the stronger is the σ_{C-H} NBO depletion (**col3**), and the more considerable is the second-order perturbation energy (**col4**). The overall magnitude of NBO-derived parameters is reminiscent of those found for α-agostic interactions for titanium or chromium alkyl complexes.[36] A representative $\sigma_{C-H} \rightarrow Si(p_z)$ NBO pair is depicted in Figure S 40.

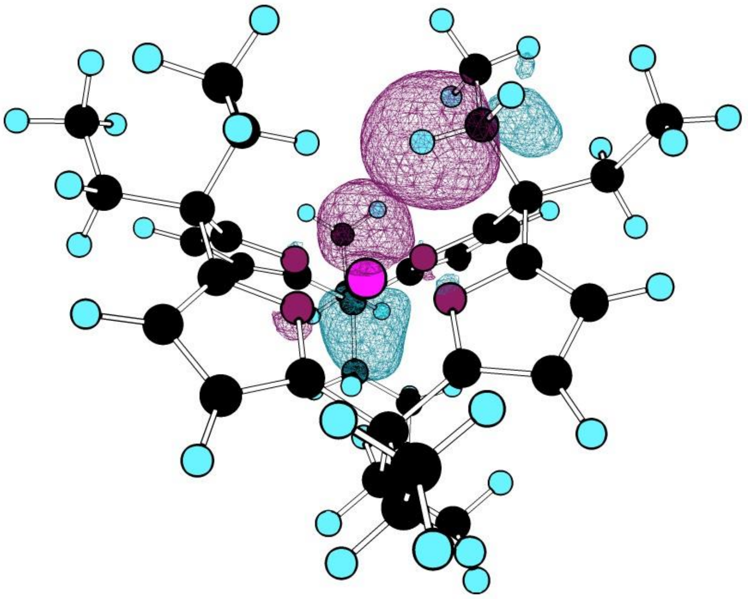

Figure S 40: A representative $\sigma_{C\text{-}H}$ → Si(p_z) NBO pair illustrating the donor-acceptor interaction in the [**2**$_{\mathbf{PBE\text{-}Gas}}$] structure

The Si-H distance (pure metric parameter) is in good correlation with $\sigma_{C\text{-}H}$ NBO occupation number (a parameter derived from the eigenfunctions of the one-particle density matrix) (Figure S 41). This observation is only to explain by a through-space orbital interaction. The (negative) hyperconjugative stabilization factor that occurs in d-block metal β-agostic bonds is absent in this case,[37] but alternative origins for this interaction, such as London dispersion, cannot be ruled out.[38, 39]

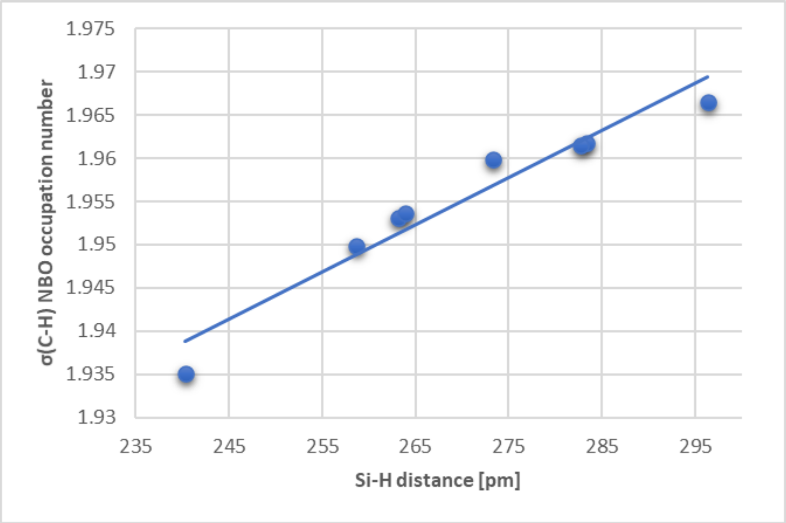

Figure S 41: Correlation of the σC-H NBO occupation number of the "interacting" methylene C-H bonds with the Si-H distance.

Further support for a C-H → Si interaction was provided by a bond critical point in the QTAIM topological analysis of the electron density distribution of the PBE0/def2-TZVPP wavefunction for [**2**$_{\mathbf{SCXRD\text{-}HAR}}$] (default settings in AIMALL). The respective molecular graph can be found in Figure S 42. It occurs between silicon and the closest H64 (see Table S 5). The bond critical point characteristic properties are in line with those reported for d-block metal agostic interactions.[40, 41] For the non-tilted methylene groups in the DFT-gas-phase optimized structure [**2**$_{\mathbf{PBE\text{-}Gas}}$], no bond critical point was found.

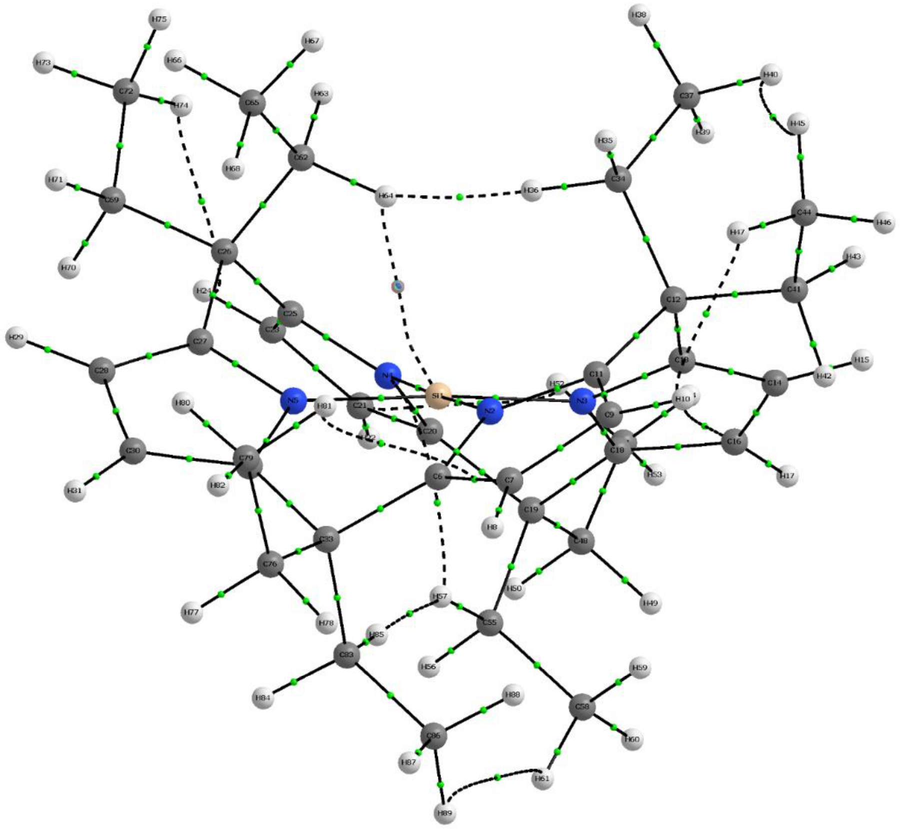

Figure S 42: Molecular graph obtain with AIMAll for the analysis of the PBE0/def2-TZVPP wavefunction of [$\mathbf{2}_{\mathrm{SCXRD\text{-}HAR}}$].

Table S 6: Second order perturbation energy between calix[4]pyrrol nitrogen atoms and silicon-centered acceptor orbitals, as obtained by NBO for the gas-phase PBEh-3c optimized structure [$\mathbf{2}_{\mathrm{PBE\text{-}Gas}}$] from the PBE0/def2-TZVPP electron density. The ligand and the silicon were considered as two individual fragments and the energies are shown for each Si-N bond. The natural electron configuration at silicon is s(0.57)p(1.05).

**Interaction**	N(“sp2”) (in-plane) → Si(3s-orbital)	N(“sp2”) (in-plane) → Si(3p_x-orbital)	N(“sp2”) (in-plane) → Si(3p_y-orbital)	N(“p_z”) (out-plane) → Si(3p_z-orbital)
**Second Order Perturbation Energy [kcal mol^{-1}] N1**	128	66	44	24
**N2**	127	43	68	26
**N3**	131	65	45	28
**N4**	130	41	68	30

Computational evaluation of the binding enthalpy of ethane to octa-protio calix[4]pyrrolato silane

To obtain an estimate of the "unrestricted" interaction energy of a hydrocarbon with a planar calix[4]pyrrolato silane, the model compound octa-protio calix[4]pyrrolato silane **4** and its ethane adduct were optimized at the highly accurate DLPNO-MP2/def2-TZVPP level of theory. The geometry optimization confirmed a planar structure of the free silane **4** and tightly bound ethane in the complex as the global minimum (distance CH-Si = 2.31 Å), and provided thermal correction parameters for the binding enthalpy. The final binding enthalpy was obtained by DLPNO-CCSD(T)/def2-QZVPP level of theory with the matching auxiliary basis sets and VeryTightSCF/tightPNO settings.[24, 42-45] Single-determinant dominated wavefunctions in the DLPNO-CCSD(T) computations were secured by evaluation of the T1 diagnostics (< 0.02).[46]

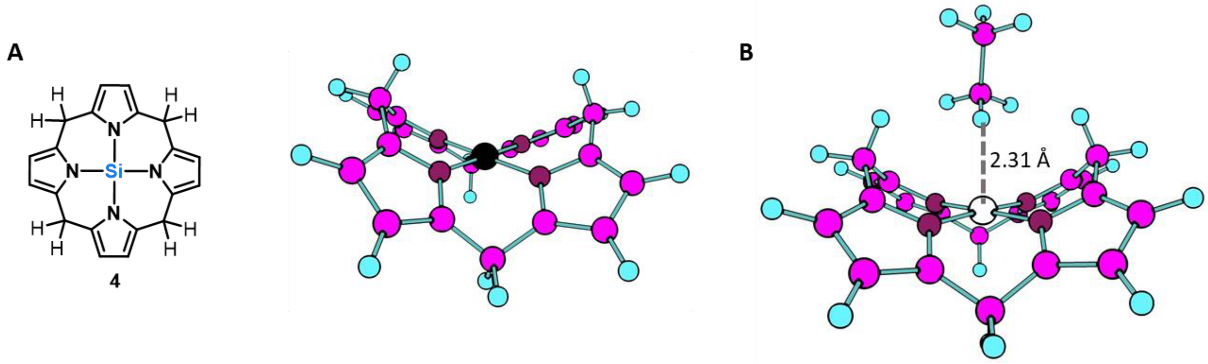

Figure S 43:**A** Lewis Structure and DLPNO-MP2/def2-TZVPP computed structure of model compound **4**. **B** DLPNO-MP2/def2-TZVPP computed structure of the ethane adduct of **4,** including the "agostic" Si-H bond distance.

Table S 7: Computed data for the ethane binding to octa-protio calix[4]pyrrolato silane **4**.

	NIMAG	Enthalpy Correction [kJ mol^{-1}]	Entropy Correction T*S (298K) [kcal/mol^{-1}]	E DLPNO-CCSD(T)/def2-QZVPP [Hartree]	E [kJ mol^{-1}]	Enthalpy H [kJ mol^{-1}]	Gibbs free energy G [kJ mol^{-1}]
**C_2H_6**	0	210.1	16.86	-79.6991	-209249.9	-209039.9	-209110.4
**HCxSi 4**	0	896.2	39.15	-1278.3795	-3356385.5	-3355489.3	-3355653.2
**HCxSi_C_2H_6**	0	1110.6	45.37	-1358.0855	-3565653.5	-3564542.9	-3564732.7
					**ΔE**	**ΔH**	**ΔG**
**C_2H_6-affinity**					**-18.0**	**-13.7**	**30.8**

Time-dependent density functional theory for computation of UV-Vis spectra for 2 and 3

The electronic transitions for **2** and **3** were computed by time-dependent density functional theory at the CAM-B3LYP/def2-TZVPP level of theory.[47] Obtained stick spectra of the first 20 electronic singlet transitions were convoluted with gaussian line-shape functions with a line width of 4000 nm. The computed spectrum and the comparison with the experimental spectrum of **2** and the computed spectrum of **3** can be found in Figure S 44. To account for the neglected solvation effects, all computed values were systematically shifted by +22 nm.

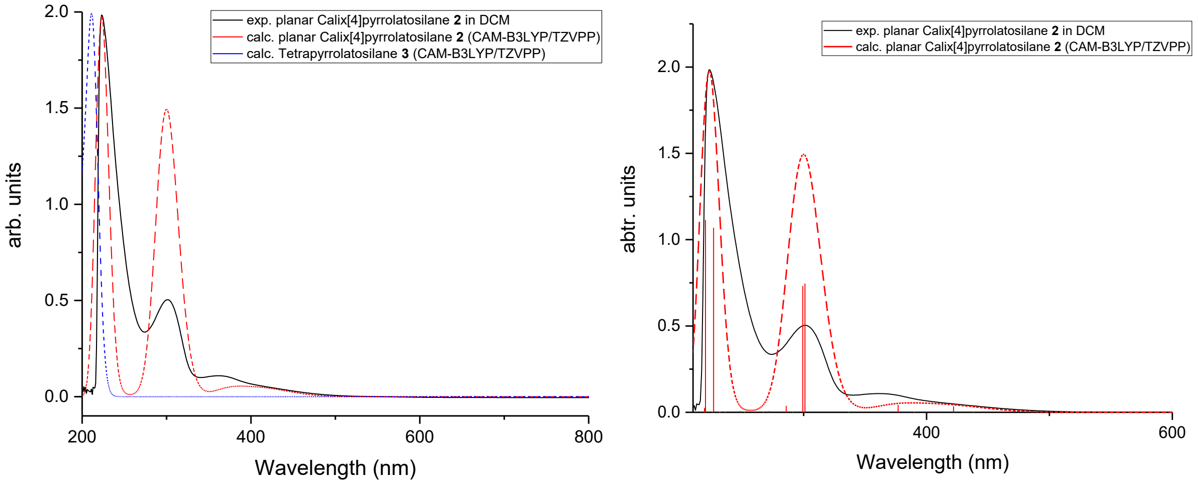

Figure S 44: Comparison of computed (CAM-B3LYP/def2-TZVPP) spectrum of **2** (red) and **3** (blue) and the experimental spectrum of **2** (black) in DCM.

To improve the qualitative description of the low energy electronic transitions, natural transition orbitals (NTO) were calculated.[48] The NTOs contributing to the four lowest energy transitions with significant oscillator strength can be found in Figure S 45. In all cases, the depicted pairs of NTOs cover participation >95%.

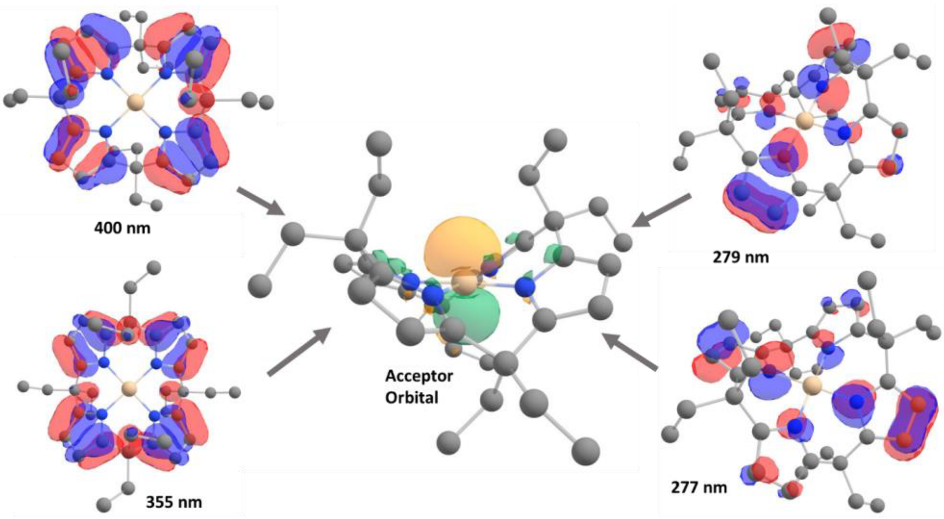

Figure S 45: Isodensity plot (0.06 au) for the involved natural transition orbitals for the electronic excitation at 400, 355, 279 and 277 nm.

As can be derived from the analysis of the NTOs, the lowest energy transitions at 400 and 355 nm occur by charge transfer of the anti-bonding and bonding combination of the local HOMOs of the pyrrole rings into the p_z-type orbital at silicon. The stronger transitions at 277 and 279 nm are of charge transfer of the HOMO-4 orbitals located at the pyrroles, which have a strong contribution of the pyrrole nitrogen electron lone-pairs, into the p_z-type orbital at silicon.

Computation of the three possible reaction pathways of 2 with phenylacetylene

The free energies for the transition states and final products for the two regioisomeric carbosilylation products (Prod_12 and Prod_21) between the model octamethyl-calix[4]pyrrolato silane and phenylacetylene, and the CH-activation product (Prod_CH) were obtained by at the PW6B95-D3(BJ)/def2-QZVPP//PBEh-3c/def2-mSVP level of theory. The corresponding reaction pathways are depicted in Figure S44. In agreement with the spectroscopic results, **Prod_12** is obtained as the kinetic product, whereas the thermodynamic **Prod_21** is not competing due to a high reaction barrier.

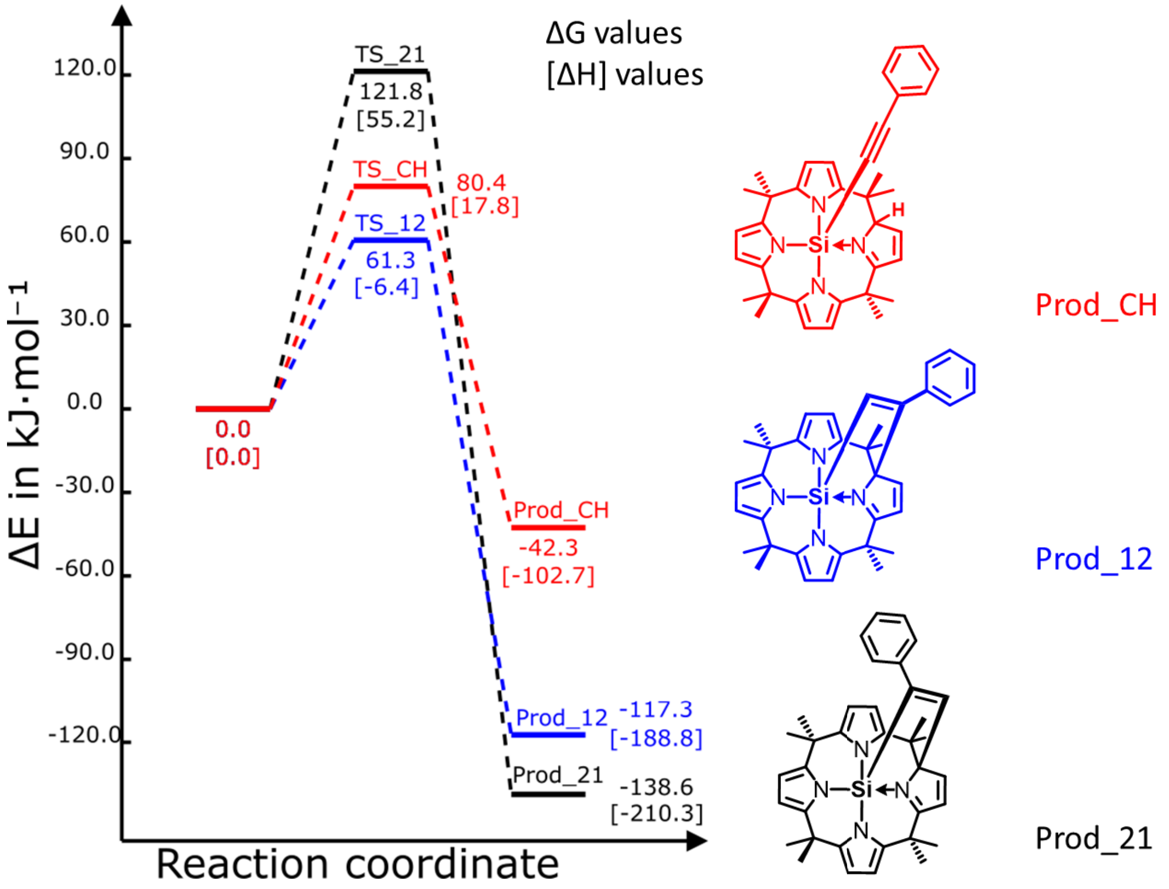

Figure S 46: Reaction pathways of the possible regio-isomeric products for the reaction between octamethyl-calix[4]pyrrolato silane and phenylacetylene. ΔG and [ΔH] values in kJ mol^{-1} obtained at PW6B95-D3(BJ)/def2-QZVPP//PBEh-3c/def2-mSVP.

Supplemental Experimental Procedures

Methods and Materials

Unless otherwise stated, all manipulations were carried out under a dry argon atmosphere using standard Schlenk techniques to prevent oxidation and hydrolysis of sensitive compounds. All solvents were rigorously dried using standard procedures, freshly degassed, and stored over molecular sieve (3 Å resp. 4 Å) before use. All glassware, syringes, magnetic stirring bars, and needles were thoroughly dried. The commercially available chemicals were used as received. All air-sensitive compounds were stored in a glove box (MBraun LABmaster dp, MB-20-G or Sylatech Glovebox) under N_2 atmosphere. The purity and identity of the compounds were confirmed by high-resolution multinuclear NMR spectroscopy, mass spectrometry, and, if possible, X-ray diffraction analysis. ^{1}H, ^{7}Li, $^{13}C\{^{1}H\}$ and ^{29}Si NMR spectra were collected with a Bruker DPX 200, Bruker Advance II 400, or Bruker 2 Advance III 600 NMR spectrometer and referenced to the solvent in use. Chemical shifts are reported as dimensionless δ values in ppm; coupling constants J are given in hertz (Hz). Electrospray ionization mass spectra were obtained with a Bruker ApexQe FT-ICR instrument. MALDI mass spectrometry was performed in a DFTB-Matrix on a Bruker AutoFlex Speed time-of-flight spectrometer. IR-Spectra was recorded on an Agilent Cary 630 spectrometer equipped with a diamond ATR unit processed with MicroLab PC and OriginPro 2017G 64Bit. *meso*-Octaethylcalix[4]pyrrole was prepared from 3-pentanone, pyrrole, and a catalytic amount of CH_3SO_3H in ethanol according to literature and purified via flash column chromatography.[49, 50] Deprotonation of the *meso*-octaethylcalix[4]pyrrole was performed in *n*-hexane, as reported in the literature.[51] Donor free Na[BArfF$_{20}$] was prepared by dissolving 1.00 eq [Ph_3C][B(C_6F_5)] in toluene followed by the addition of 1.00 eq 1M $NaBHEt_3$ toluene solution and stirring for 12 h. The organic phase was decanted, and the colorless residue was washed extensively with *n*-pentane and dried in vacuo. CV measurements were performed with an EmStat3+ Blue from PalmSens Compact Electrochemical Interfaces and processed with PSTrace 5.7 at 298 K. A glassy carbon electrode, with a working area of 0.07 cm^2, was used as a working electrode, a platinic wire as a counter electrode; and a silver wire served as a quasi reference electrode. As internal standard ferrocene was measured at the very end of each measurement. Recrystallized [N^tBu_4][BArF$_{20}$] (0.1 M) was used as electrolyte. Elemental analysis was attempted, but yielded unsatisfying results due to extreme moisture and oxygen sensitivity of the compounds.

Synthesis of [Li(dme)][octa-ethylcalix[4]pyrrolato-chloridosilicate] - [Li(dme)][1]

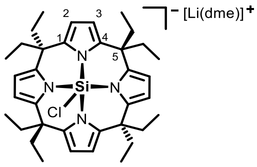

To a solution of 1.00 g *meso*-octaethyl-calix[4]pyrrole (1.84 mmol, 1.00 eq.) in 20 ml *n*-hexane, 4.68 ml 1.6 M *n*-BuLi (7.36 mmol, 4.00 eq.) in hexane was added dropwise at -78 °C and stirred for 20 min. After the solution reached room temperature (rt) it was refluxed for 12 h at 80 °C. Exhaustive deprotonation was verified by ^{1}H NMR spectroscopy in toluene-d$_8$. *n*-Hexane was removed under reduced pressure, and the deprotonated ligand was dissolved in 15 ml dimethoxyethane (DME). 785 mg $SiCl_4$ (4.62 mmol, 2.50 eq.) in 2 ml DME was added dropwise at rt, and the red solution was stirred for 10 min. A conversion to [Li(dme)][**1**] of 40 % was determined by ^{1}H NMR spectroscopy in acetonitrile-d$_3$, beside free *meso*-octaethyl-calix[4]pyrrole and undefined side products. The solvent was removed, and the solid was washed with 20 ml *n*-pentane. Due to the instability of [Li(dme)][**1**] in common solvents except for DME and acetonitrile (ACN), the solid was not further purified but converted directly into the PPh_4 salt. **^{1}H NMR** (ACN-d$_3$, 600 MHz, 295 K) δ[ppm]: 5.75 (d, $^3J_{HH}$ = 3.3 Hz, 4H, H3), 5.70 (d, $^3J_{HH}$ = 3.3 Hz, 4H, H2), 3.46 (s, 4H, $CH_{2\ dme}$), 3.29 (s, 6H,), 2.14 (q, $^3J_{HH}$ = 7.4 Hz, 4H, $CH_{2\ Ethyl}$), 2.01 (q, $^3J_{HH}$ = 7.4 Hz, 4H, $CH_{2\ Ethyl}$), 1.98 (q, 4H, $CH_{2\ Ethyl}$), 1.95 (q, 4H, $CH_{2\ Ethyl}$), 1.13 (t, $^3J_{HH}$ = 7.4 Hz, 6H, $CH_{3\ Ethyl}$), 0.93 (t, $^3J_{HH}$ = 7.4 Hz, 6H, $CH_{3\ Ethyl}$), 0.45 (t, $^3J_{HH}$ = 7.4 Hz, 6H, $CH_{3\ Ethyl}$), 0.26 (t, $^3J_{HH}$ = 7.4 Hz, 6H, $CH_{3\ Ethyl}$). **^{13}C{^{1}H} NMR** (ACN-d$_3$, 150 MHz, 295 K) δ[ppm]: 145.9 (C1/C4), 143.3 (C1/C4), 106.9 (C2), 102.7 (C3), 72.4 ($CH_{2\ dme}$), 58.8 ($CH_{3\ dme}$), 44.7 (C5), 39.1 ($CH_{2\ Ethyl}$), 37.0 (2C, $CH_{2\ Ethyl}$), 26.0 ($CH_{2\ Ethyl}$), 10.8 ($CH_{3\ Ethyl}$), 10.2 ($CH_{3\ Ethyl}$), 9.7 ($CH_{3\ Ethyl}$), 9.3 ($CH_{3\ Ethyl}$). **^{29}Si HMBC NMR** (ACN-d$_3$, 600 MHz, 295 K) δ[ppm]: −129.0.**^{7}Li NMR** (155 MHz, ACN-d$_3$) δ[ppm]: −1.88. **Mass spectrometry** [HR-ESI$^-$]: calc. 599.3336 m/z exp. 599.3377 m/z.

Synthesis of [PPh_4][Ethylcalix[4]pyrrolato-chloridosilicate] - [PPh_4][1]

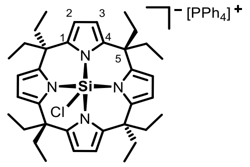

[Li(dme)][**1**] (509 mg, 0.73 mmol, 1.00 eq.) was dissolved in 15 ml acetonitrile and 277 mg PPh_4Cl (0.73 mmol, 1.00 eq.) was added, and the suspension was stirred for 1 h at room temperature. The solvent was removed under reduced pressure, and the solid was dissolved in 5 ml DCM and filtered over a glass frit. The solid was washed until discoloration with small portions of DCM. The solvent was removed from the filtrate, and the residue was washed three times with 50 ml *n*-pentane each. For a better purification, the suspension was put into a sonic bath for 5 min after every washing step. The *n*-pentane was removed by decanting and the solid dried under reduced pressure. The solid was washed three times with 20 ml toluene until no coloration of the toluene washing phase occurred. Last traces of DME were removed by dissolving the solid again in 15 ml DCM and precipitation with 60 ml *n*-pentane. The solvent was decanted, and the residual volatiles were removed in vacuo. Compound [PPh_4][**1**] (0.628 g, 0.67 mmol) was obtained as a colorless solid in 91 % yield. Suitable crystals for SCXRD analysis were obtained from a saturated benzene solution at room temperature. **^{1}H NMR** (DCM-d_2, 400 MHz, 295 K) δ[ppm]: 7.86 (m, 4H, PPh_4^+), 7.67 (m, 8H, PPh_4^+), 7.57 (m, 8H, PPh_4^+), 5.75 (d, $^3J_{HH}$ = 3.2 Hz, 4H, H3), 5.67 (d, $^3J_{HH}$ = 3.2 Hz, 4H, H2), 2.10 (q, $^3J_{HH}$ = 7.4 Hz, 4H, $CH_{2\ Ethyl}$), 2.01 (q, $^3J_{HH}$ = 7.4 Hz, 4H, $CH_{2\ Ethyl}$), 1.99 (q, $^3J_{HH}$ = 7.4 Hz, 4H, $CH_{2\ Ethyl}$), 1.88 (q, $^3J_{HH}$ = 7.4 Hz, 4H, $CH_{2\ Ethyl}$), 1.12 (t, $^3J_{HH}$ = 7.4 Hz, 6H, $CH_{3\ Ethyl}$), 0.91 (t, $^3J_{HH}$ = 7.4 Hz, 6H, $CH_{3\ Ethyl}$), 0.47 (t, $^3J_{HH}$ = 7.4 Hz, 6H, $CH_{3\ Ethyl}$), 0.22 (t, $^3J_{HH}$ = 7.4 Hz, 6H, $CH_{3\ Ethyl}$). **^{13}C{^{1}H} NMR** (DCM-d_2, 100 MHz, 295 K) δ[ppm]: 145.8 (C1/C4), 143.0 (C1/C4), 136.1 (d, $^4J_{CP}$ = 3.1 Hz, CH, PPh_4^+), 134.8 (d, $^2J_{CP}$ = 10.3 Hz, CH, PPh_4^+), 131.0 (d, $^3J_{CP}$ = 12.9 Hz, CH, PPh_4^+), 117.9 (d, $^1J_{CP}$ = 89.8 Hz, C_q, PPh_4^+), 106.0 (C2), 101.9 (C3), 44.3 (C5), 40.6 ($CH_{2\ Ethyl}$), 39.3 ($CH_{2\ Ethyl}$), 36.4 ($CH_{2\ Ethyl}$), 25.4 ($CH_{2\ Ethyl}$), 10.5 ($CH_{3\ Ethyl}$), 10.0 ($CH_{3\ Ethyl}$), 9.3 ($CH_{3\ Ethyl}$), 9.0 ($CH_{3\ Ethyl}$). **^{29}Si HMBC NMR** (DCM-d_2, 400 MHz, 295 K) δ[ppm]: −129.0. **^{31}P NMR** (DCM-d_2, 161 MHz, 295 K) δ[ppm]: 23.22. **Mass spectrometry** [HR-ESI$^-$]: calc. 599.3336 m/z exp. 599.3377 m/z. **IR spectroscopy** ṽ[cm^{-1}]: 3099 (w) , 2958 (m), 2925 (m), 2868 (m), 1507 (w), 1454 (m), 1413 (m), 1374 (m), 1326 (w), 1247 (m), 1076 (s), 1009 (w), 970 (m), 857 (m), 765 (s), 706 (w).

Synthesis of [Ethylcalix[4]pyrrolato-silane] – 2

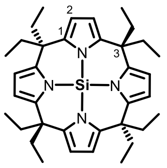

Freshly prepared donor free sodium tetrakis(pentafluorophenyl)borate (68.4 mg, 28.5 µmmol, 1.10 eq.) was added to a solution of [PPh_4][**1**] (66.0 mg, 70.2 µmmol, 1.00 eq.) in 4 ml DCM at room temperature. The solution was stirred for 12 h while turning yellow, and the solvent was removed under reduced pressure. 10 ml *n*-hexane were added, and the solution was stirred for 2 h and filtered off. The solvent of the filtrate was removed, and the residue was dissolved in 10 ml *n*-hexane, stirred for 24 h and filtered. The clear yellow solution was stirred for another 24 h to drive the elimination of NaCl until completeness. The yellow solution was filtered again, and the solvent was removed. After drying in vacuo, the neutral silane **2** was obtained as a yellow solid 78 % yield (30.9 mg, 54.7 µmmol). Orange crystals of **2** were obtained by storing a concentrated solution of **2** in DCM for 7 days at -40 °C. At room temperature, slow decomposition in DCM-d_2 and C_6D_6 was observed over 4 days. Remark: The first step in this reaction is the cation exchange from PPh_4^+ to Na^+. The SCXRD of this intermediate $[Na]_4$[**1**]$_4$ was obtained by storing a concentrated solution of [PPh_4][**1**] and $NaBArF_{20}$ in DCM for 5 days at room temperature without further workup. **^{1}H NMR** (DCM-d_2, 400 MHz, 295 K) δ[ppm]: 5.89 (s, 8H, H2), 2.04 (q, $^3J_{HH}$ = 7.4 Hz, 8H, $CH_{2\ Ethyl}$), 1.93 (q, $^3J_{HH}$ = 7.4 Hz, 8H, $CH_{2\ Ethyl}$), 1.10 (t, $^3J_{HH}$ = 7.4 Hz, 12H, $CH_{3\ Ethyl}$), 0.66 (t, $^3J_{HH}$ = 7.4 Hz, 12H, $CH_{3\ Ethyl}$). **^{13}C{^{1}H} NMR** (DCM-d_2, 100 MHz, 295 K) δ[ppm]: 143.9 (C1), 106.5 (C2), 45.9 ($CH_{2\ Ethyl}$), 42.4 (C3), 24.8 ($CH_{2\ Ethyl}$), 9.4 ($CH_{3\ Ethyl}$), 9.3 ($CH_{3\ Ethyl}$). **^{29}Si HMBC NMR** (DCM-d_2, 400 MHz, 295 K): −55.6 ppm. **Mass spectrometry**: [ESI$^-$]: $C_{36}H_{48}N_4Si{*}OH^-$: calc. 581.3675 m/z exp. 581.3761 m/z. [MALDI, DFTB-Matrix] [m/z]: $C_{36}H_{48}N_4Si{*}H_3O^+$: calc. 583.383 m/z exp. 583.397 m/z. **IR**

spectroscopy ṽ[cm^{-1}]: 3112 (w), 2963 (s), 2931 (s), 2872 (s), 2815 (w), 1344 (w), 1514 (m), 1455 (s), 1378 (m), 1364 (m), 1322 (w), 1297 (w), 1278 (w), 1234 (s), 1132 (s), 1087 (s), 973 (s), 953 (m), 926 (w), 857 (w), 736 (s), 713 (m), 681 (w).

Synthesis of [Ethylcalix[4]pyrrolato-silane-phenylacetylene] activation product

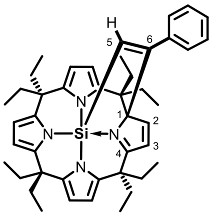

To a solution of **2** (15.0 mg, 26.5 µmol) in 0.5 ml DCM-d_2, phenylacetylene (0.1 mL) was added at room temperature. Quantitative conversion was observed by ^{1}H NMR spectroscopy. Isolation of the activation product was performed as follows: To a solution of [PPh_4][**1**] (70.0 mg, 74.5 µmol, 1.00 eq.) in 5 ml dichloromethane Na[$BArF_{20}$] (57.5 mg, 81.9 µmol, 1.10 eq.) and phenylacetylene (10.0 mg, 96.8 µmol, 1.30 eq.) were added and stirred for 16 h. All volatiles were removed *in vacuo* and the solid was stirred for 30 min in 5 ml *n*-hexane. The yellow solution was filtered and the solvent was removed under reduced pressure. This step was performed twice. The product was dried for 30 min *in vacuo*. The yellow phenylacetylene activation product (35.4 mg, 52.5 µmol) was obtained in 70 % yield. **^{1}H NMR** (DCM-d_2, 600 MHz, 295 K) δ[ppm]: 8.04 (d, $^3J_{HH}$ = 5.4 Hz, 1H, H2), 7.26 (m, 3H, H_{phenyl}), 7.19 (m, 2H, $^3J_{HH}$ = 5.4 Hz, 1H, H3), 6.07 (s, 1H, H_{alkyne}), 6.05 (d, $^3J_{HH}$ = 3.2 Hz, 1H, $H_{pyrrole}$), 6.00 (d, $^3J_{HH}$ = 3.2 Hz, 1H, $H_{pyrrole}$), 5.96 (m, 2H, $H_{pyrrole}$), 5.86 (d, $^3J_{HH}$ = 3.4 Hz, 1H, $H_{pyrrole}$), 5.78 (d, $^3J_{HH}$ = 3.4 Hz, 1H, $H_{pyrrole}$), 2.25 (m, 2H, H_{CH2}), 2.18 (m, 2H, H_{CH2}), 2.07 (m, 2H, H_{CH2}), 1.92 (m, 4H, H_{CH2}), 1.81 (m, 2H, H_{CH2}), 1.73 (m, 2H, H_{CH2}), 1.28 (m, 2H, H_{CH2}), 1.18 (t, $^3J_{HH}$ = 7.3 Hz, 3H, H_{CH3}), 1.15 (t, $^3J_{HH}$ = 7.3 Hz, 3H, H_{CH3}), 0.78 (t, $^3J_{HH}$ = 7.3 Hz, 3H, H_{CH3}), 0.73 (t, $^3J_{HH}$ = 7.3 Hz, 3H, H_{CH3}), 0.65 (t, $^3J_{HH}$ = 7.3 Hz, 3H, H_{CH3}), 0.61 (t, $^3J_{HH}$ = 7.3 Hz, 3H, H_{CH3}), 0.39 (t, $^3J_{HH}$ = 7.3 Hz, 3H, H_{CH3}), 0.35 (t, $^3J_{HH}$ = 7.3 Hz, 3H, H_{CH3}). **^{13}C{^{1}H} NMR** (DCM-d_2, 150 MHz, 295 K) δ[ppm]: 184.1 (C4), 158.4 (C2), 152.9 (C_q), 149.6 (C_q), 145.2 (C_q), 143.5 (C5), 143.0 (C_q), 142.8 (C_q), 141.1 (C6), 140.1 (C_q), 136.4 (C_q), 128.8 (2C, CH_{phenyl}), 128.5 (2C, CH_{phenyl}), 127.9 (CH_{phenyl}), 127.0 (C3), 111.1 (CH), 109.5 (CH), 108.7 (CH), 105.6 (CH), 105.2 (CH), 105.0 (CH), 95.1 (C1), 55.2 (C_q), 46.0 (C_q), 44.8 (C_q), 42.8 (C_q), 40.7 (CH_2), 37.1 (CH_2), 34.6 (CH_2), 31.3 (CH_2), 29.3 (CH_2), 26.2 (CH_2), 25.8 (CH_2), 23.3 (CH_2), 9.9 (CH_3), 9.8 (CH_3), 9.7 (CH_3), 9.6 (CH_3), 9.4 (CH_3), 9.3 (CH_3), 9.0 (CH_3), 8.0 (CH_3). **^{29}Si HMBC NMR** (DCM-d_2, 600 MHz, 295 K) δ[ppm]: −126.0 ppm. **Mass spectrometry**: [ESI^+]: $C_{44}H_{54}N_4Si{*}H^+$ calc. 667.4195 m/z exp. 667.4141 m/z.

Reduction of 2 with $CoCp^*_2$ in dichloromethane

To a solution of **2** (10.0 mg, 17.7 µmol, 1.00 eq) in DCM-d_2 at room temperature, $CoCp_2^*$ was added stepwise (0.1, 0.2, 0.6 and 1.0 eqivalents), and the solution turned from yellow to light yellow, indicating the formation of chlorido silicate [$CoCp_2$*][**1**] as the major product (Figure S 15). Addition of an excess of $CoCp_2^*$ in one portion lead to the clean formation of [**1**]$^-$ without side products (Figure S 16). The identity of the chlorido silicate [**1**]$^-$ was verified by ^{1}H and ^{29}Si HMBC NMR spectroscopy, ESI-MS and by comparison with the other salts of [**1**]$^-$. Due to the rapid reaction, no radical intermediate could be detected by EPR-spectroscopy. The fate of the chloride atom donor was not inspected.

Supplemental References

1. Otwinowski, Z., and Minor, W. (1997). Processing of X-ray diffraction data collected in oscillation mode. Methods Enzymol. *276*, 307-326.
2. SAINT (2016) (Bruker AXS GmbH, Karlsruhe, Germany).
3. Sheldrick, G.M. (2004-2014) (SADABS, Bruker AXS GmbH, Karlsruhe, Germany).
4. Krause, L., Herbst-Irmer, R., Sheldrick, G.M., and Stalke, D. (2015). Comparison of silver and molybdenum microfocus X-ray sources for single-crystal structure determination. J. Appl. Crystallogr. *48*, 3-10.
5. Sheldrick, G.M. (2014-2018) (SHELXT, Program for Crystal Structure Solution, University of Göttingen, Germany).
6. Sheldrick, G.M. (2015). SHELXT - Integrated space-group and crystal-structure determination. Acta Crystallogr. A, 3-8.
7. Sheldrick, G.M. (2012-2018) (SHELXL-20xx, University of Göttingen and Bruker AXS GmbH, Karlsruhe, Germany).
8. Robinson, W., and Sheldrick, G.M. (1988). Crystallographic Computing 4, N.W. Isaaks, and M.R. Taylor, eds. (Ch. 22, IUCr and Oxford University Press, Oxford, UK).
9. Sheldrick, G.M. (2008). A short history ofSHELX. Acta Crystallogr. A *64*, 112-122.
10. Sheldrick, G.M. (2015). Crystal structure refinement withSHELXL. Acta Crystallogr. C *71*, 3-8.
11. Dolomanov, O.V., Bourhis, L.J., Gildea, R.J., Howard, J.A.K., and Puschmann, H. (2009). OLEX2: a complete structure solution, refinement and analysis program. J. Appl. Crystallogr. *42*, 339-341.
12. Capelli, S.C., Burgi, H.-B., Dittrich, B., Grabowsky, S., and Jayatilaka, D. (2014). Hirshfeld atom refinement. IUCrJ *1*, 361-379.
13. Woińska, M., Grabowsky, S., Dominiak, P.M., Woźniak, K., and Jayatilaka, D. (2016). Hydrogen atoms can be located accurately and precisely by x-ray crystallography. Sci. Adv. *2*, e1600192.
14. Kleemiss, F., Puschmann, H., Dolomanov, O., Bodensteiner, M., Peyerimhoff, N., Midgley, L., Bourhis, L., Genoni, A., Malaspina, L.A., Jayatilaka, D., *et al.* (2020). Generalizing Non-Spherical Structure Refinement: Hirshfeld Atom Refinement in NoSpherA2. Chem. Sci., DOI: 10.1039/D1030SC05526C.
15. Neese, F. (2012). The ORCA program system. WIREs Comput. Mol. Sci. *2*, 73-78.
16. Andrienko, G.A. (https://www.chemcraftprog.com).
17. Grimme, S., Brandenburg, J.G., Bannwarth, C., and Hansen, A. (2015). Consistent structures and interactions by density functional theory with small atomic orbital basis sets. J. Chem. Phys. *143*, 054107.
18. Kruse, H., and Grimme, S. (2012). A geometrical correction for the inter- and intra-molecular basis set superposition error in Hartree-Fock and density functional theory calculations for large systems. J. Chem. Phys. *136*, 154101.
19. Grimme, S., Antony, J., Ehrlich, S., and Krieg, H. (2010). A consistent and accurate ab initio parametrization of density functional dispersion correction (DFT-D) for the 94 elements H-Pu. J. Chem. Phys. *132*, 154104.
20. Grimme, S., Ehrlich, S., and Goerigk, L. (2011). Effect of the damping function in dispersion corrected density functional theory. J. Comput. Chem. *32*, 1456-1465.
21. Sure, R., Brandenburg, J.G., and Grimme, S. (2016). Small Atomic Orbital Basis Set First-Principles Quantum Chemical Methods for Large Molecular and Periodic Systems: A Critical Analysis of Error Sources. ChemistryOpen *5*, 94-109.
22. Frenzel, A., Herbst-Irmer, R., Klingebiel, U., Noltemeyer, M., and Schäfer, M. (1995). Indolyl- und Pyrrolylsilane – Synthese und Kristallstruktur / Indolyl- and Pyrrolylsilanes – Syntheses and Crystal Structures. Z. Naturforsch. B *50*, 1658.
23. Zhao, Y., and Truhlar, D.G. (2005). Design of Density Functionals That Are Broadly Accurate for Thermochemistry, Thermochemical Kinetics, and Nonbonded Interactions. J. Phys. Chem. A *109*, 5656-5667.
24. Weigend, F., and Ahlrichs, R. (2005). Balanced basis sets of split valence, triple zeta valence and quadruple zeta valence quality for H to Rn: Design and assessment of accuracy. Phys. Chem. Chem. Phys. *7*, 3297-3305.
25. Stoychev, G.L., Auer, A.A., and Neese, F. (2017). Automatic Generation of Auxiliary Basis Sets. J. Chem. Theory Comput. *13*, 554-562.
26. Neese, F., Wennmohs, F., Hansen, A., and Becker, U. (2009). Efficient, approximate and parallel Hartree–Fock and hybrid DFT calculations. A ‘chain-of-spheres’ algorithm for the Hartree–Fock exchange. Chem. Phys. *356*, 98-109.

27. Neese, F. (2003). An improvement of the resolution of the identity approximation for the formation of the Coulomb matrix. J. Comput. Chem. *24*, 1740-1747.
28. Reed, A.E., Curtiss, L.A., and Weinhold, F. (1988). Intermolecular interactions from a natural bond orbital, donor-acceptor viewpoint. Chem. Rev. *88*, 899-926.
29. Glendening, E.D., Landis, C.R., and Weinhold, F. (2012). Natural bond orbital methods. Wiley Interdiscip. Rev. Comput. Mol. Sci *2*, 1-42.
30. Bader, R.F.W. (1991). A quantum theory of molecular structure and its applications. Chem. Rev. *91*, 893-928.
31. Adamo, C., and Barone, V. (1999). Toward reliable density functional methods without adjustable parameters: The PBE0 model. J. Chem. Phys. *110*, 6158-6170.
32. Ernzerhof, M., and Scuseria, G.E. (1999). Assessment of the Perdew–Burke–Ernzerhof exchange-correlation functional. J. Chem. Phys. *110*, 5029-5036.
33. Medvedev, M.G., Bushmarinov, I.S., Sun, J., Perdew, J.P., and Lyssenko, K.A. (2017). Density functional theory is straying from the path toward the exact functional. Science *355*, 49-52.
34. NBO 7.0. E. D. Glendening, J. K. Badenhoop, A. E. Reed, J. E. Carpenter, J. A. Bohmann, C. M. Morales, P. Karafiloglou, C. R. Landis, and F. Weinhold, Theoretical Chemistry Institute, University of Wisconsin, Madison, WI (2018)
35. AIMAll (Version 19.10.12), Todd A. Keith, TK Gristmill Software, Overland Park KS, USA, 2019 (aim.tkgristmill.com)
36. Thakur, T.S., and Desiraju, G.R. (2007). Theoretical investigation of C–H···M interactions in organometallic complexes: A natural bond orbital (NBO) study. J. Mol. Struc.-Theochem *810,* 143-154.
37. Scherer, W., and McGrady, G.S. (2004). Agostic Interactions in d0 Metal Alkyl Complexes. Angew. Chem. Int. Edit. *43,* 1782-1806.
38. Lu, Q., Neese, F., and Bistoni, G. (2018). Formation of Agostic Structures Driven by London Dispersion. Angew. Chem. Int. Edit. *57,* 4760-4764.
39. Lin, X., Wu, W., and Mo, Y. (2020). A theoretical perspective of the agostic effect in early transition metal compounds. Coordin. Chem. Rev. *419,* 213401.
40. Lein, M. (2009). Characterization of agostic interactions in theory and computation. Coordin. Chem. Rev. *253,* 625-634.
41. Tognetti, V., Joubert, L., Raucoules, R., De Bruin, T., and Adamo, C. (2012). Characterizing Agosticity Using the Quantum Theory of Atoms in Molecules: Bond Critical Points and Their Local Properties. J. Phys. Chem. A *116,* 5472-5479.
42. Neese, F., Hansen, A., and Liakos, D.G. (2009). Efficient and accurate approximations to the local coupled cluster singles doubles method using a truncated pair natural orbital basis. J Chem Phys *131.*
43. Riplinger, C., and Neese, F. (2013). An efficient and near linear scaling pair natural orbital based local coupled cluster method. J Chem Phys *138.*
44. Riplinger, C., Sandhoefer, B., Hansen, A., and Neese, F. (2013). Natural triple excitations in local coupled cluster calculations with pair natural orbitals. J Chem Phys *139.*
45. Weigend, F., Furche, F., and Ahlrichs, R. (2003). Gaussian basis sets of quadruple zeta valence quality for atoms H–Kr. J. Chem. Phys. *119,* 12753-12762.
46. Lee, T.J., and Taylor, P.R. (1989). A diagnostic for determining the quality of single-reference electron correlation methods. Int. J. Quantum Chem. *36,* 199-207.
47. Yanai, T., Tew, D.P., and Handy, N.C. (2004). A new hybrid exchange–correlation functional using the Coulomb-attenuating method (CAM-B3LYP). Chem. Phys. Lett. *393,* 51-57.
48. Martin, R.L. (2003). Natural transition orbitals. J. Chem. Phys. *118,* 4775-4777.
49. Jacoby, D., Floriani, C., Chiesi-Villa, A., and Rizzoli, C. (1991). The π and σ bonding modes of meso-octaethylporphyrinogen to transition metals: the X-ray structure of a meso-octaethylporphyrinogen–zirconiuml(IV) complex and of the parent meso-octaethylporphyrinogen ligand. J. Chem. Soc. Chem. Comm., 790-792.
50. Depraetere, S., Smet, M., and Dehaen, W. (1999). N-confused calix[4]pyrroles. Angew Chem Int Edit *38,* 3359-3361.
51. De Angelis, S., Solari, E., Floriani, C., Chiesi-Villa, A., and Rizzoli, C. (1994). Dalton communications. Solvent-dependent forms of lithiated 5,5,10,10,15,15,20,20-octaethylporphyrinogen in solution and in the solid state and reaction with tetrahydrofuran. J. Chem. Soc. Dalton, 2467-2469.
