# Supplementary material for: An isolable, crystalline complex of square-planar silicon(IV)
Source: Chem. 2021 Aug 12;7(8):2151–9. doi: 10.1016/j.chempr.2021.05.002 (PMC8367297; doi:10.1016/j.chempr.2021.05.002)
Supplement: Document S2. Article plus supplemental information [file mmc2.pdf]

Chem

CellPress
OPEN ACCESS

Article

An isolable, crystalline complex of square-planar silicon(IV)

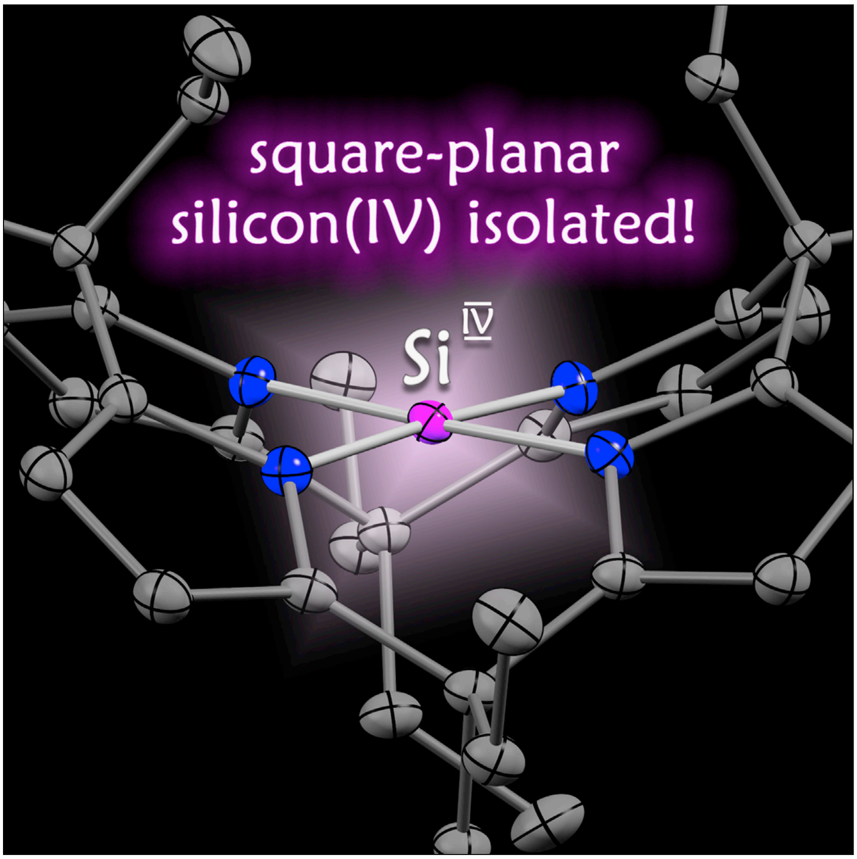

Fabian Ebner, Lutz Greb

greb@uni-heidelberg.de

Highlights

The first square-planar silicon(IV) is isolated

A low-lying LUMO provokes CH-agostic interaction and visible-light absorption

Transition metals are mimicked without leaving the natural oxidation state

Our Earth's crust is covered with compounds of silicon(IV). In each, tetracoordinated silicon(IV) arranges its four substituents in a tetrahedral fashion, while other environments are unknown. This work describes the isolation and properties of a molecular complex with square-planar silicon(IV). The flattened structural motif provokes a range of features that primes the second most abundant element for new applications in catalysis and materials science.

Ebner & Greb, Chem 7, 2151–2159
August 12, 2021 © 2021 The Authors. Published by Elsevier Inc.
https://doi.org/10.1016/j.chempr.2021.05.002

Chem

CellPress OPEN ACCESS

Article

An isolable, crystalline complex of square-planar silicon(IV)

Fabian Ebner[1] and Lutz Greb[1,2,*]

SUMMARY

The structure and reactivity of silicon(IV), the second most abundant element in our Earth's crust, is determined by its invariant tetrahedral coordination geometry. Silicon(IV) with a square-planar configuration (*ptSi*^{IV}) represents a transition state. Quantum theory supported the feasibility of stabilizing *ptSi*^{IV} by structural constraint, but its isolation has not been achieved yet. Here, we present the synthesis and full characterization of the first square-planar coordinated silicon(IV). The planarity provokes an extremely low-lying unoccupied molecular orbital that induces unusual silicon redox chemistry and CH-agostic interactions. The small separation of the frontier molecular orbitals enables visible-light ligand-element charge transfer and bond-activation reactivity. Previously, such characteristics have been reserved for d-block metals or low-valent p-block elements. Planarization transfers them, for the first time, to a p-block element in the normal valence state.

The bigger picture

Tetrahedral silicon(IV) compounds are the building blocks of our Earth's crust. Here, we describe the first species of silicon(IV) with a square-planar configuration. The structural deformation has substantial consequences for the compounds' physicochemical properties and imparts features usually associated with transition metals. Upon planarization, the frontier molecular orbital gap shrinks by more than 50% and enables ligand-element charge transfer, CH-bond agostic interactions, and spontaneous reactivity with inert bonds. Small frontier molecular orbital gaps are critical for bond-activation reactivity, catalysis, and photochemistry with transition metals. Traditional approaches to mimic these characteristics with the more abundant p-block elements rely on unusual valence or oxidation states. With the realization of square-planar silicon(IV), these peculiarities start reaching p-block elements in their natural oxidation states.

INTRODUCTION

Silicon is the second most abundant element in the Earth's crust, constituting 28 wt % as tetrahedral silicon(IV).[1] During past decades, groundbreaking low-valent states of silicon, such as silylium ions,[2,3] silylenes,[4–6] silylium ylidenes,[7–10] disilenes,[11] disilynes,[12] trisilaallene,[13] or silylones,[14,15] have been isolated (Figure 1A).[16] The modified electronic structure in those compounds compared with normal-valent silicon(IV) evolved into a linchpin for unique reactivities and catalytic applications.[16–21] Naturally, these achievements channeled the primary interest of modern silicon chemistry on low-valent states while pushing conventional silicon(IV) into a niche of being precursor with classical behavior only. However, it is not only the valence or oxidation state that changes the reactivity and properties of an element, but likewise the type and the spatial arrangement of its ligands.[22]

Structural constraint of group 13 and 15 p-block elements for reactivity enhancement is burgeoning in recent years[23–30] but remain less explored for group 14. For silicon *in unnatural oxidation states*, planar-tetracoordinated states (*ptSi*) have been observed in the gas phase (I, Figure 1B),[31] in polysilanes (II),[32,33] or in trapezoid complexes with transition metals (III).[34] However, the isolation of planar-tetracoordinated silicon in the *intrinsically more stable, natural oxidation state IV* (*ptSi*^{IV}) has not been achieved.[35–38] Indeed, the square-planar configuration of silicon(IV) constitutes a high-energy transition state during silicon inversion.[39] Judged by the computed electronic structure of *ptSi*^{IV}, fundamental changes can be expected upon planarization.[39,40] The *ptSi*^{IV} has a silicon-centered p_z-type lowest unoccupied molecular orbital (LUMO) of much lower energy than the tetrahedral state and a ligand-centered highest occupied molecular orbital (HOMO) raised in energy (Figure 1C).[39,41] However, further computational studies supported the feasibility of

This is an open access article under the CC BY license (http://creativecommons.org/licenses/by/4.0/).

stabilizing *ptSi*^{IV} into a ground state by appropriate substituents.[42,43] If incorporated into extended 2D-structures, unique electronic properties were attributed to *in silico*-designed *ptSi*^{IV}-materials that await experimental realization.[41,44–46] More recent computational studies on molecular complexes of *ptSi*^{IV} even proposed carbenoid character to the square-planar silicon(IV).[47] Ultimately, it is the small HOMO-LUMO gap that renders d-block metals or p-block elements in the low-valence states as suitable for bond activation.[48] Hence, planarization might project those beneficial features to silicon—while staying in the normal oxidation state. This assumption is verified in this work using the macrocyclic calix[4]pyrrolato ligand.[49,50]

RESULTS AND DISCUSSION

Synthesis and characterization of the planar-tetracoordinated silicon complex

The reaction of the tetra lithium salt of *meso*-octaethyl-calix[4]pyrrole with $SiCl_4$ in dimethoxyethane and subsequent salt metathesis with PPh_4Cl provided the *meso*-octaethyl-calix[4]pyrrolato chlorido silicate [PPh_4][**1**] in an overall isolated yield of 36% up to 600 mg (Figure 2A). A ^{29}Si-NMR chemical shift of —129 ppm in CD_2Cl_2 and single-crystal X-ray diffraction analysis (SCXRD, Figure S24) verified the structure of the first anionic SiN_4Cl motif, showing structural parameters similar to the hydridosilicate reported previously.[40] The reaction of [PPh_4][**1**] with sodium tetrakis(pentafluorophenyl)borate in CH_2Cl_2 afforded a macrocyclic, tetrameric sodium salt of the chlorido silicate [Na]$_4$[**1**]$_4$ by salt metathesis (see Figure S25 for SCXRD). Stirring a solution of [Na]$_4$[**1**]$_4$ in *n*-hexane induced the transformation into a new, clean reaction product **2**, isolated in 78% yield. The ^{29}Si-NMR chemical shift of **2** (−55.6 ppm) indicates a tetracoordinated silicon center. The occurrence of only one peak for the pyrrole ring protons in the ^{1}H NMR spectrum, and a 2-fold set of signals for the ethyl group, illustrates a highly symmetric species in line with effective D_{2d} symmetry on the NMR time scale. Upon storing a solution of **2** in CH_2Cl_2 for 7 days at −40°C, block-shaped orange crystals developed (Figure 2B). SCXRD analysis revealed a tetracoordinated silicon with typical Si–N bond lengths (178.7(1) to 179.7(1) pm) and an almost ideal square-planar coordination geometry (N1–Si–N3 = 178.2(1)°, N2–Si–N4 = 176.1(1)°, N1–Si–N2 = 90.2°, Figures 2C and 2D).

The location of all protons in the difference Fourier map and structure solution by Hirshfeld atom refinement[51] (R_1 = 2.03; wR_2 = 4.36) disclosed another structural peculiarity: two of the four inward-oriented methylene groups tilt toward the silicon center, reflected by varying CH····Si distances, e.g., Si–H21b (273.3(8) pm) versus Si–H29b (240.3(8) pm) (Figure 2D). In the solid-state crystal packing, neither intermolecular contacts can be made responsible for this distortion nor does it occur in the isoelectronic aluminate.[52] Hence, an attractive intramolecular CH····Si interaction was suspected as the cause. ^{1}H coupled ^{13}C-NMR spectroscopy shows a coupling constant for the inward-oriented methylene groups ($^1J_{C–H}$ = 123 Hz, Figure S13) that is effectively reduced by 32 Hz (averaged over the 8 protons, absolute reduction 4 Hz) compared with the outward CH_2-groups or the protonated, free ligand ($^1J_{C–H}$ = 127 Hz, Figure S14). A further spectroscopic indication for an interaction is provided by FTIR-vibrational spectroscopy, revealing a shoulder in the C–H vibrational band for **2**, red-shifted by 57 cm^{-1}, which is absent in the protonated, free ligand or the chlorido silicate **1**$^-$ (Figure S32). Such observations, although weak in this case, are characteristic for C–H agostic interactions in transition metal complexes[53,54] but unusual as the source of structural deformation in p-block element chemistry. E–H σ-bond donor-acceptor interactions in the p-block were only observed for combinations of more hydridic bonds with the most Lewis acidic boranes,[55,56] silylium ions,[2,57] anagostic,[9] or structurally enforced.[58]

[1]Anorganisch-Chemisches Institut, Ruprecht-Karls-Universität Heidelberg, Im Neuenheimer Feld 275, 69120 Heidelberg, Germany

[2]Lead contact

*Correspondence: greb@uni-heidelberg.de

https://doi.org/10.1016/j.chempr.2021.05.002

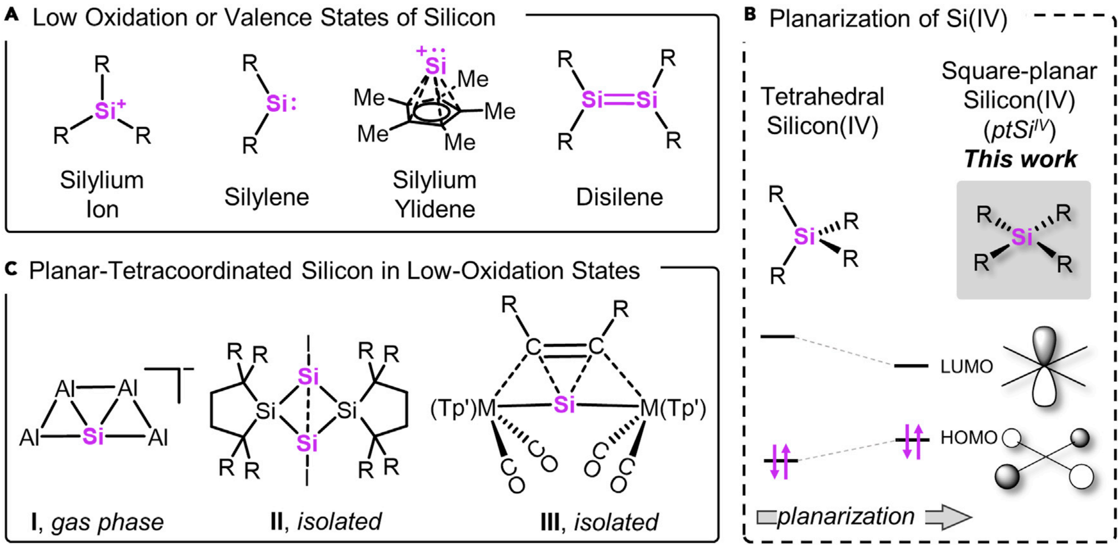

Figure 1. Molecular silicon complexes

(A) Selection of previously isolated low-oxidation or low-valent states of silicon.

(B) Reported compounds with planar-tetracoordinated silicon in low-oxidation states (M = W, Mo).

(C) Planarization of normal-valent silicon(IV), the effected changes of the frontier molecular orbital energy, and the representation of HOMO and LUMO in *ptSi*^{IV}.

The UV-vis spectrum of **2** in CH_2Cl_2 (69.5 μM) showed strong absorption bands at 301 and 360 nm, with a pronounced shoulder reaching into the visible range (Figure 3A). This observation contrasts with the non-chromophoric nature of every other tetracoordinated silane, e.g., the literature known tetra(N-pyrrolyl)silane **3** (Figure 3B),[59] and even with that of tris(N-pyrrolyl)borane,[60] but indicates an unusually small HOMO-LUMO gap.

Redox chemistry and bond activation with 2

Cyclic voltammetry revealed an irreversible reduction peak for **2** at a potential of –1.83 V versus Fc/Fc^+, which was assigned to the formation of the silicon-centered radical anion $\mathbf{2}^{\bullet-}$ (Figure S36). This reduction potential is exceptionally mild for silicon(IV), but in the range of electron-deficient boranes such as $B(C_6F_5)_3$.[61,62] Indeed, tetracoordinated silicon radical anions remain elusive as they are presumed to be powerful electron donors, even stronger than alkali metals.[63] A chemical reduction of **2** was attempted with decamethylcobaltocene (−1.9 V versus Fc/Fc^+) as a reducing agent to support the electroanalytical results. Performed in C_6D_6, a rapid reaction to a mixture of unidentified products indicated a redox process to happen. Performed in CD_2Cl_2 as the solvent, conversion to the chlorido silicate $[CoCp^*_2]$[**1**] was observed (Figure S16). This finding supports the formation of a silicon-centered radical anion that abstracts a chlorine atom from the solvent (see Figure S39 for the computed spin density of $\mathbf{2}^{\bullet-}$). Hence, planarization enables silicon-based radical chemistry under mildly reducing conditions.

To probe the potentially small HOMO-LUMO gap of the planar silane in bond activation, a solution of **2** in CD_2Cl_2 was subjected to phenylacetylene at room temperature. A rapid and quantitative silicon-ligand cooperative 1,2-addition of **2** to the carbon-carbon triple bond was verified by the occurrence of characteristic ^{1}H- and ^{29}Si-NMR spectroscopic patterns and mass spectrometry (Figures 3C and S19–S23). This interpretation was corroborated computationally for the model octamethyl-calix[4]pyrrolato silane by favorable thermodynamics (ΔG = −117 kJ mol^{-1}) and a low reaction barrier ($\Delta G^{\ddagger}$ = 61 kJ

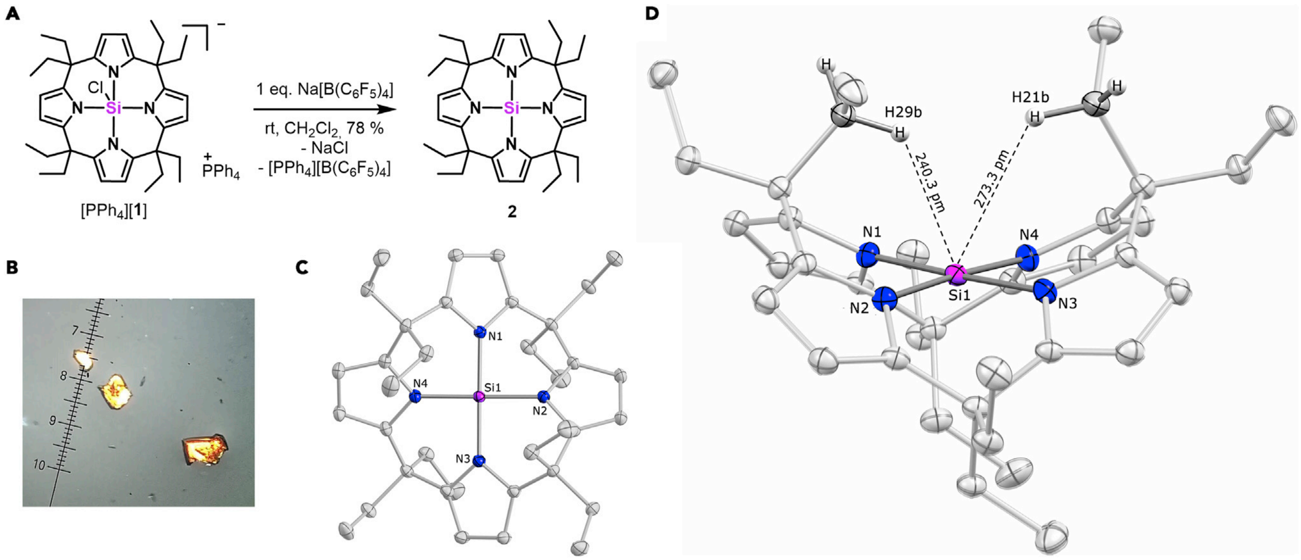

Figure 2. Synthesis and molecular structure of 2

(A) Synthesis of **2**.

(B) Microscopic photograph of crystals of **2**.

(C) Top-view of molecular structure of **2** as obtained by SCXRD/Hirshfeld atom refinement.

(D) Side-view of the molecular structure of **2**, showing hydrogen-silicon distances. Hydrogen atoms are refined freely and anisotropically, depicted at fixed positions, other atoms as thermal displacement ellipsoids of 50% probability. Selected bond distances (pm) and angles (°): N1–Si 178.84(5), N2–Si 179.26(5), N3–Si 179.90(4), N4–Si 178.81(4), H21b–Si 273.3(8), H29b–Si 240.3(8), H27b–Si 258.5(8), H35b–Si 296.3(8), N1–Si–N3 178.19(2), N2–Si–N4 176.08(2), N1–Si–N2 90.15(2), N2–Si–N3 89.28(2), N3–Si–N4 90.43(2), N4–Si–N1 90.26(2).

mol^{-1}) (see Figure S46). It represents an unprecedented, spontaneous carbosilylation of an alkyne with high synthetic potential, and the first example of silicon-element ligand co-operativity in its classical sense.[64]

Quantum chemical analysis of the electronic structure and spectroscopic features of 2

All experimental and spectroscopic results point to a unique electronic structure of **2**, which was thus analyzed by computational methods. Structure optimization of **2** validates the square-planar silicon (N–Si–N = 177°) as the energetic minimum. The LUMO in **2** has a strongly localized p_z-character at silicon with an energy lower by 2.4 eV than the tetrahedral **3** (Figure 3B). This low-lying LUMO explains the experimentally observed uptake of an electron in the redox studies. Indeed, the computed electron affinity for **2** (165 kJ mol^{-1}) is substantially higher than for tetrahedral **3** (6 kJ mol^{-1}). The HOMO of **2** is located at the ligand's π-system, and its energy is higher in comparison with **3** by 0.9 V. The reduced HOMO-LUMO gap rationalizes the light absorption in the visible range, and the time-dependent density functional theory (TDDFT) computed UV-vis spectrum is in line with the experimental one (Figure 3A). Inspection of the natural transition orbitals assigns the band at 360 nm to a $\pi(C{=}C_{pyrrole}) \rightarrow Si(p_z)$ charge transfer (HOMO/LUMO) and the more intense band at 301 nm as $N(LP) \rightarrow Si(p_z)$ charge transfer (HOMO-4/LUMO) (Figures 3B and S45). Those transitions are absent in the UV-vis spectrum of tetrahedral **3**, which shows ligand-based $\pi \rightarrow \pi^*$ excitation as the lowest energy transition (190 nm). Such a ligand-element charge transfer is very unusual for light p-block elements and underscores the π-donor stabilization in the square-planar silicon state.[39,42] This effect is also corroborated by natural bond orbital (NBO) analysis, which finds substantial π-delocalization of the nitrogen atom lone pair into the $Si(p_z)$ acceptor orbital, amounting to roughly 10% of the total Si–N bond interaction energy (Table S6).

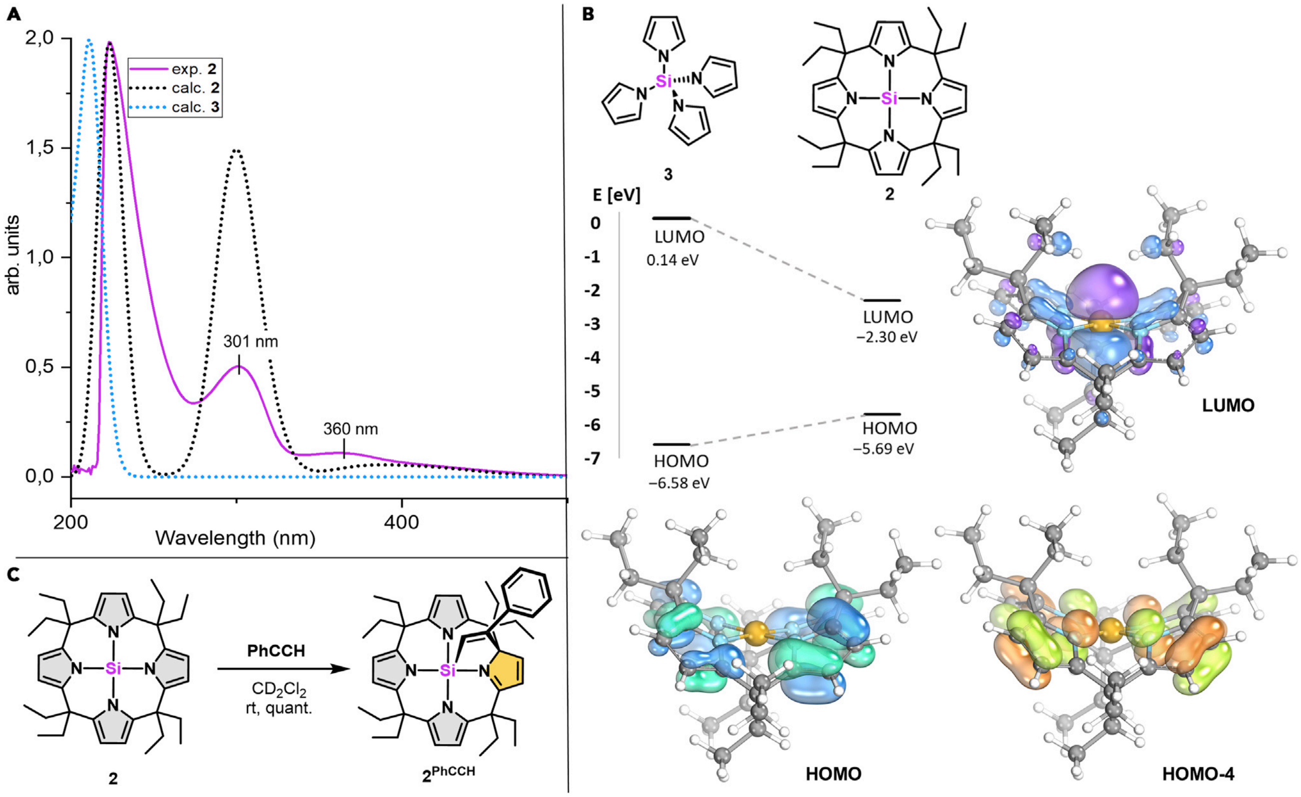

Figure 3. Experimental and computed properties of 2

(A) Experimental UV-vis spectrum of **2** (purple curve) in CH_2Cl_2 (69.5 μM), calculated (CAM-B3LYP/def2-TZVPP, shifted by +22 nm) curves of **2** (black curve), and **3** (blue curve).

(B) Energies and isodensity plots of the frontier molecular Kohn-Sham orbitals of **2** and **3**.

(C) Reactivity of **2** observed with phenylacetylene.

Last, the agostic or σ-donor type interaction of the endo-methylene C–H bonds with the square-planar silicon in **2** was considered computationally. Although the experimentally observed methylene group tilting was not found in the DFT-computed global minimum structure, the red-shifted IR bands for the endo-methylene C–H bonds were reproduced in the computed IR frequencies (Figure S38). Moreover, quantum theory of atoms in molecules (QTAIM) on the experimental SCXRD-HAR structure's electron density revealed a bond critical point between the inwards tilted H29b and the silicon center ($\rho(r) = 0.02$ a.u., Figure S42), in line with weak but traceable interactions also found for d^0-metals.[54] Further, NBO analysis disclosed significant $\sigma_{CH} \rightarrow Si(p_z)$ second-order perturbation energies (38 kJ mol^{-1}) and systematically depleted NBOs of the inward σ_{CH} bond in comparison with the other methylene CH-bond (Figure S40; Table S5). Ultimately, DLPNO-CCSD(T)/def2-QZVPP computations yielded an interaction enthalpy of −14 kJ mol^{-1} between ethane and the hypothetical octaprotio-calix[4]pyrrolato silane **4** (Figure S43; Table S7). The very low lying, silicon-centered LUMO in **2** provides a strong driving force for this orbital-controlled interaction, potentially supported by London dispersive attraction.[65]

Conclusion

The first square-planar silicon(IV) species **2** is isolated and characterized. The compound undergoes unprecedented silicon redox chemistry, light-induced ligand-element charge transfer, and bond activation in element-ligand cooperative fashion. Hence, the structural-, spectroscopic-, and reactivity features of **2** confirm a LUMO

substantially lowered in energy and a HOMO-LUMO gap reduced by 50%, provoked by forcing a Si(IV)-tetrahedron into a planar structure. Small HOMO-LUMO gaps are the basis for the peculiar reactivity of d-block metals, low-valent p-block compounds, or frustrated Lewis pairs.[48] This work establishes a fourth pillar to reach this goal. It endows the second most abundant element with new potential for catalysis, photochemistry, and materials science, while staying in the oxidation state that seemed exhaustively understood.

EXPERIMENTAL PROCEDURES

Resource availability

Lead contact

Further information and requests for resources and reagents should be directed to and will be fulfilled by the lead contact, Lutz Greb (greb@uni-heidelberg.de).

Materials availability

All unique/stable reagents generated in this study are available from the lead contact with a completed materials transfer agreement.

Data and code availability

Crystallographic data have been deposited in the Cambridge Crystallographic Data Center (CCDC) under accession numbers CCDC: 2042938, 2042939, 2042940, and 2042941. These data can be obtained free of charge from the CCDC at http://www.ccdc.cam.ac.uk/data_request/cif.

Synthesis and analytical data of 2

Rigerously donor free sodium tetrakis(pentafluorophenyl)borate (68.4 mg, 28.5 μmol, 1.10 equiv) was added to a solution of [PPh_4][**1**] (66.0 mg, 70.2 μmol, 1.00 equiv) in 4 ml CH_2Cl_2 at ambient temperature, and stirred for further 12 h, during which it turned yellow. The solvent was removed under reduced pressure, the residue suspended in 10 ml *n*-hexane, stirred for 2 h, and filtered. The solvent of the filtrate was removed, and the residue was dissolved in 10 ml *n*-hexane, stirred for 24 h and, filtered. The clear yellow solution was stirred for another 24 h to drive the elimination of NaCl to completeness. The yellow solution was filtered again, and the solvent was removed. After drying *in vacuo,* the neutral silane **2** was obtained as a yellow solid 78% yield (30.9 mg, 54.7 μmol). Orange crystals of **2** were obtained by storing a concentrated solution of **2** in CH_2Cl_2 for 7 days at -40°C. Slow decomposition in CD_2Cl_2 and C_6D_6 was observed over 4 days at room temperature. Remark: the first step in this reaction is the cation exchange from PPh_4^+ to Na^+. The SCXRD of this intermediate $[Na]_4[\mathbf{1}]_4$ was obtained by storing a concentrated solution of [PPh_4][**1**] and $NaBArF_{20}$ in DCM for 5 days at room temperature without further workup (CCDC: 2042940). **^{1}H NMR** (CD_2Cl_2, 400 MHz, 295 K) δ[ppm]: 5.89 (s, 8H), 2.04 (q, $^3J_{HH}$ = 7.4 Hz, 8H, $CH_{2\ Ethyl}$), 1.93 (q, $^3J_{HH}$ = 7.4 Hz, 8H, $CH_{2\ Ethyl}$), 1.09 (t, $^3J_{HH}$ = 7.4 Hz, 12H, $CH_{3\ Ethyl}$), 0.66 (t, $^3J_{HH}$ = 7.4 Hz, 12H, $CH_{3\ Ethyl}$). **^{13}C{^{1}H}NMR** (CD_2Cl_2, 100 MHz, 295 K) δ[ppm]: 143.9 (*C1*), 106.5 (*C2*), 45.9 ($CH_{2\ Ethyl}$), 42.4 (*C3*), 24.8 ($CH_{2\ Ethyl}$), 9.4 ($CH_{3\ Ethyl}$), 9.3 ($CH_{3\ Ethyl}$). **^{29}Si HMBC NMR** (CD_2Cl_2, 400 MHz, 295 K): −55.6 ppm.

Mass spectrometry

[ESI]: $C_{36}H_{48}N_4Si*OH^-$: calc. 581.3675 exp. 581.3761. [MALDI, DFTB-Matrix]: $C_{36}H_{48}N_4Si*H_3O^+$: calc. 583.383 exp. 583.397.

IR spectroscopy $\tilde{\nu}$[cm^{-1}]

3,112 (w), 2,963 (s), 2,931 (s), 2,872 (s), 2,815 (w), 1,344 (w), 1,514 (m), 1,455 (s), 1,378 (m), 1,364 (m), 1,322 (w), 1,297 (w), 1,278 (w), 1,234 (s), 1,132 (s), 1,087 (s), 973 (s), 953 (m), 926 (w), 857 (w), 736 (s), 713 (m), 681 (w).

Crystal data for [2]

$C_{36}H_{48}N_4Si$, M = 564.87, monoclinic, $P2_1/n$ (no. 14), a = 10.3052(4), b = 19.3263(7), c = 14.9876(7) Å, α = 90, β = 94.396(2), γ = 90, V = 2,976.2(2) $Å^3$, Z = 4, D_c = 1.261 g cm^{-3}, μ(Mo-Kα) = 0.112 mm^{-1}, $2\theta_{max}$ = 61°, T = 100 K, orange blocks, Bruker APEX-II CCD; 9,086 independent measured reflections (R_{int} = 0.0505), R_1(obs) = 0.0380, wR_2(all) = 0.1046, 406 parameters. NoSphereA2-solution: R_1(obs) = 0.0203, wR_2(all) = 0.0436, 802 parameters. CCDC: 2042941(ShelXl solution)/2042939 (HAR/NoSpherA2-solution).

SUPPLEMENTAL INFORMATION

Supplemental information can be found online at https://doi.org/10.1016/j.chempr.2021.05.002.

ACKNOWLEDGMENTS

We thank Prof. H.-J. Himmel for his support, Prof. M. Enders and Dr. Dragos Rosca for discussion, and Prof. H. Wadepohl and Dr. M. Schorpp for advice with SCXRD. Prof. S. Grabowsky and Florian Kleemiss are acknowledged for providing NoSphereA2 and valuable advice. Financial support was provided by the European Research Council (ERC) under the European Union's Horizon 2020 research and innovation program (grant agreement no. 948708), the DFG (GR5007/1-1), and the Foundation of German Business (sdw, F.E.). The BWFor/BWUniCluster, funded by the DFG, is acknowledged for computational resources.

AUTHOR CONTRIBUTIONS

L.G. and F.E. designed and conducted experiments and computations and prepared the manuscript. L.G. supervised the project.

DECLARATION OF INTERESTS

The authors declare no competing interests.

Received: December 17, 2020
Revised: March 1, 2021
Accepted: May 4, 2021
Published: June 9, 2021

REFERENCES

1. Lide, D.R. (2000). CRC Handbook of Chemistry and Physics, Eighty-First Edition (CRC Press).

2. Kim, K.C., Reed, C.A., Elliott, D.W., Mueller, L.J., Tham, F., Lin, L., and Lambert, J.B. (2002). Crystallographic evidence for a free silylium ion. Science *297*, 825–827.

3. Wu, Q., Irran, E., Müller, R., Kaupp, M., Klare, H.F.T., and Oestreich, M. (2019). Characterization of hydrogen-substituted silylium ions in the condensed phase. Science *365*, 168–172.

4. Denk, M., Lennon, R., Hayashi, R., West, R., Belyakov, A.V., Verne, H.P., Haaland, A., Wagner, M., and Metzler, N. (1994). Synthesis and structure of a stable silylene. J. Am. Chem. Soc. *116*, 2691–2692.

5. Ghadwal, R.S., Roesky, H.W., Merkel, S., Henn, J., and Stalke, D. (2009). Lewis base stabilized dichlorosilylene. Angew. Chem. Int. Ed. Engl. *48*, 5683–5686.

6. Protchenko, A.V., Birjkumar, K.H., Dange, D., Schwarz, A.D., Vidovic, D., Jones, C., Kaltsoyannis, N., Mountford, P., and Aldridge, S. (2012). A stable two-coordinate acyclic silylene. J. Am. Chem. Soc. *134*, 6500–6503.

7. Jutzi, P., Mix, A., Rummel, B., Schoeller, W.W., Neumann, B., and Stammler, H.G. (2004). The $(Me_5C_5)Si^+$ cation: a stable derivative of HSi^+. Science *305*, 849–851.

8. Xiong, Y., Yao, S., Inoue, S., Irran, E., and Driess, M. (2012). The elusive silyliumylidene [ClSi:]+ and silathionium [ClSi=S]+ cations stabilized by bis(iminophosphorane) chelate ligand. Angew. Chem. Int. Ed. Engl. *51*, 10074–10077.

9. Filippou, A.C., Lebedev, Y.N., Chernov, O., Straßmann, M., and Schnakenburg, G. (2013). Silicon(II) coordination chemistry: N-heterocyclic carbene complexes of Si2+ and SiI+. Angew. Chem. Int. Ed. Engl. *52*, 6974–6978.

10. Hinz, A. (2020). A mono-substituted silicon(II) cation: a crystalline "supersilylene". Angew. Chem. Int. Ed. Engl. *59*, 19065–19069.

11. West, R., Fink, M.J., and Michl, J. (1981). Tetramesityldisilene, a stable compound containing a silicon-silicon double bond. Science *214*, 1343–1344.

12. Sekiguchi, A., Kinjo, R., and Ichinohe, M. (2004). A stable compound containing a silicon-silicon triple bond. Science *305*, 1755–1757.

13. Ishida, S., Iwamoto, T., Kabuto, C., and Kira, M. (2003). A stable silicon-based allene analogue with a formally sp-hybridized silicon atom. Nature *421*, 725–727.

14. Mondal, K.C., Roesky, H.W., Schwarzer, M.C., Frenking, G., Niepötter, B., Wolf, H., Herbst-Irmer, R., and Stalke, D. (2013). A stable singlet

biradicaloid siladicarbene: (L:)$_2$Si. Angew. Chem. Int. Ed. Engl. *52*, 2963–2967.

15. Xiong, Y., Yao, S., Inoue, S., Epping, J.D., and Driess, M. (2013). A cyclic silylone ("siladicarbene") with an electron-rich silicon(0) atom. Angew. Chem. Int. Ed. Engl. *52*, 7147–7150.

16. Lee, V.Y. (2017). Organosilicon Compounds (Elsevier Academic Press).

17. Allemann, O., Duttwyler, S., Romanato, P., Baldridge, K.K., and Siegel, J.S. (2011). Proton-catalyzed, silane-fueled Friedel-Crafts coupling of fluoroarenes. Science *332*, 574–577.

18. Shao, B., Bagdasarian, A.L., Popov, S., and Nelson, H.M. (2017). Arylation of hydrocarbons enabled by organosilicon reagents and weakly coordinating anions. Science *355*, 1403–1407.

19. Hiyama, T., and Oestreich, M. (2019). Organosilicon Chemistry: Novel Approaches and Reactions (Wiley-VCH Verlag).

20. Shan, C., Yao, S., and Driess, M. (2020). Where silylene–silicon centres matter in the activation of small molecules. Chem. Soc. Rev. *49*, 6733–6754.

21. Hanusch, F., Groll, L., and Inoue, S. (2021). Recent advances of group 14 dimetallenes and dimetallynes in bond activation and catalysis. Chem. Sci. *12*, 2001–2015.

22. Albright, T.A., Burdett, J.K., and Whangbo, M.-H. (2013). Orbital Interactions in Chemistry (Wiley).

23. Dunn, N.L., Ha, M., and Radosevich, A.T. (2012). Main group redox catalysis: reversible PIII/PV redox cycling at a phosphorus platform. J. Am. Chem. Soc. *134*, 11330–11333.

24. Thompson, E.J., Myers, T.W., and Berben, L.A. (2014). Synthesis of square-planar aluminum(III) complexes. Angew. Chem. Int. Ed. Engl. *53*, 14132–14134.

25. Robinson, T.P., De Rosa, D.M., Aldridge, S., and Goicoechea, J.M. (2015). E-H bond activation of ammonia and water by a geometrically constrained phosphorus(III) compound. Angew. Chem. Int. Ed. *54*, 13758–13763.

26. Hentschel, A., Brand, A., Wegener, P., and Uhl, W. (2018). A sterically constrained tricyclic PC3 phosphine: coordination behavior and insertion of chalcogen atoms into P−C Bonds. Angew. Chem. Int. Ed. Engl. *57*, 832–835.

27. Volodarsky, S., and Dobrovetsky, R. (2018). Ambiphilic geometrically constrained phosphenium cation. Chem. Commun. (Camb) *54*, 6931–6934.

28. Kindervater, M.B., Marczenko, K.M., Werner-Zwanziger, U., and Chitnis, S.S. (2019). A redox-confused bismuth(I/III) triamide with a T-shaped planar ground state. Angew. Chem. Int. Ed. Engl. *58*, 7850–7855.

29. Nelson, S.G., Kim, B.-K., and Peelen, T.J. (2000). Lewis acidity expressed in neutral electron-rich aluminum(III) complexes: an example of ligand-defined catalysis. J. Am. Chem. Soc. *122*, 9318–9319.

30. Ben Saida, A., Chardon, A., Osi, A., Tumanov, N., Wouters, J., Adjieufack, A.I., Champagne, B., and Berionni, G. (2019). Pushing the Lewis acidity boundaries of boron compounds with non-planar triarylboranes derived from triptycenes. Angew. Chem. Int. Ed. Engl. *58*, 16889–16893.

31. Boldyrev, A.I., Li, X., and Wang, L.S. (2000). Experimental observation of pentaatomic tetracoordinate planar Si- and Ge-containing molecules: MAl4− and MAl4. Angew. Chem. Int. Ed. Engl. *39*, 3307–3310.

32. Nukazawa, T., and Iwamoto, T. (2020). An isolable tetrasilicon analogue of a planar bicyclo[1.1.0]butane with π-type single-bonding character. J. Am. Chem. Soc. *142*, 9920–9924.

33. Cowley, M.J., Huch, V., and Scheschkewitz, D. (2014). Donor–acceptor adducts of a 1,3-disila-2-oxyallyl zwitterion. Chemistry *20*, 9221–9224.

34. Ghana, P., Rump, J., Schnakenburg, G., Arz, M.I., and Filippou, A.C. (2021). Planar tetracoordinated silicon (ptSi): room-temperature stable compounds containing anti-van't Hoff/Le Bel silicon. J. Am. Chem. Soc. *143*, 420–432.

35. Meyer, H., and Nagorsen, G. (1979). Structure and reactivity of the orthocarbonic and orthosilicic acid esters of pyrocatechol. Angew. Chem. Int. Ed. Engl. *18*, 551–553.

36. Schomburg, D. (1983). Strong distortion of the tetrahedral geometry in a Spirosilicate: molecular structure of bis(tetramethylethylenedioxy)silane. Angew. Chem. Int. Ed. Engl. *22*, 65.

37. Ding, B., Keese, R., and Stoeckli-Evans, H. (1999). First synthesis and structure of a tetraazasilafenestrane. Angew. Chem. Int. Ed. Engl. *38*, 375–376.

38. Dunitz, J.D. (1980). Planar four-coordinated silicon? Angew. Chem. Int. Ed. Engl. *19*, 1034.

39. Krogh-Jespersen, M.B., Chandrasekhar, J., Wuerthwein, E.U., Collins, J.B., and von Ragué Schleyer, P. (1980). Molecular orbital study of tetrahedral, planar, and pyramidal structures of the isoelectronic series BH4-, CH4, NH4+, AlH4-, SiH4, and PH4+. J. Am. Chem. Soc. *102*, 2263–2268.

40. Ebner, F., and Greb, L. (2018). Calix[4]pyrrole hydridosilicate: the elusive planar tetracoordinate silicon imparts striking stability to its anionic silicon hydride. J. Am. Chem. Soc. *140*, 17409–17412.

41. Yang, L.M., Ganz, E., Chen, Z., Wang, Z.X., and Schleyer, Pv. (2015). Four decades of the chemistry of planar hypercoordinate compounds. Angew. Chem. Int. Ed. Engl. *54*, 9468–9501.

42. Würthwein, E.-U., and von Ragué Schleyer, P. (1979). Planar tetracoordinate silicon. Angew. Chem. Int. Ed. Engl. *18*, 553–554.

43. Boldyrev, A.I., von Ragué Schleyer, P., and Keese, R. (1992). Planar tetracoordinated silicon and phosphorus geometries in poiynitrogen-substituted [5.5.5.5]Fenestrenes. Mendeleev Commun *2*, 93–95.

44. Li, Y., Li, F., Zhou, Z., and Chen, Z. (2011). SiC2 silagraphene and its one-dimensional derivatives: where planar tetracoordinate silicon happens. J. Am. Chem. Soc. *133*, 900–908.

45. Sun, M.J., Cao, X., and Cao, Z. (2018). Stabilization of planar tetra-coordinate silicon in a 2D-layered extended system and design of a high-capacity anode material for Li-ion batteries. Nanoscale *10*, 10450–10458.

46. Wang, Y., Li, Y., and Chen, Z. (2020). Planar hypercoordinate motifs in two-dimensional materials. Acc. Chem. Res. *53*, 887–895.

47. Zhang, Y., Zhang, C., Mo, Y., and Cao, Z. (2021). Planar tetracoordinate silicon in organic molecules as carbenoid-type amphoteric centers: a computational study. Chemistry *27*, 1402–1409.

48. Power, P.P. (2010). Main-group elements as transition metals. Nature *463*, 171–177.

49. Jacoby, D., Floriani, C., Chiesi-Villa, A., and Rizzoli, C. (1991). meso-octamethyl-porphyrinogen metal complexes: an entry to high valent unsaturated metal centres. J. Chem. Soc. Chem. Commun. 220–222.

50. Cuesta, L., and Sessler, J.L. (2009). π-metal complexes of tetrapyrrolic systems. A novel coordination mode in "porphyrin-like" chemistry. Chem. Soc. Rev. *38*, 2716–2729.

51. Woińska, M., Grabowsky, S., Dominiak, P.M., Woźniak, K., and Jayatilaka, D. (2016). Hydrogen atoms can be located accurately and precisely by x-ray crystallography. Sci. Adv. *2*, e1600192.

52. Ebner, F., Wadepohl, H., and Greb, L. (2019). Calix[4]pyrrole aluminate: A planar tetracoordinate aluminum(III) anion and its unusual Lewis acidity. J. Am. Chem. Soc. *141*, 18009–18012.

53. Brookhart, M., Green, M.L.H., and Parkin, G. (2007). Agostic interactions in transition metal compounds. Proc. Natl. Acad. Sci. USA *104*, 6908–6914.

54. Scherer, W., and McGrady, G.S. (2004). Agostic interactions in d0 metal alkyl complexes. Angew. Chem. Int. Ed. Engl. *43*, 1782–1806.

55. Houghton, A.Y., Hurmalainen, J., Mansikkamäki, A., Piers, W.E., and Tuononen, H.M. (2014). Direct observation of a borane–silane complex involved in frustrated Lewis-pair-mediated hydrosilylations. Nat. Chem. *6*, 983–988.

56. Liu, Y., Su, B., Dong, W., Li, Z.H., and Wang, H. (2019). Structural characterization of a boron(III) η2-σ-silane-complex. J. Am. Chem. Soc. *141*, 8358–8363.

57. Khalimon, A.Y., Lin, Z.H., Simionescu, R., Vyboishchikov, S.F., and Nikonov, G.I. (2007). Persistent silylium ions stabilized by polyagostic Si-H···Si interactions. Angew. Chem. Int. Ed. *46*, 4530–4533.

58. Dorsey, C.L., and Gabbaï, F.P. (2008). $R_3C{-}H \rightarrow SiFR_3$ agostic interaction. Organometallics *27*, 3065–3069.

59. Frenzel, A., Herbst-Irmer, R., Klingebiel, U., Noltemeyer, M., and Schäfer, M. (1995). Indolyl- und Pyrrolylsilane – synthese und Kristallstruktur / indolyl- and Pyrrolylsilanes –

CellPress
OPEN ACCESS

syntheses and crystal structures. Z. Naturforsch. B *50*, 1658–1664.

60. Wrackmeyer, B., Schwarze, B., and Milius, W. (1996). Molecular structures of tri-N-pyrrolylboranes and the dynamic behaviour of tri-N-indolylboranes in solution. Inorg. Chim. Acta *241*, 87–93.

61. Fuchigami, T. (1998). Electrochemistry of organosilicon compounds. The Chemistry of Organic Silicon Compounds, *Vol. 2* (John Wiley &Sons)), pp. 1187–1232.

62. Lawrence, E.J., Oganesyan, V.S., Wildgoose, G.G., and Ashley, A.E. (2013). Exploring the fate of the tris(pentafluorophenyl)borane radical anion in weakly coordinating solvents. Dalton Trans *42*, 782–789.

63. Smith, A.J., Young, A., Rohrbach, S., O'Connor, E.F., Allison, M., Wang, H.-S., Poole, D.L., Tuttle, T., and Murphy, J.A. (2017). Electron-transfer and hydride-transfer pathways in the Stoltz–Grubbs reducing system (KOtBu/Et3SiH). Angew. Chem. Int. Ed. *56*, 13747–13751.

64. Greb, L., Ebner, F., Ginzburg, Y., and Sigmund, L.M. (2020). Element-ligand cooperativity with p-block elements. Eur. J. Inorg. Chem. *2020*, 3030–3047.

65. Lu, Q., Neese, F., and Bistoni, G. (2018). Formation of agostic structures driven by London dispersion. Angew. Chem. Int. Ed. Engl. *57*, 4760–4764.

Chem, Volume *7*

Supplemental information

An isolable, crystalline complex of square-planar silicon(IV)

Fabian Ebner and Lutz Greb

Supplemental Analytic Data

NMR-Spectra

(*residual solvent peaks)

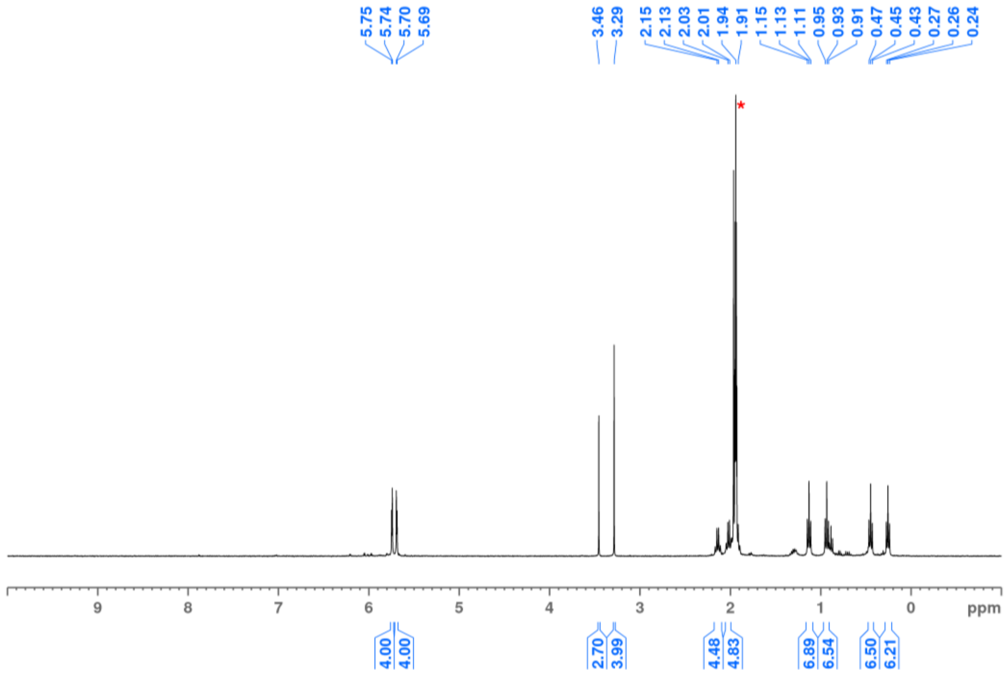

Figure S 1: ^{1}H NMR (ACN-d_3, 600 MHz, 295 K) spectrum of [Li(dme)][**1**].

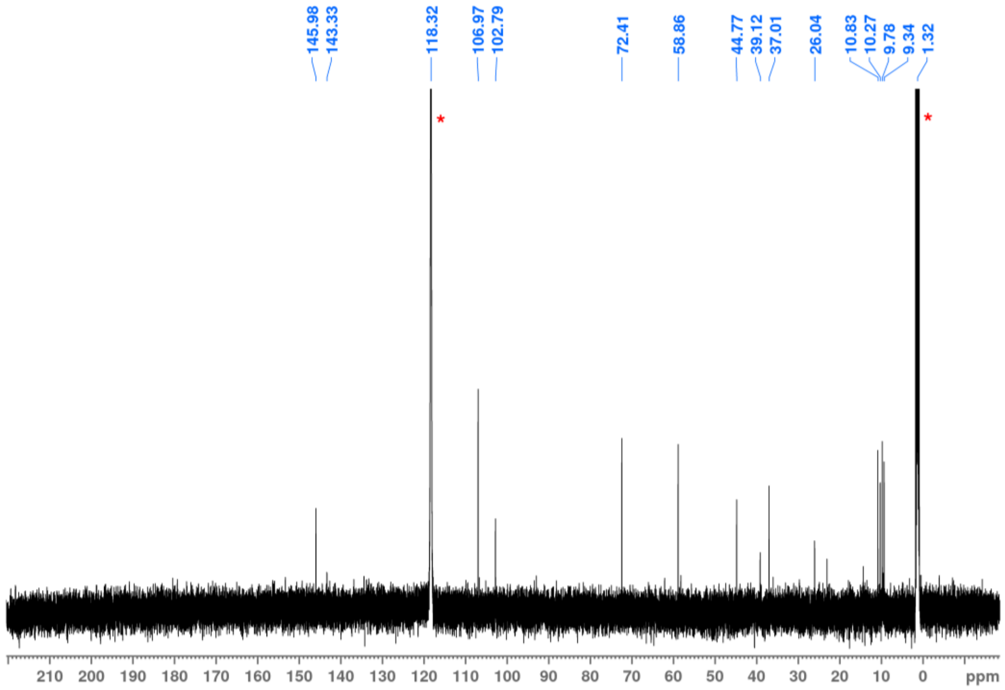

Figure S 2: ^{13}C NMR (ACN-d_3, 150 MHz, 295 K) spectrum of [Li(dme)][**1**].

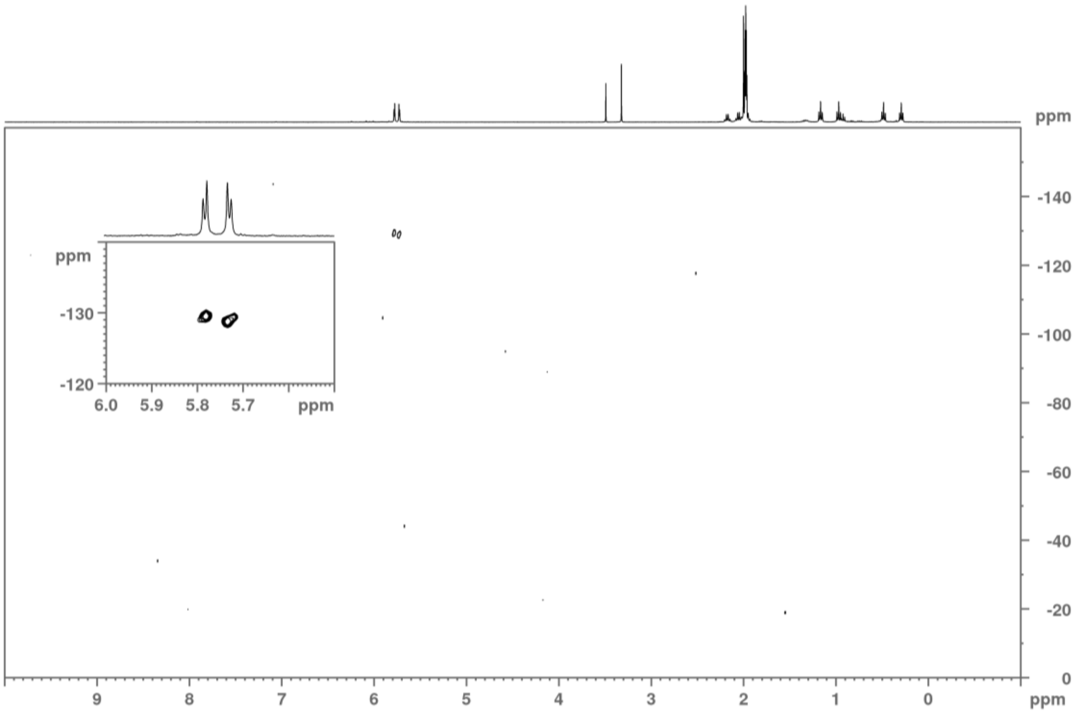

Figure S 3: ^{29}Si HMBC NMR (ACN-d_3, 600 MHz, 295 K) spectrum of [Li(dme)][**1**].

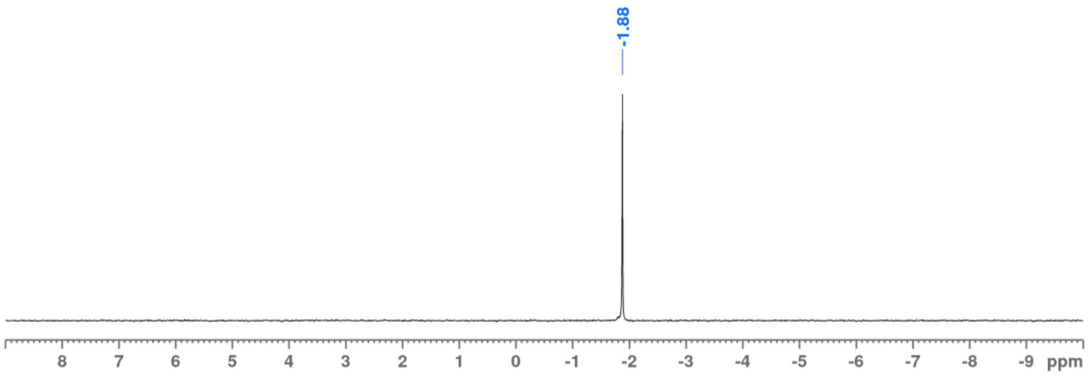

Figure S 4: ^{7}Li NMR (ACN-d_3, 155 MHz, 295 K) spectrum of [Li(dme)][**1**].

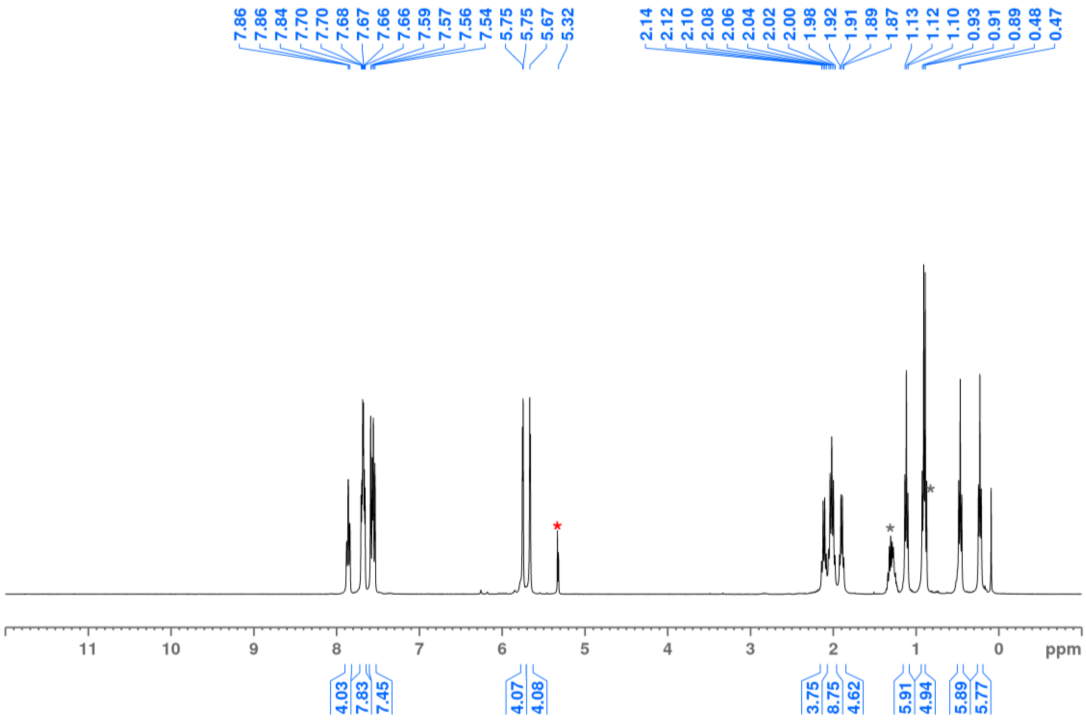

Figure S 5: ^{1}H NMR (DCM-d_2, 400 MHz, 295 K) spectrum of [PPh_4][**1**]. Gray asterisks mark residues of *n*-pentane.

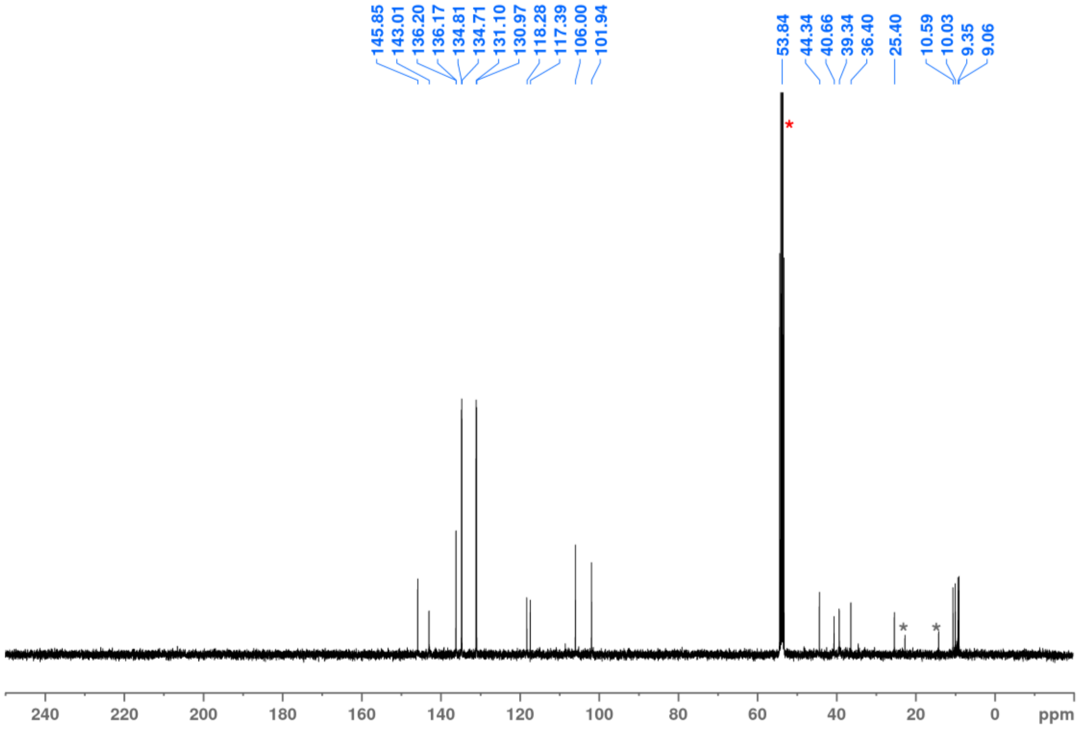

Figure S 6: ^{13}C NMR (DCM-d_2, 100 MHz, 295 K) spectrum of [PPh_4][**1**]. Gray asterisks mark residues of *n*-pentane.

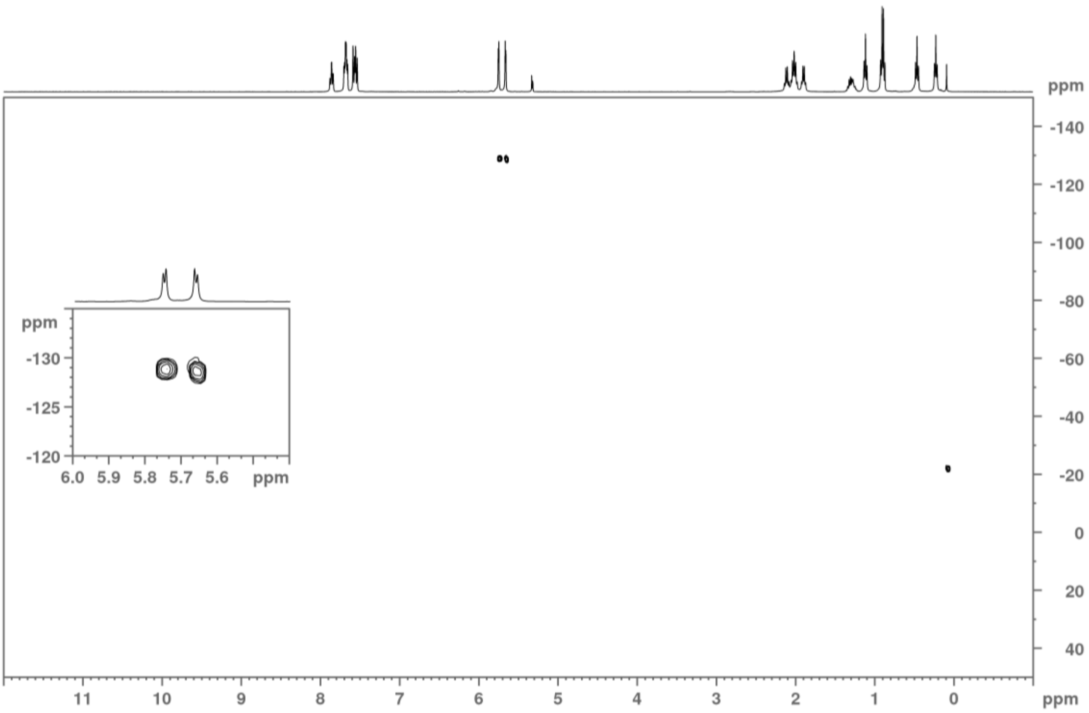

Figure S 7: ^{29}Si HMBC NMR (DCM-d_2, 400 MHz, 295 K) spectrum of [PPh_4][**1**]. Cross peak at δ_{29Si} = -23 ppm belongs to H grease.

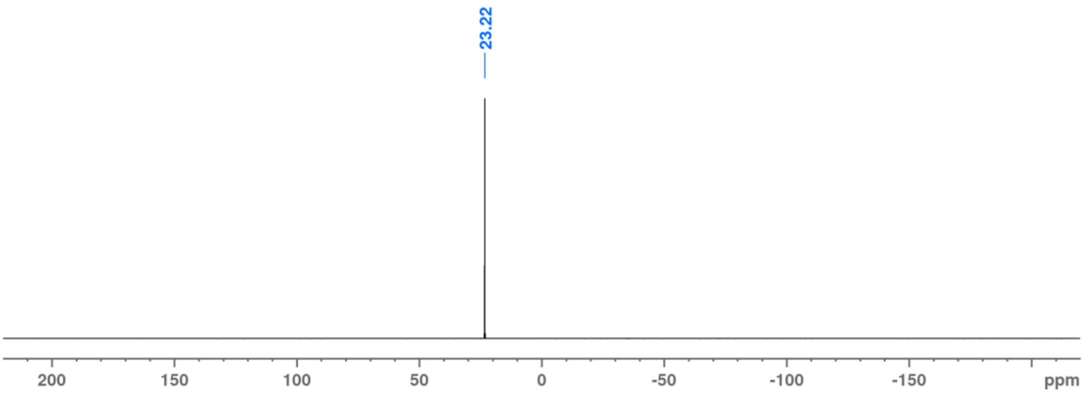

Figure S 8: ^{31}P NMR (DCM-d_2, 161 MHz, 295 K) spectrum of [PPh_4][**1**].

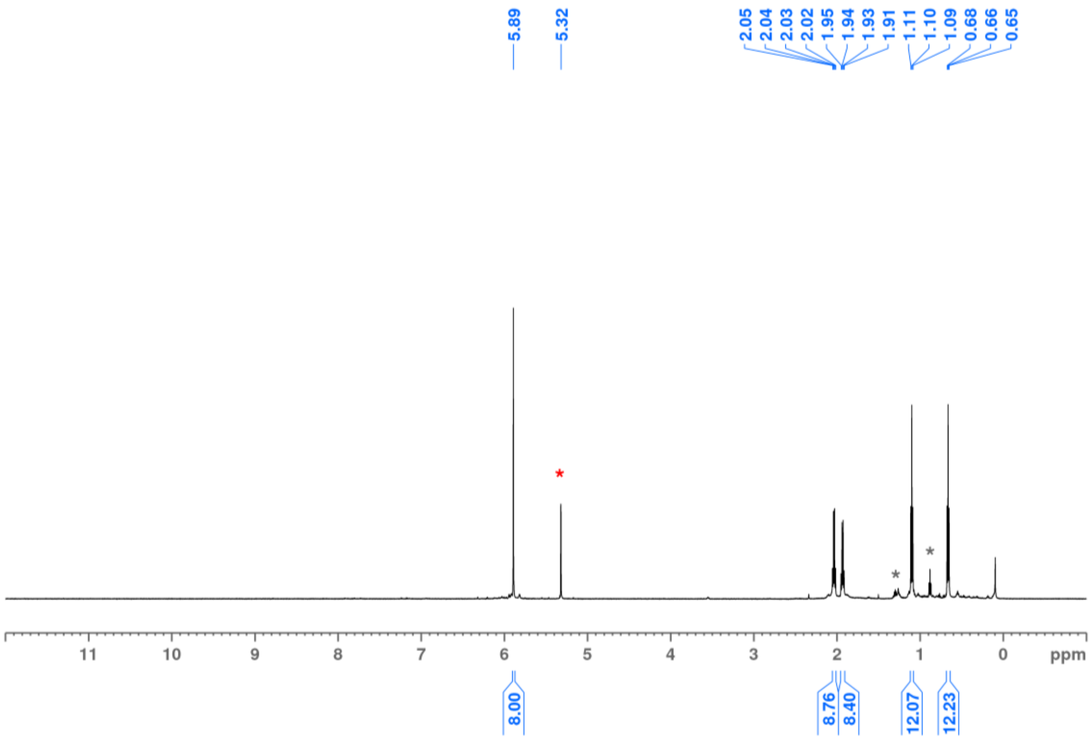

Figure S 9: ^{1}H NMR (DCM-d_2, 400 MHz, 295 K) spectrum of **2.** Gray asterisks mark residues of *n*-pentane.

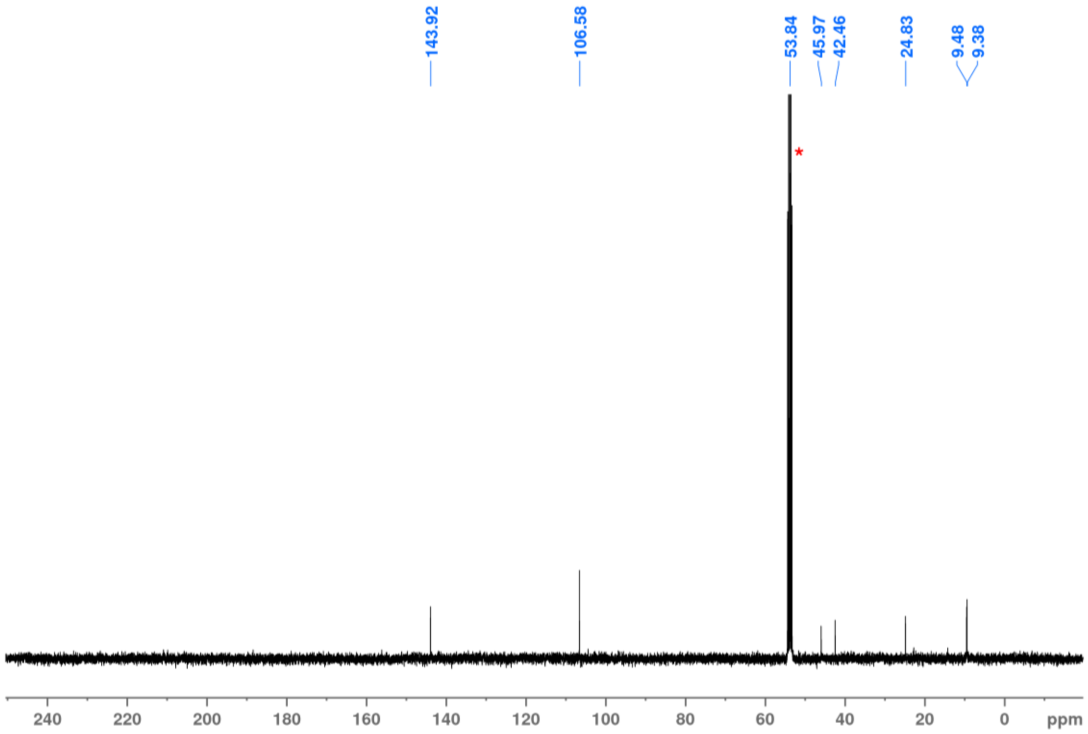

Figure S 10: ^{13}C NMR (DCM-d_2, 100 MHz, 295 K) spectrum of **2.**

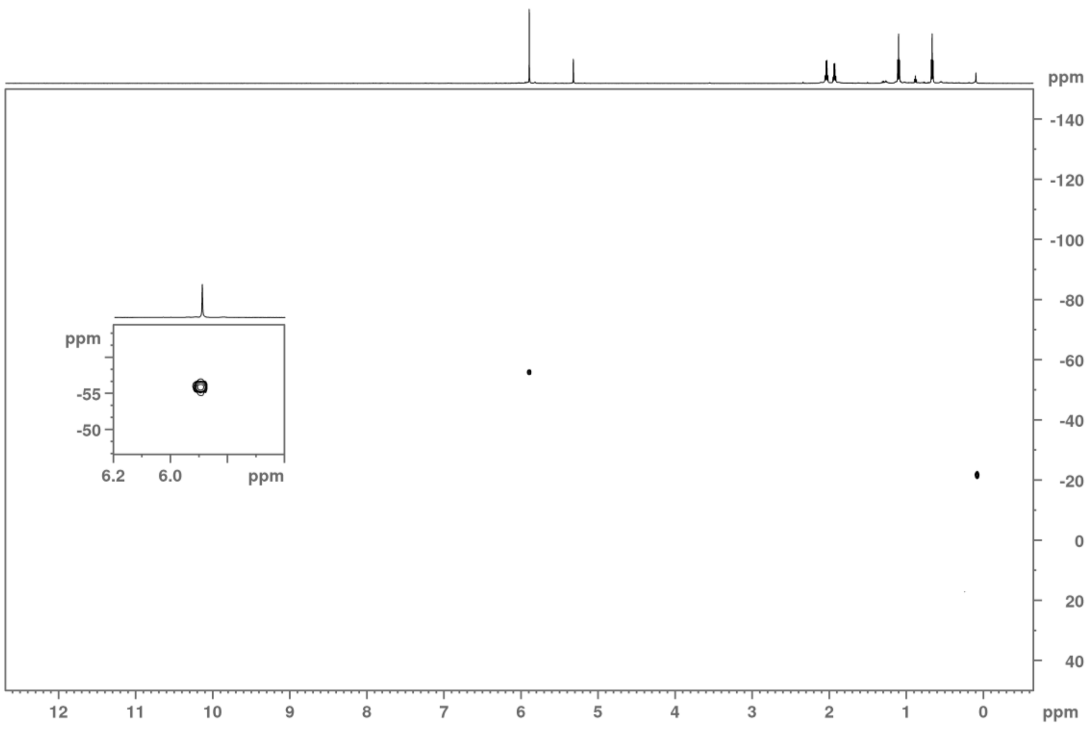

Figure S 11: ^{29}Si HMBC NMR (DCM-d$_2$, 400 MHz, 295 K) spectrum of **2**. Cross peak at δ_{29Si} = -23 ppm belongs to H grease.

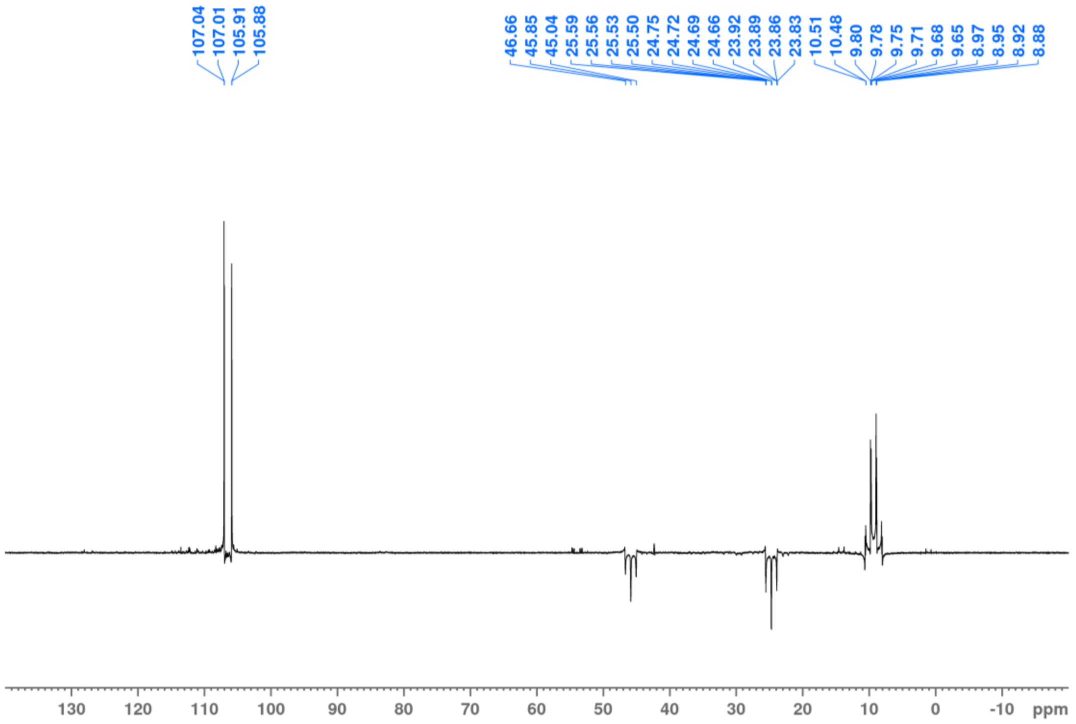

Figure S 12: ^{13}C DEPT135 ^{1}H coupled NMR spectrum (DCM-d$_2$, 150 MHz, 295 K) of **2**.

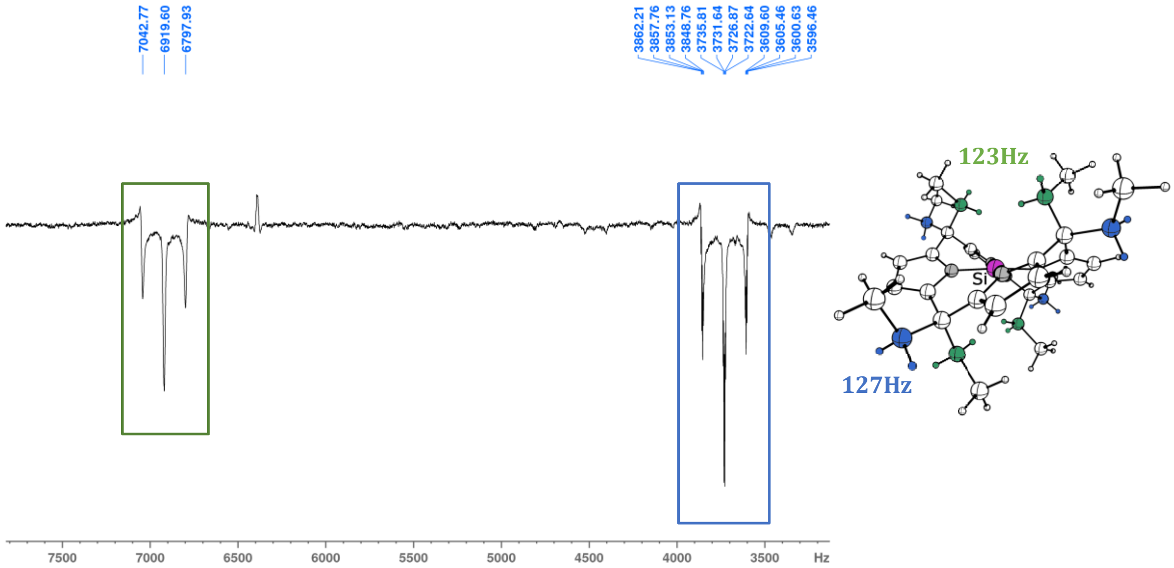

Figure S 13: Section of relevant peak splitting of the ^{13}C DEPT135 ^{1}H coupled NMR spectrum (DCM-d_2, 150 MHz, 295 K) of **2** shown in Figure S 12. Green color marks the endo-methylene group signals, blue color marks the exo-methylene group signals. Difference in coupling constant between exo- and endo-methylene groups is 4 Hz.

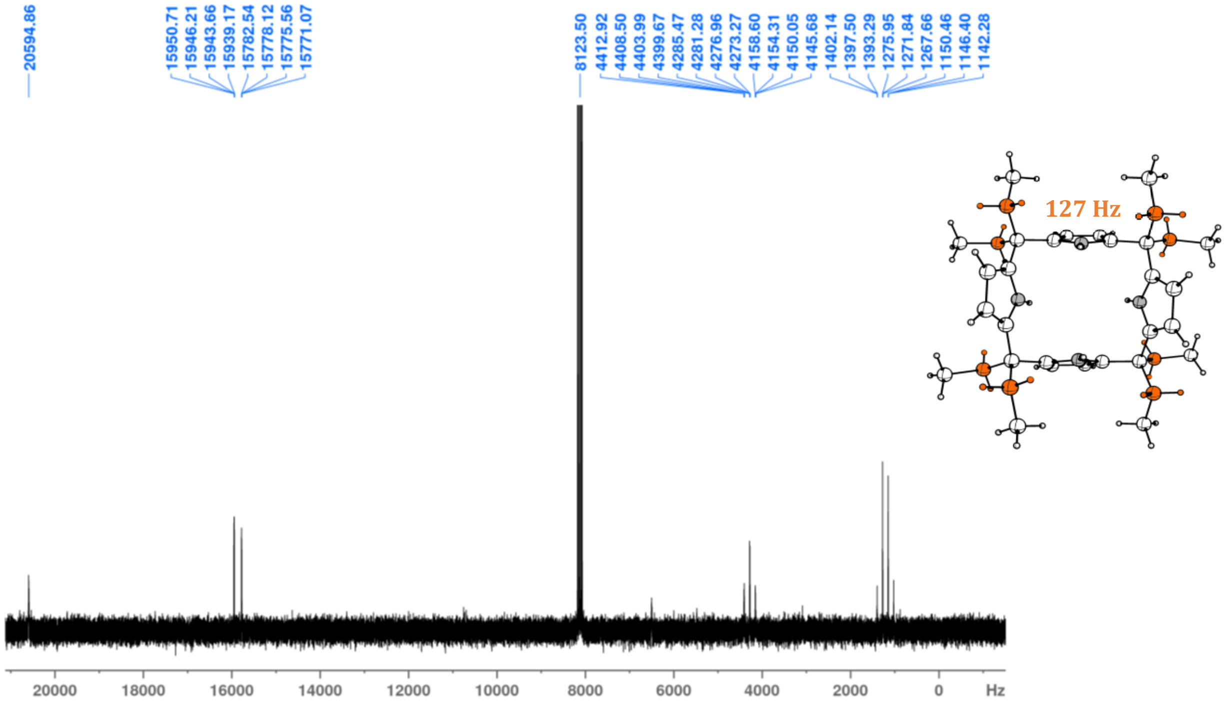

Figure S 14: ^{13}C ^{1}H coupled NMR spectrum (DCM-d_2, 150 MHz, 295 K) of free octaethyl-calix[4]pyrrole ligand.

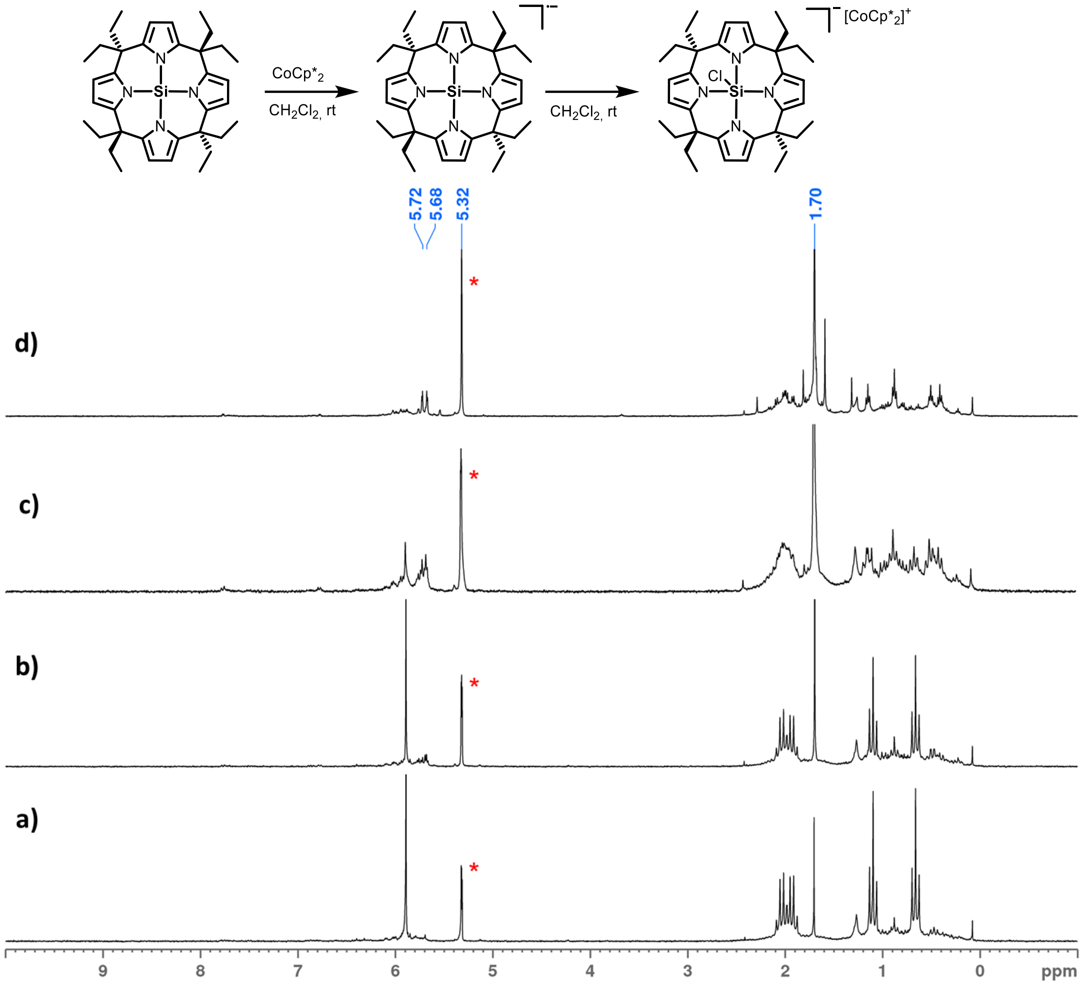

Figure S 15: ^1H NMR stack (DCM-d_2, 200 MHz, 295 K) of reduction progress after dddition of a) 0.1 eq, b) 0.2 eq, c) 0.6 eq and d) 1.0 eq of $CoCp_2^*$ to **2**. Formation of [**1**]$^-$ as the major product was indicated by the signals at δ = 5.7 ppm.

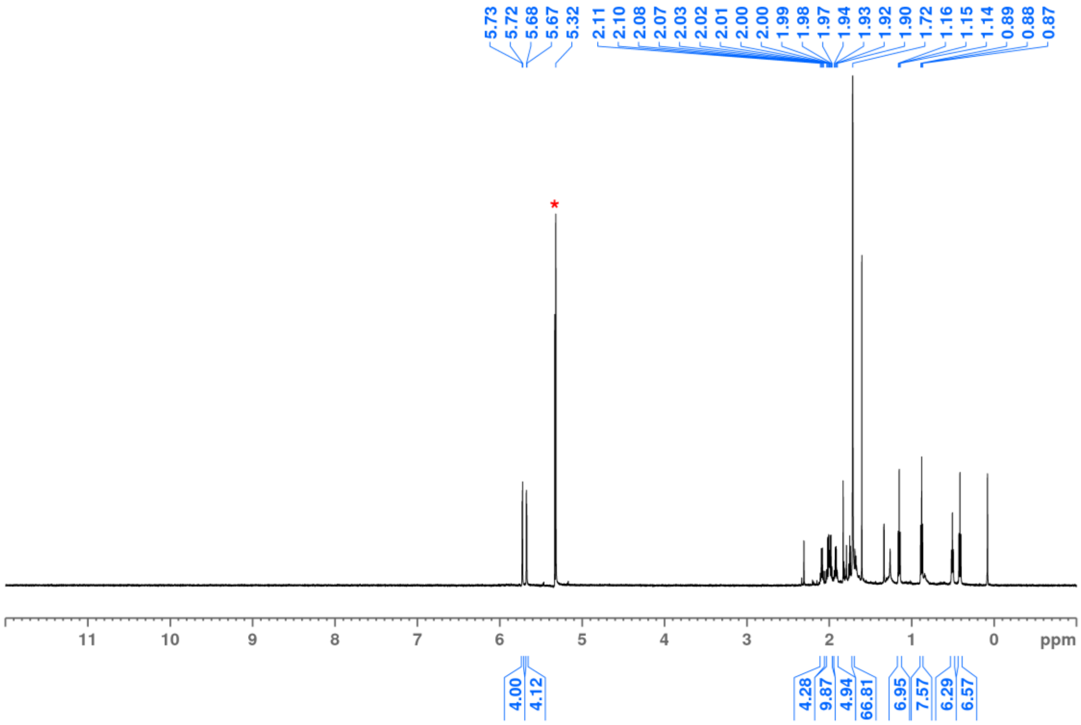

Figure S 16: ^{1}H NMR spectrum (DCM-d_2, 600 MHz, 295 K) of **2** and a slight excess of decamethylcobaltocene in DCM-d_2 at room temperature showing the selective formation of the chlorido silicate [$CoCp_2$*][**1**].

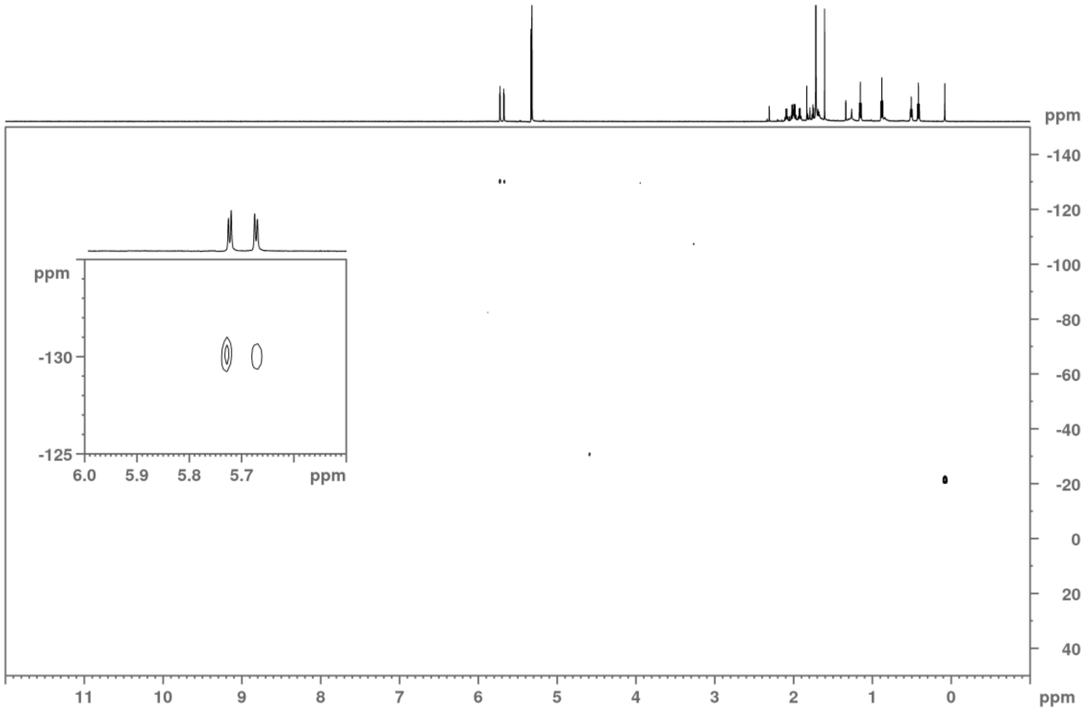

Figure S 17: ^{29}Si HMBC (DCM-d_2, 600 MHz, 295 K) spectrum of [$CoCp_2$*][**1**] formed by the reduction of **2** with decamethylcobaltocene in DCM-d_2.

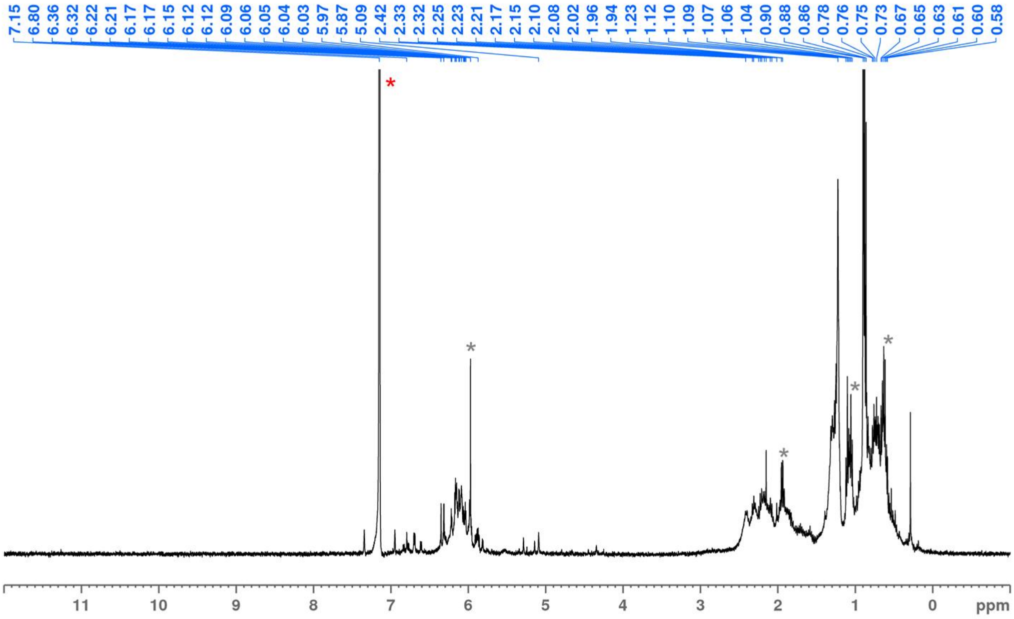

Figure S 18: ^{1}H NMR (C_6D_6, 600 MHz, 295 K) spectrum of **2** and slight shortage of decamethylcobaltocene in C_6D_6 at room temperature showing the successful reduction and formation of various unidetified products.Gray asterisks mark residual **2**.

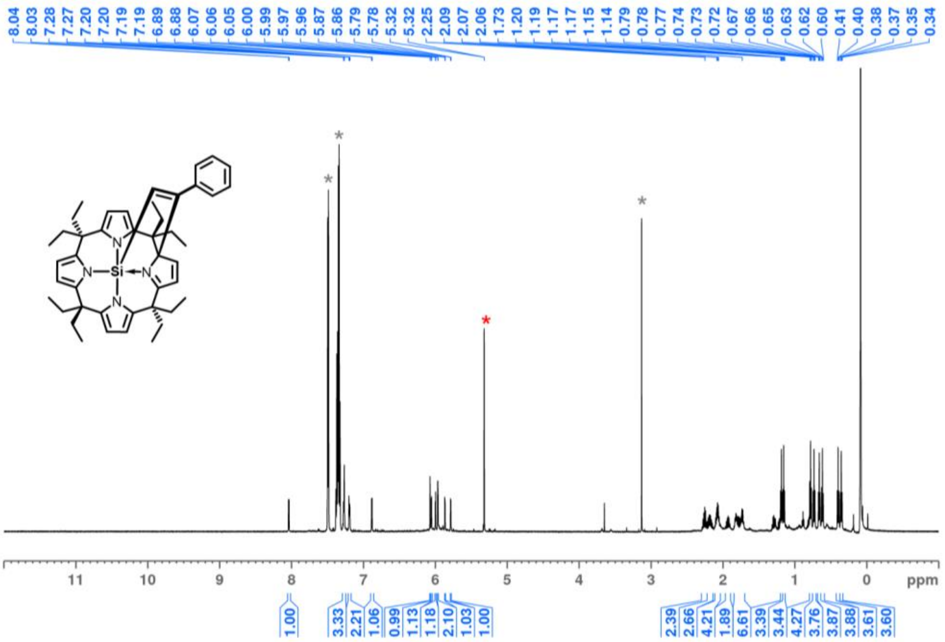

Figure S 19: ^{1}H NMR (DCM-d_2, 600 MHz, 295 K) spectrum of the phenylacetylene activation product by the planar silane **2**. Gray asterisks mark some excess of substrate.

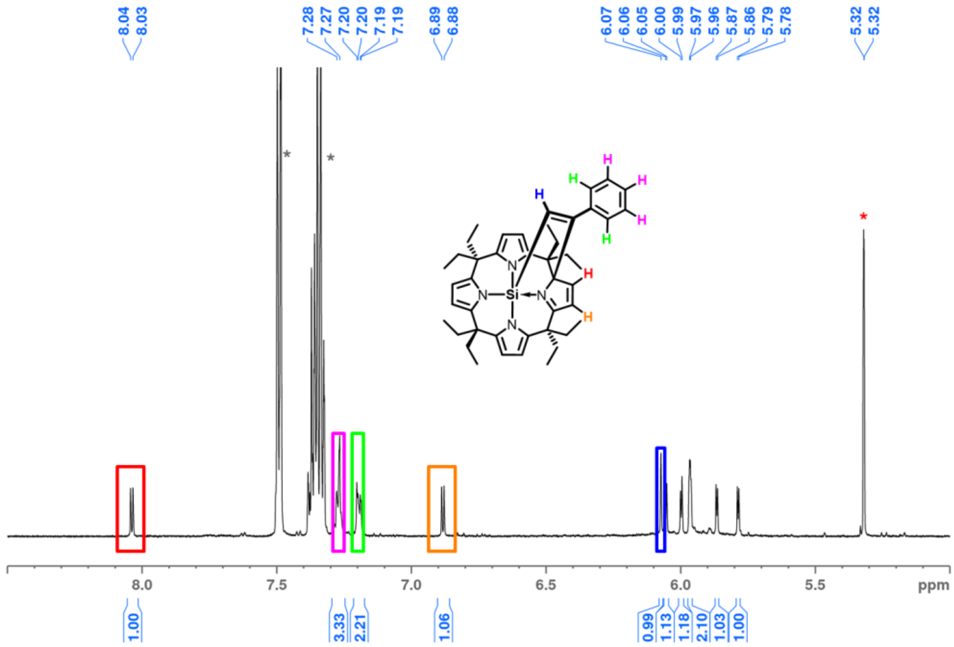

Figure S 20: Zoomed in area of the ^{1}H NMR (DCM-d_2, 600 MHz, 295 K) spectrum of the phenylacetylene activation product by the planar silane **2** showing the characteristic aromatic area and characteristic proton signals indicated by colors. Gray asterisks mark some excess of substrate.

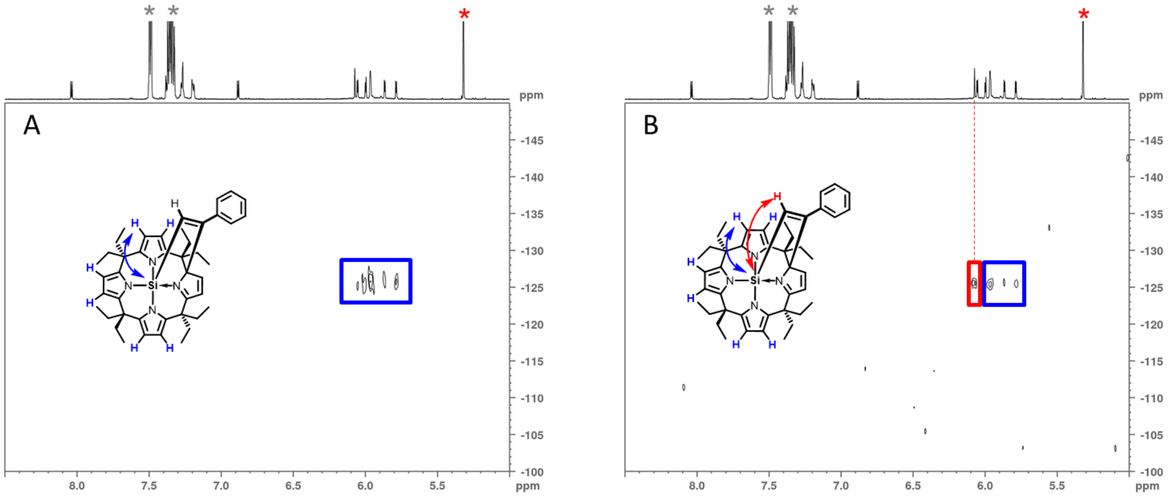

Figure S 21: ^{29}Si HMBC NMR (DCM-d_2, 600 MHz, 295 K) spectrum of the activation product of phenylacetylene by the planar silane **2**. The ^{29}Si shift of the activated species is δ = -126.0 ppm. A) Spectrum was measured with cnst13 = 3 Hz; B) Spectrum was measured with cnst13 = 7 Hz. Gray asterisks mark some excess of substrate.

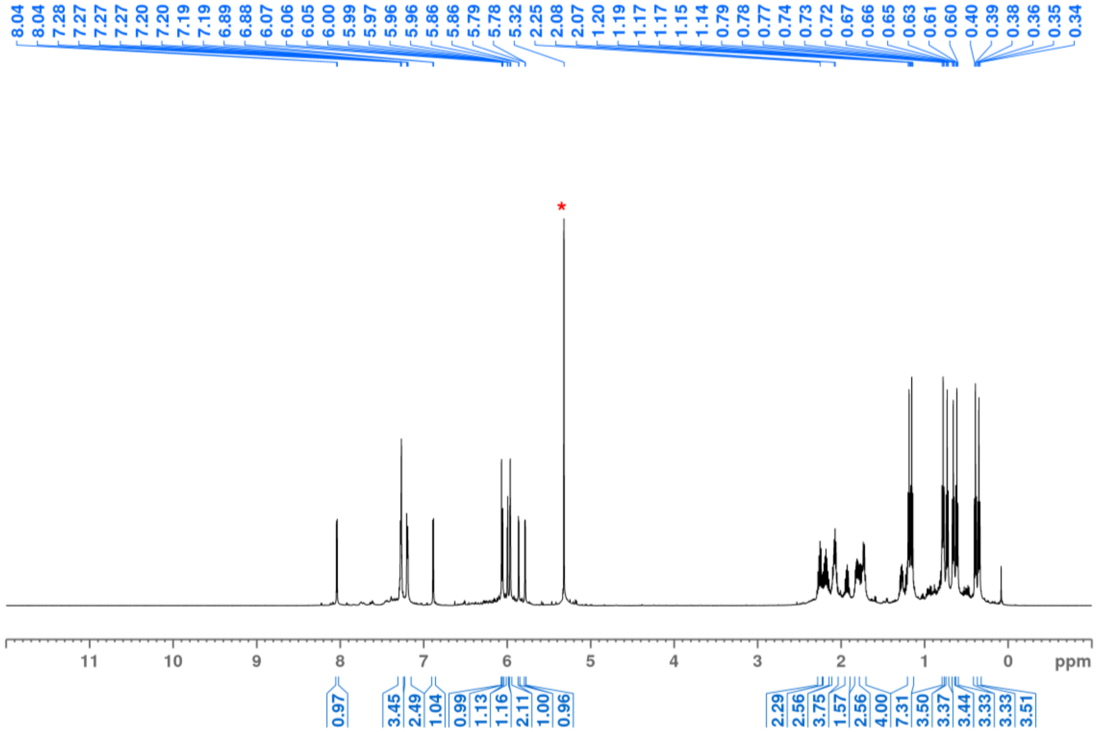

Figure S 22: ^{1}H NMR (DCM-d_2, 600 MHz, 295 K) spectrum of the isolated phenylacetylene activation product by the planar silane **2**.

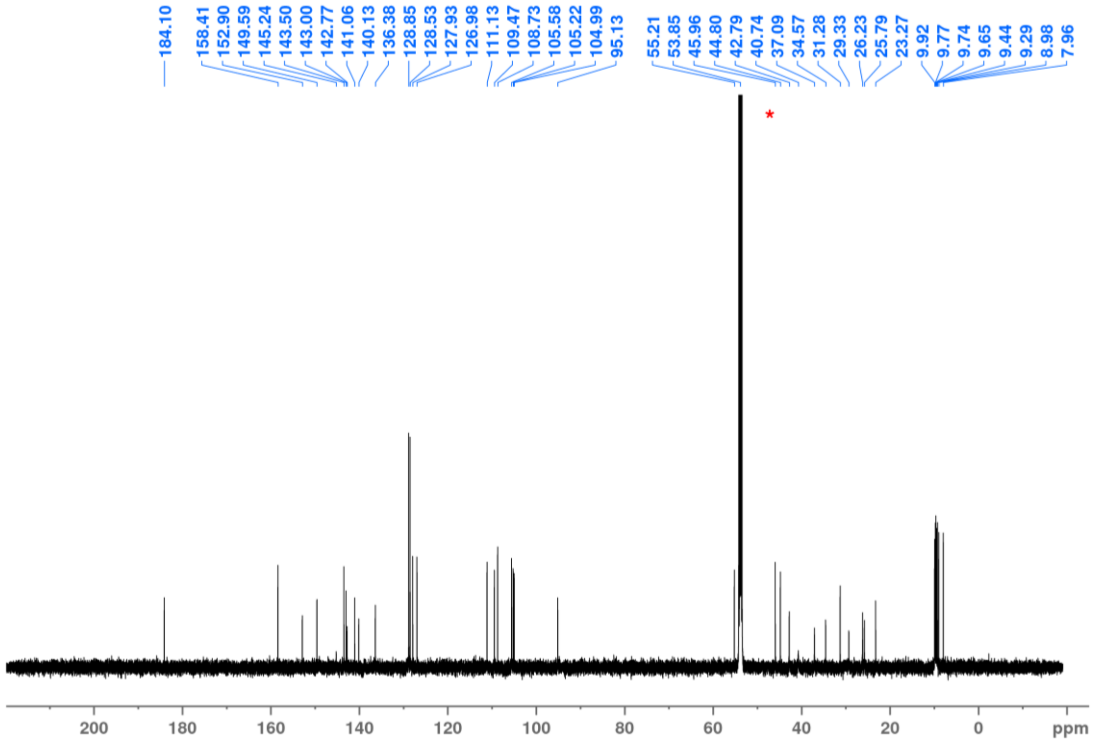

Figure S 23: ^{13}C NMR (DCM-d_2, 150 MHz, 295 K) spectrum of the isolated phenylacetylene activation product by the planar silane **2**.

Single crystal X-ray diffraction

Suitable crystals for single-crystal structure determination were taken directly from the mother liquor, covered with perfluorinated polyether oil and fixed on a cryo loop. Full shells of intensity data were collected at low temperature with a Bruker D8 Venture diffractometer, dual source (Mo-or Cu-K_α radiation, microfocus X-ray tube, Photon III detector). Data were processed with the standard Bruker (SAINT, APEX3) software package.[1, 2] Multiscan absorption correction was applied using the SADABS program.[3, 4] The structures were solved by intrinsic phasing[5, 6] and refined using the SHELXTL software package (Version 2018/3).[7-10] Graphical handling of the structural data during solution and refinement was performed with OLEX2.[11]

For the ShelXL SCRXD structure refinement of **2**, all hydrogen atoms were found in the residual density map and refined isotropically without restraints. All non-hydrogen atoms were given anisotropic displacement parameters.

The structure solution of **2** was further improved by Hirshfeld atom refinement,[12, 13] as implemented by the NoSphereA2 module in Olex2 version 1.3 alpha.[14] The method provides aspherical atomic electron densities created from the crystal-field embedded quantum-chemically derived electron density using the Hirshfeld partitioning. In the present case, the quantum chemically derived electron density was obtained at the PBE0/def2-TZVPP level of theory from ORCA. Coordinates of all atoms, including hydrogen atoms, were refined freely and anistropically. Hence, also for the hydrogen atoms, aspherical displacement parameters were obtained.

Crystallographic data for the structures reported in this article are deposited within the Cambridge Crystallographic Data Centre as supplementary publication no. CCDC 2042938-2042941 and can be obtained free of charge. For compound **2**, both the ShelXL refinement solution (CCDC 2042941) and the NoSphereA2 refinement solution ($\mathbf{2}_{\mathbf{SCXRD\text{-}HAR}}$, CCDC 2042939) were deposited. Crystal data and structure are summarized in the tables under the crystallographic figure.

Additional proof for the correct determination of the hydrogen atom positions via SCXRD/NoSpherA2 HAR method was obtained by DFT-theoretical reoptimization of the H-atom positions with PBEh-3c (see computational section for further details), starting from the $\mathbf{2}_{\mathbf{SCXRD\text{-}HAR}}$ refined structure. A shifting of max 2 pm was observed for the protons, as shown in table S1, which compares C-H bond lengths for the methylene groups.

Table S 1: Comparison of C-H bond lengths [pm] of **2** obtained by SCXRD and subsequent HAR with NoSpherA2 (PBE0/def2-TZVPP) and the “ab initio” results computed by PBEh-3c starting from the conventionally refined SCXRD structure.

	SCXRD-HAR	PBEh-3c	deviation
C27-H27b (in)	110.9	109.70	-1.2
C27-H27a (out)	109.8	109.30	-0.5
C35-H35b (in)	110.0	109.40	-0.6
C35-H35b (out)	110.1	109.40	-0.7
C29-H29b (in)	112.1	109.80	-2.3
C29-H29a (out)	108.1	109.20	1.1
C21-H21b (in)	110.2	109.30	-0.9
C21-H21b (out)	111.3	109.30	-2.0

Figures of molecular structures

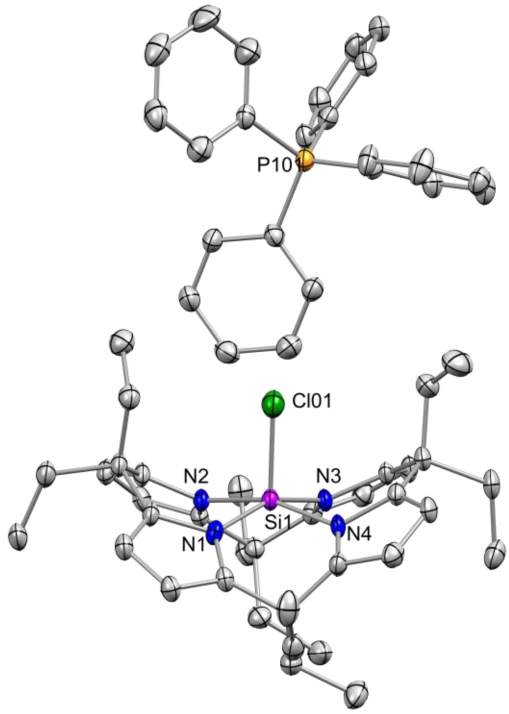

Figure S 24: Molecular structure of [**1**][PPh_4]. Hydrogen atoms are omitted for clarity. Displacement ellipsoids are drawn with a probability of 50 %. Selected bond distances (pm): Si(1)-N(1) 187.0(3), Si(1)-N(2) 186.6(3), Si(1)-N(3) 185.5(3), Si(1)-N(4) 185.0(3), Si(1)-Cl(01) 206.82(13). Selected bond angles (deg): N(1)-Si(1)-N(3) 157.23(13), N(2)-Si(1)-N(4) 161.52(13).

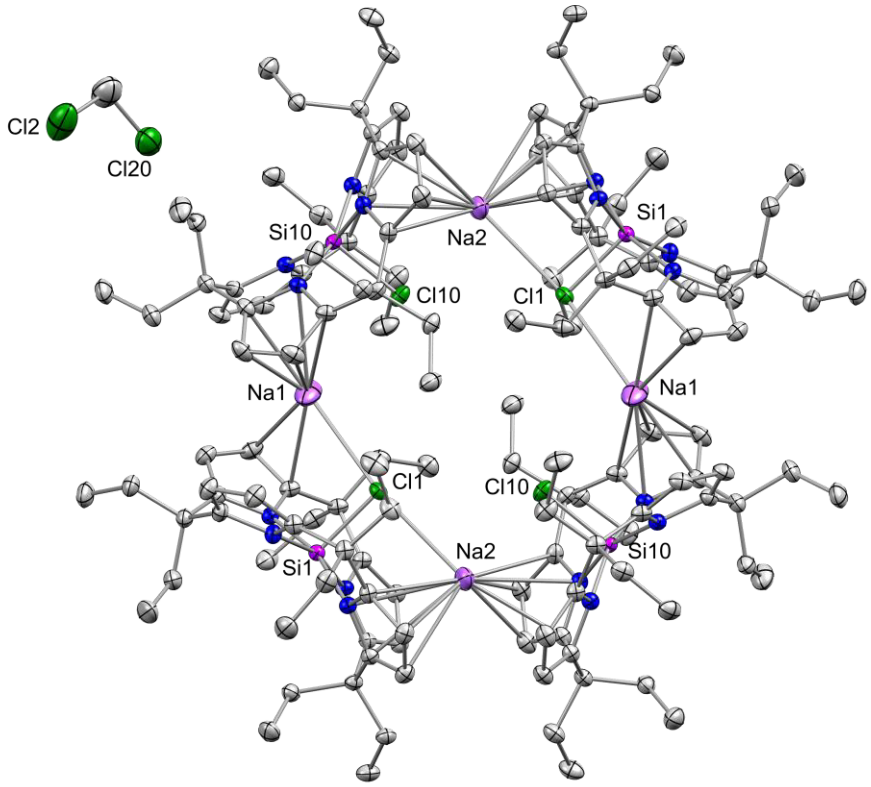

Figure S 25: Molecular structure of [**1**]$_4$[Na]$_4$. Hydrogen atoms are omitted for clarity. Displacement ellipsoids are drawn with a probability of 50 %. Two cell units are shown. For structural parameter see Figure S 26.

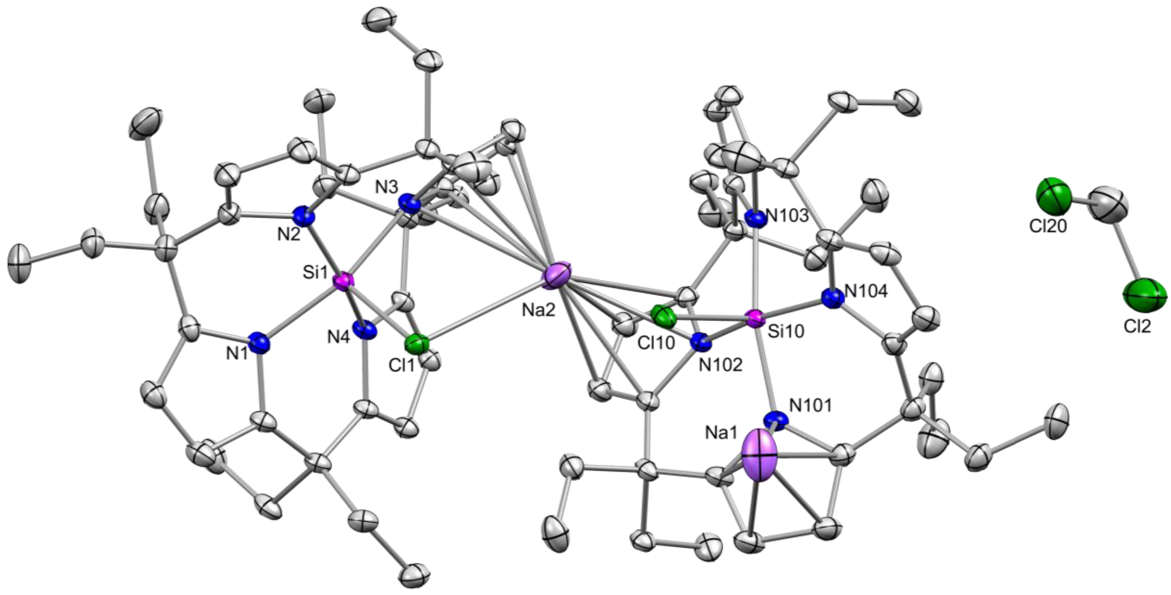

Figure S 26: Part of the molecular structure of [**1**]$_4$[Na]$_4$. Hydrogen atoms are omitted for clarity. Displacement ellipsoids are drawn with a probability of 50 %. Selected bond distances (pm): Si(1)-N(1) 186.15(13), Si(1)-N(2) 183.89(13), Si(1)-N(3) 186.53(13), Si(1)-N(4) 184.85(14), Si(1)-Cl(1) 213.47(6), Na(2)-Cl(1) 309.18(10), Si(10)-N(101) 185.72(13), Si(10)-N(102) 185.95(13), Si(10)-N(103) 182.73(13), Si(10)-N(104) 183.12(13), Si(10)-Cl(10) 213.5(2), Na(1)-Cl(10) 335.0(4). Selected bond angles (deg): N(1)-Si(1)-N(3) 163.40(6), N(2)-Si(1)-N(4) 162.38(7), N(101)-Si(1)-N(103) 163.58(6), N(102)-Si(1)-N(104) 162.13(6).

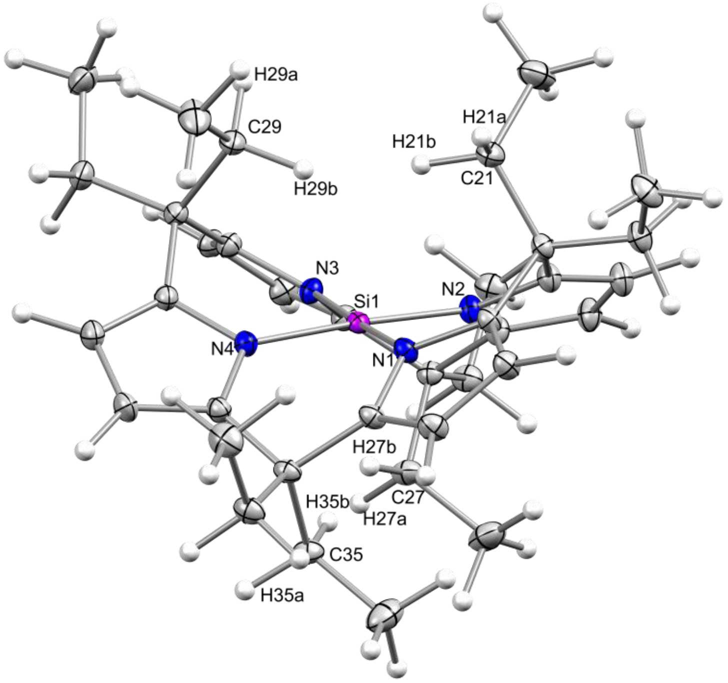

Figure S 27: Molecular structure of **2** (HAR). Displacement ellipsoids are drawn with a probability of 50 %. Selected bond distances (pm): Si(1)-N(1) 178.84(5), Si(1)-N(2) 179.26(5), Si(1)-N(3) 179.90(4), Si(1)-N(4) 178.81(4), C(29)-H(29a) 108.1, C(29)-H(29b) 112.1, C(21)-H(21a) 111.2, C(21)-H(21b) 110.2, C(27)-H(27a) 109.8, C(27)-H(27b) 111.0, C(35)-H(35a) 110.1, C(35)-H(35b) 109.9. Selected bond angles (deg): N(1)-Si(1)-N(3) 178.19(2), N(2)-Si(1)-N(4) 176.08(2), N(1)-Si(1)-N(2) 90.15(2), N(2)-Si(1)-N(3) 89.28(2), N(3)-Si(1)-N(4) 90.43(2), N(4)-Si(1)-N(1) 90.26(2).

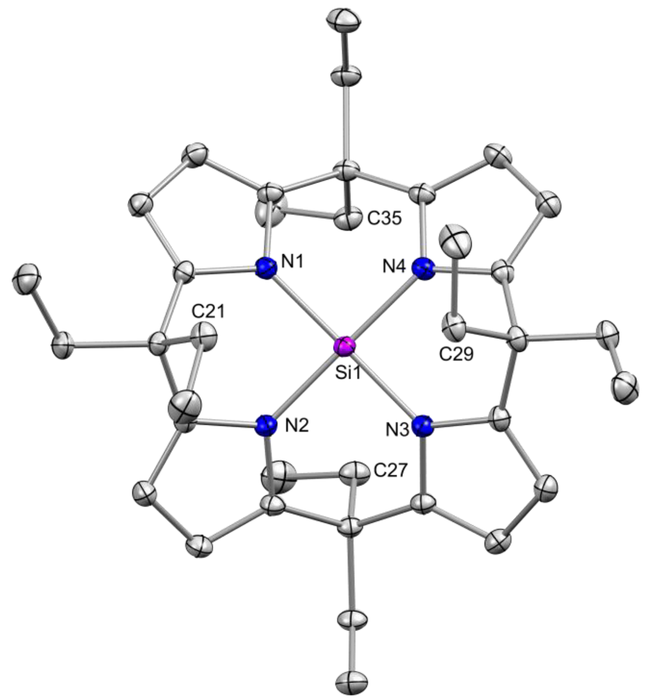

Figure S 28: Top-down view of molecular structure of **2**. Hydrogen atoms are omitted for clarity. Displacement ellipsoids are drawn with a probability of 50 %. For structural parameter see Figure S 27.

Table S 2: Crystallographic data

Compound	[PPh_4][**1**]	[Na]$_4$[**1**]$_4$	**2** (refined with ShelXL)	**2** (refined with NoSphereA2)
Identification code	mo_fe744_0m	mo_fe754_01	mo_fe736_0m	mo_fe736_0m_NoSpherA2
Empirical formula	$C_{60}H_{68}ClN_4PSi$	$C_{73}H_{98}Cl_4N_8Na_2Si_2$	$C_{36}H_{48}N_4Si$	$C_{36}H_{48}N_4Si$
Formula weight	939.69	1331.55	564.87	564.895
Temperature/K	100.0	100.0	100.0	100.0
Crystal system	triclinic	triclinic	monoclinic	monoclinic
Space group	P-1	P-1	$P2_1/n$	$P2_1/n$
a/Å	12.0514(9)	14.099(2)	10.3052(4)	10.3052(4)
b/Å	13.9832(10)	15.651(2)	19.3263(7)	19.3263(7)
c/Å	16.6437(13)	16.334(3)	14.9876(7)	14.9876(7)
α/°	78.997(3)	77.122(6)	90	90
β/°	73.403(3)	84.014(6)	94.396(2)	94.396(2)
γ/°	69.275(3)	76.639(6)	90	90
Volume/Å^3	2501.3(3)	3413.1(9)	2976.2(2)	2976.2(2)
Z	2	2	4	4
ρ_{calc}g/cm^3	1.248	1.296	1.261	1.261
μ/mm^{-1}	0.177	0.271	0.112	0.112
F(000)	1004.0	1420.0	1224.0	1224.7
Crystal size/mm^3	0.135 × 0.116 × 0.102	0.12 × 0.103 × 0.095	0.191 × 0.189 × 0.184	0.191 × 0.189 × 0.184
Radiation	MoKα (λ = 0.71073)	MoKα (λ = 0.71073)	MoKα (λ = 0.71073)	Mo Kα (λ = 0.71073)
2Θ range for data	3.72 to 53.996	3.816 to 57.998	4.636 to 61.09	4.64 to 61.1
Index ranges	-15 ≤ h ≤ 15, -17 ≤ k ≤ 17, -	-19 ≤ h ≤ 19, -21 ≤ k ≤ 21, -22	-14 ≤ h ≤ 14, -27 ≤ k ≤ 27, -21 ≤ l	-14 ≤ h ≤ 14, -27 ≤ k ≤ 27, -21 ≤ l
Reflections collected	75362	201842	113758	113758
Independent reflections	10910 [R_{int} = 0.1784,	18139 [R_{int} = 0.0464, R_{sigma} =	9086 [R_{int} = 0.0505, R_{sigma} =	9082 [R_{int} = 0.0505, R_{sigma} =
Data/restraints/parameter	10910/0/612	18139/0/828	9086/0/406	9082/0/802
Goodness-of-fit on F^2	1.017	1.039	1.051	1.089
Final R indexes [I>=2σ (I)]	R_1 = 0.0650, wR_2 = 0.1338	R_1 = 0.04446, wR_2 = 0.1131	R_1 = 0.0380, wR_2 = 0.0979	R_1 = 0.0203, wR_2 = 0.0399
Final R indexes [all data]	R_1 = 0.1321, wR_2 = 0.1679	R_1 = 0.0528, wR_2 = 0.1195	R_1 = 0.0459, wR_2 = 0.1046	R_1 = 0.0280, wR_2 = 0.0436
Largest diff. peak/hole / e	0.52/-0.64	1.00/-0.76	0.42/-0.29	0.24/-0.16
CCDC Database number	2042938	2042940	2042941	2042939

ATR-IR spectra

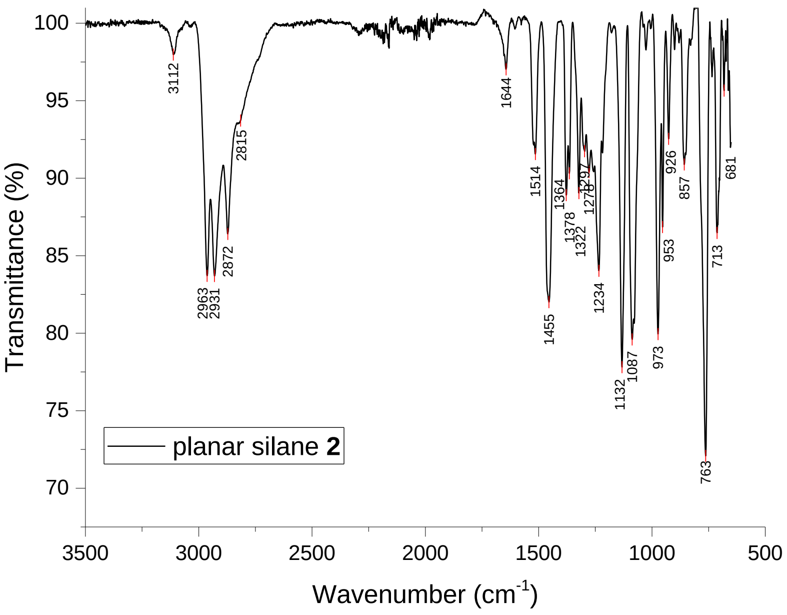

Figure S 29: IR spectrum of **2**.

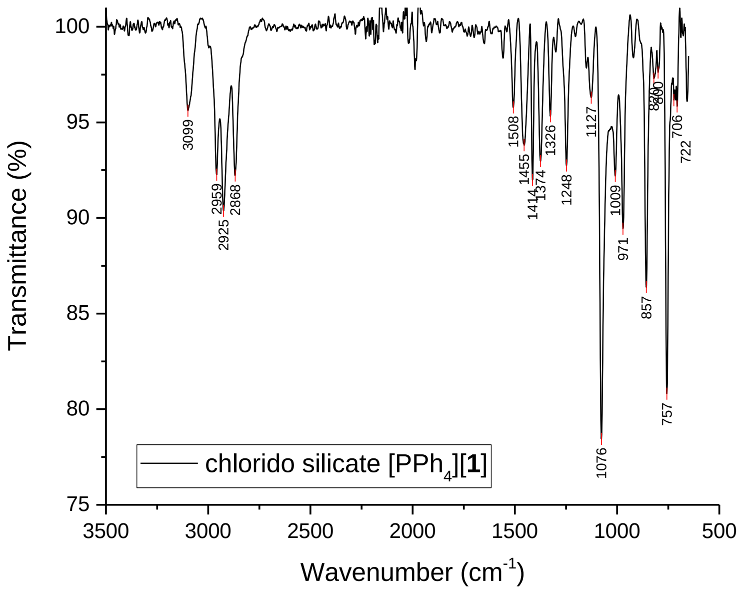

Figure S 30: IR spectrum of [PPh$_4$][**1**].

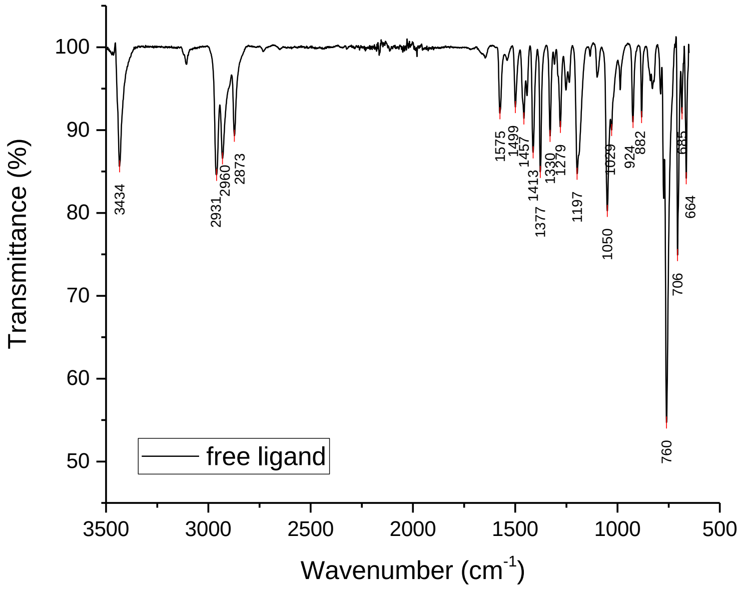

Figure S 31: IR spectrum of free *meso*-Octaethylcalix[4]pyrrole ligand as reference.

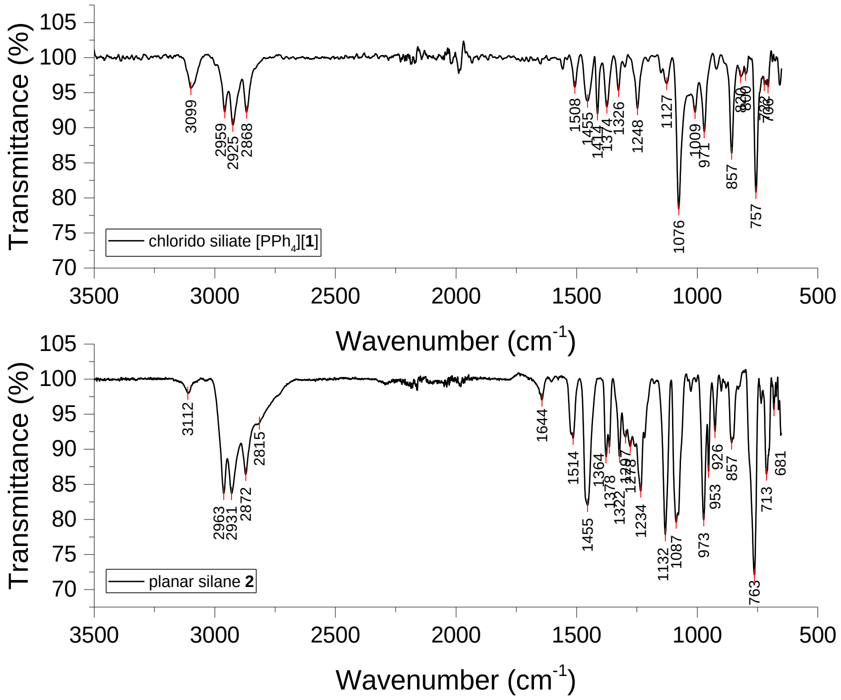

Figure S 32: Comparison of the IR spectra of [PPh4][**1**] and **2**. The shoulder at 2815 cm^{-1} indicates the agostic interaction in the planar silane **2**.

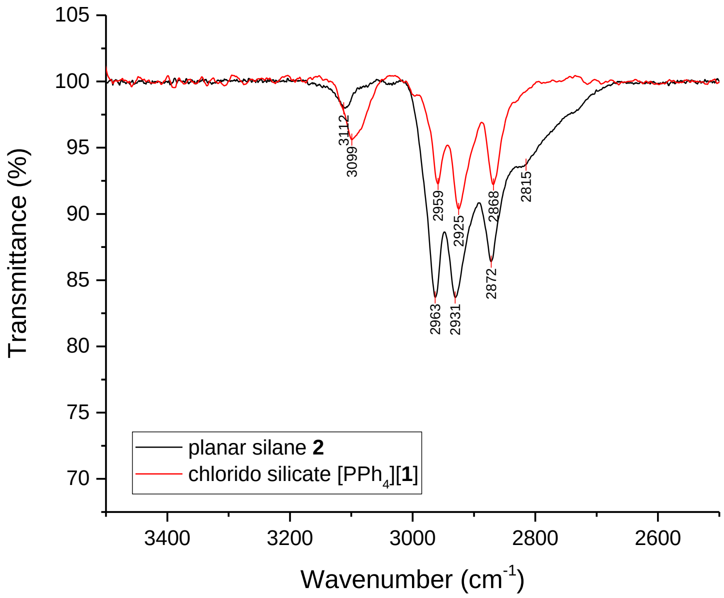

Figure S 33: Zoom of Figure S 32 from 3500 – 2500 cm^{-1} showing the band at 2815 cm^{-1} assigned to the agostic interactions in the planar silane **2**.

UV-Vis spectra

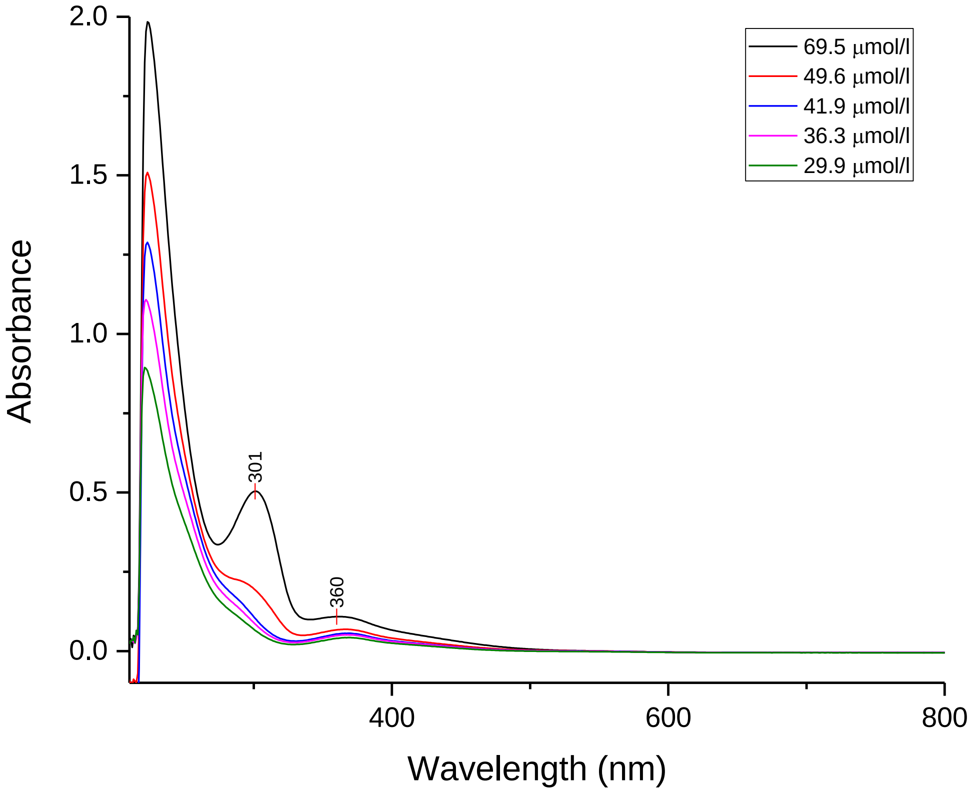

Figure S 34: UV-Vis spectrum of **2** in DCM with d = 0.2 cm.

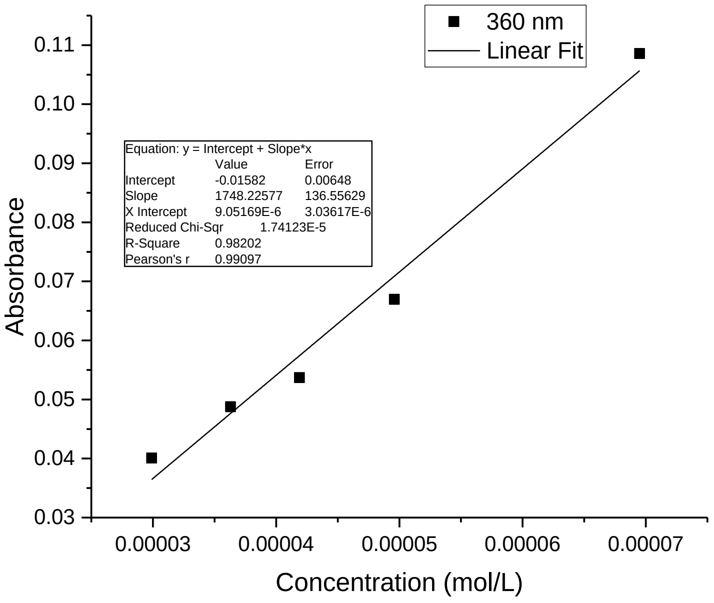

Figure S 35: Determination of the extinction coefficient of **2** from data of Figure S 34: ε(360 nm) = 8740 ± 680 L mol^{-1} cm^{-1}.

CV measurements

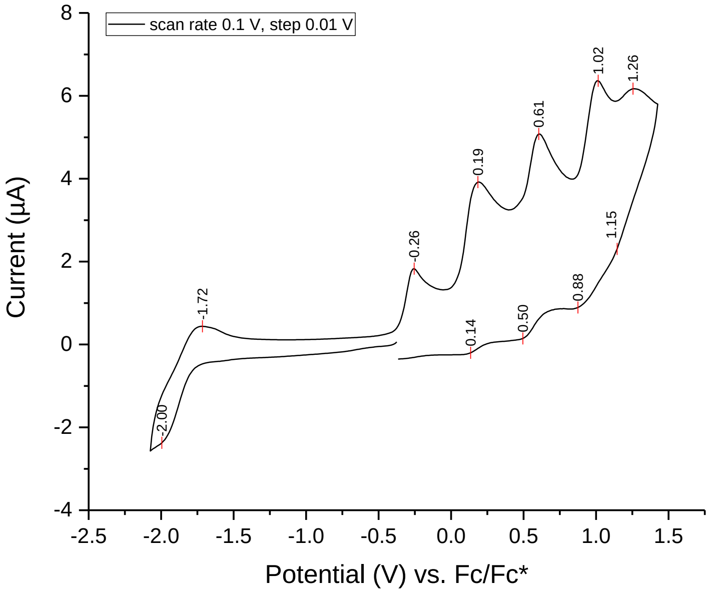

Figure S 36: CV experiment of **2** (1 mM) in DCM with 0.1 V/s NBu_4BArF_{20} (0.1 M).

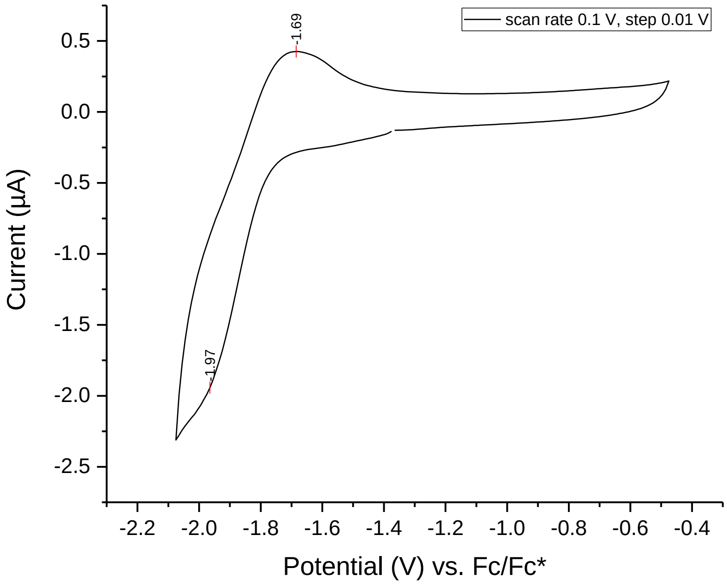

Figure S 37: CV experiment of **2** (1 mM) in DCM with 0.1 V/s NBu_4BArF_{20} (0.1 M).

Supplemental Computational Data

For all quantum chemical calculations, Orca 4.1.2 or 4.2.1[15] was employed. Ball and stick representations were rendered with Chemcraft 1.8 or IBOView.[16]

Structure optimization

All structures were optimized with the PBEh-3c model[17] and confirmed to possess only positive Hessian matrix eigenvalues. PBEh-3c is a composite electronic structure method based on a hybrid Perdew-Burke-Ernzerhoff (PBE) exchange-correlation functional combined with polarized valence-double zeta Gaussian atomic orbital basis sets (def2-mSVP). It treats basis set superposition errors with the geometrical counterpoise scheme (gCP)[18], and London dispersion interactions with the Becke-Johnson-damped D3 correction[19, 20]. PBEh-3c produces results of similar structural accuracy to those obtained with MP2/def2-TZVPP.[21] Comparison of selected computed bond lengths with the SCXRD derived data of **2** and **3** is presented in Table S 3. It illustrates the very good performance of the computational model, with a maximum bond length deviation of 1.7 pm and bond angle deviation of 1° for **2** and **3** (Si(pyrrole)$_4$).

Table S 3: Comparison of PBEh-3c computed metric parameters with SCXRD-HAR derived values of **2** and the literature SCXRD values of **3**.[22]

bond length [pm]	**PBEh-3c**	**SCXRD**	**Deviation**
bond angles (°)			
**[2]**			
Si-N1	180.11	178.85	1.3
Si-N2	179.93	179.26	0.7
Si-N3	180.12	179.90	0.2
Si-N4	179.89	178.82	1.1
N1-C1	139.43	141.11	-1.7
C1-C2	135.99	136.49	-0.5
N1-Si-N3	177.0	178.2	-1.2
N1-Si-N2	175.3	176.1	-0.8
**[3]**		**SCXRD[22]**	
Si-N1	173.62	172.50	1.12
N1-C1	138.10	139.30	-1.20
C1-C2	136.10	135.70	0.40
N1-Si-N2	111.0	110.5	0.50

Figure S 38 shows the computed PBEh-3c minimum structure of **2**, with (unscaled) IR stretching frequencies of the assigned C-H bonds (major contributions). Although the values are systematically shifted compared to the experimental data, they show the same trend → the smallest for the “agostic” C-H-bonds (endo-in).

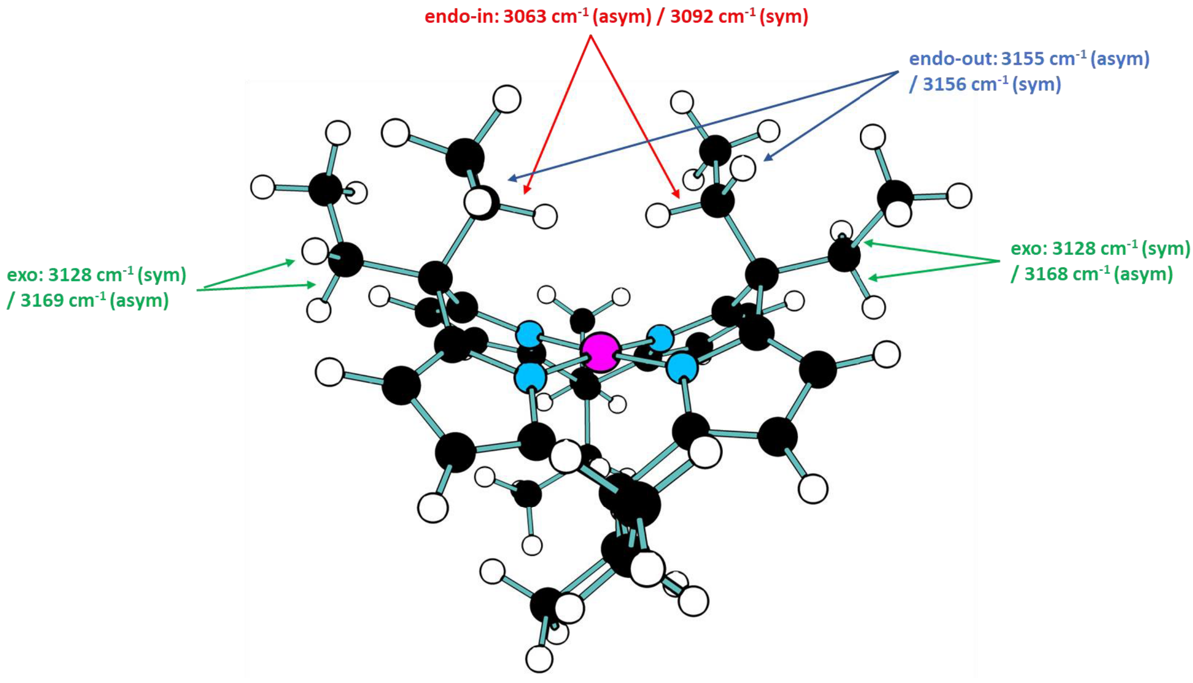

Figure S 38: Computed PBEh-3c minimum structure of **2** and the computed vibrational frequencies.

Single point computation for electron affinities and frontier molecular energies

The gas phase single point computations for the discussion of frontier molecular orbital energies and electron affinities were calculated with the hybrid meta exchange-correlation functional PW6B95-D3(BJ), that was shown to be very reliable for the computation of electron affinities,[23] in combination with the extensive def2-QZVPP basis set.[24] SCF settings were tightSCF and integration was performed at a very fine grid7. The RIJCOSX Fock-matrix formation algorithm was used as implemented in Orca along with the respective automatically generated auxiliary basis sets (AutoAux)[25]. The RIJCOSX scheme combines the chain of spheres exchange approximation (COSX)[26] for the computation of the exchange matrix with the Split-RI-J algorithm[27] for the calculation of the Coulomb matrix. Enthalpies at 298.15 K were calculated with the total thermal and zero-point energy correction from the PBEh-3c calculation combined with the electronic single point energies obtained on the PW6B95-D3(BJ)/def2-QZVPP level of theory.

Table S 4: PBEh-3c derived enthalpy correction and PW6B95-D3(BJ)/def2-QZVPP derived single point energy, enthalpy and FMO energies.

	**Thermal + ZPE correction from PBEh-3c [kJ mol^{-1}]**	**E [Hartree] PW6B95-D3(BJ)/def2-QZVPP**	**E [kJ mol^{-1}]**	**H [kJ mol^{-1}]**	HOMO [eV]	LUMO [eV]
**2**	2199.9	-1912.4490	-5021134.8	-5018935.0	-5.69	-2.3
**2_radical_anion**	2195.3	-1912.5102	-5021295.6	-5019100.3		
**3**	842.6	-1129.6732	-2965956.9	-2965114.3	-6.58	0.14
**3_radical anion**	836.4	-1129.6729	-2965956.1	-2965119.8		

			E_{rad_anion} – $E_{neutral}$	H_{rad_anion} – $H_{neutral}$		
	**Electron Affinity**	**for 2**	160.8	165.3		
		**for 3**	-0.8	5.5		

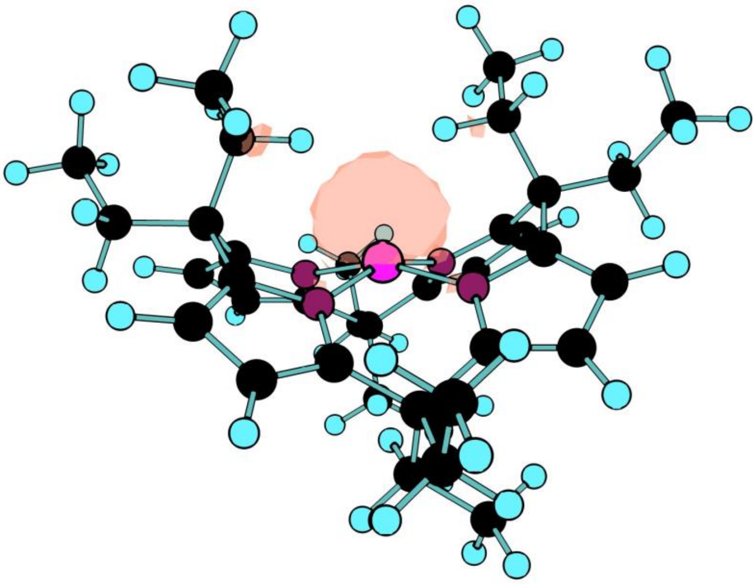

Figure S 39: Spin Density Distribution of **2_radical_anion** (PBEh-3c, isodensity value 0.005 au.

Electron density evaluation of the agostic interaction by NBO and QTAIM

Natural bond orbitals (NBO) [28, 29] or the quantum theory of atoms in molecules (QTAIM) [30] both analyze the electron density distribution. In recent work, it was shown that the hybrid PBE0 functional [31, 32] is among the best performing functionals for the accurate description of the electron density distribution, referenced against exact all-electron coupled-cluster singles and doubles densities.[33] Hence, the wavefunction for the gas-phase PBEh-3c optimized structure [$\mathbf{2}_{\mathbf{PBE\text{-}Gas}}$] was recomputed with PBE0/def2-TZVPP at fine grid6 settings, and analyzed with NBO 7.0 [34] and AIMAll Version 19.10.12.[35] Besides, the wavefunction for the molecular structure obtained by SCXRD + Hirshfeld Atom Refinement, named [$\mathbf{2}_{\mathbf{SCXRD\text{-}HAR}}$], was computed at PBE0/def2-TZVPP and analyzed accordingly. The NBO results of relevance for the description of the agostic interaction can be found in Table S 5.

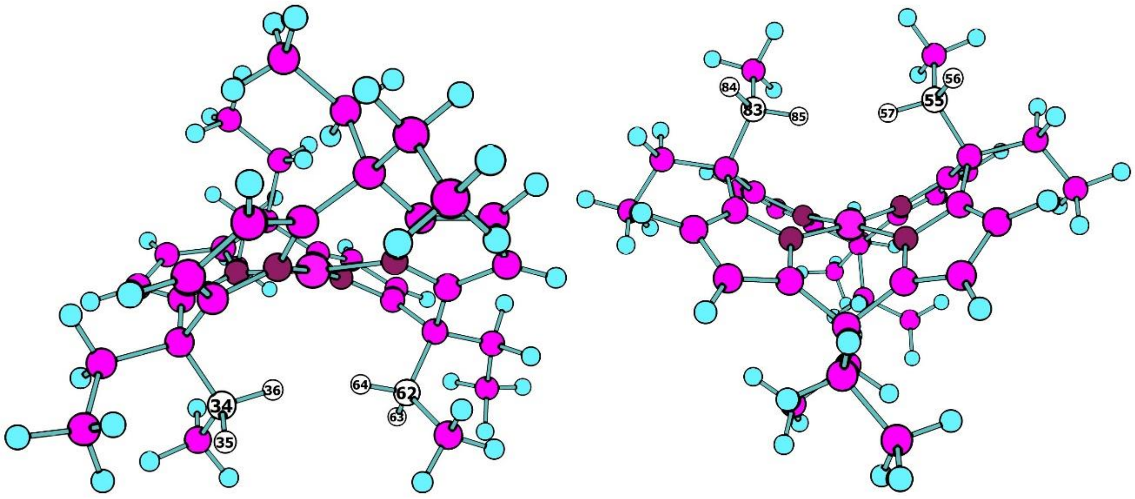

Table S 5: Relevant NBO parameters and metrical data for the gas-phase PBEh-3c optimized structure [$\mathbf{2}_{\text{PBE-Gas}}$] and the SCXRD-HAR derived experimental structure [$\mathbf{2}_{\text{SCXRD-HAR}}$], obtained from the PBE0/def2-TZVPP electron density. For the "inward, agostic" methylene protons: **col1)** number of the considered CX-HY NBO, **col2)** H-Si distance, **col3)** occupation number of the C-H natural bond orbital, **col4)** second-order perturbation energy for the $\sigma_{C\text{-}H} \rightarrow Si(p_z)$

Col1	Col2	Col3	Col4
**NBO of (C X)-(H Y)**	**Si-H dist. [pm]**	**σ(C-H) NBO occ. num.**	**2nd ord. pert. [kcal mol^{-1}]**
[$\mathbf{2}_{\text{PBE-Gas}}$]			
C 34- H 35		1.976	
C 34- H 36	263.2	1.953	5.42
C 55- H 56		1.977	
C 55- H 57	283.4	1.962	3.29
C 62- H 63		1.976	
C 62- H 64	263.9	1.954	4.73
C 83- H 84		1.976	
C 83- H 85	282.7	1.961	3.35
[$\mathbf{2}_{\text{SCXRD-HAR}}$]			
C 34- H 35		1.976	
C 34- H 36	273.3	1.960	3.53
C 55- H 56		1.978	
C 55- H 57	258.6	1.950	6.73
C 62- H 63		1.976	
C 62- H 64	240.3	1.935	9.26
C 83- H 84		1.977	
C 83- H 85	296.3	1.967	1.91

As can be seen in the Table S 5, the shorter the Si-H bond distance (**col2**), the stronger is the $\sigma_{C\text{-}H}$ NBO depletion (**col3**), and the more considerable is the second-order perturbation energy (**col4**). The overall magnitude of NBO-derived parameters is reminiscent of those found for α-agostic interactions for titanium or chromium alkyl complexes.[36] A representative $\sigma_{C\text{-}H} \rightarrow Si(p_z)$ NBO pair is depicted in Figure S 40.

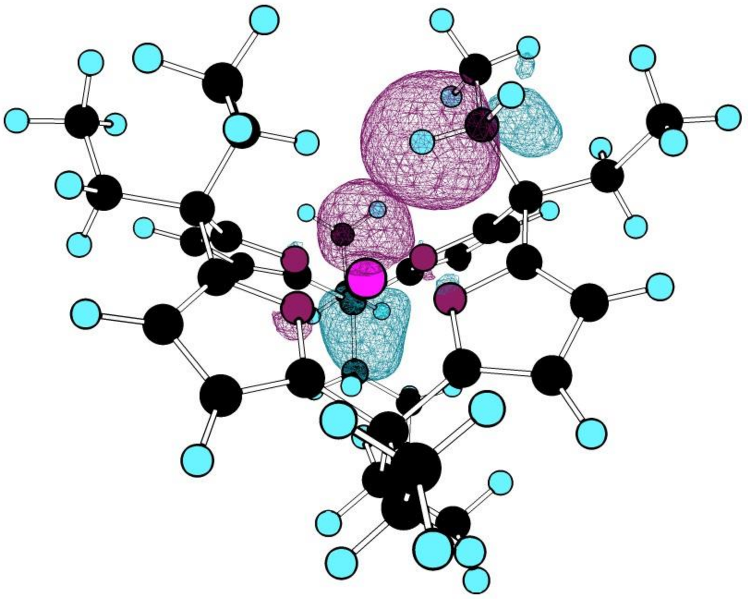

Figure S 40: A representative $\sigma_{C\text{-}H}$ → Si(p_z) NBO pair illustrating the donor-acceptor interaction in the [**2**$_{\mathbf{PBE\text{-}Gas}}$] structure

The Si-H distance (pure metric parameter) is in good correlation with $\sigma_{C\text{-}H}$ NBO occupation number (a parameter derived from the eigenfunctions of the one-particle density matrix) (Figure S 41). This observation is only to explain by a through-space orbital interaction. The (negative) hyperconjugative stabilization factor that occurs in d-block metal β-agostic bonds is absent in this case,[37] but alternative origins for this interaction, such as London dispersion, cannot be ruled out.[38, 39]

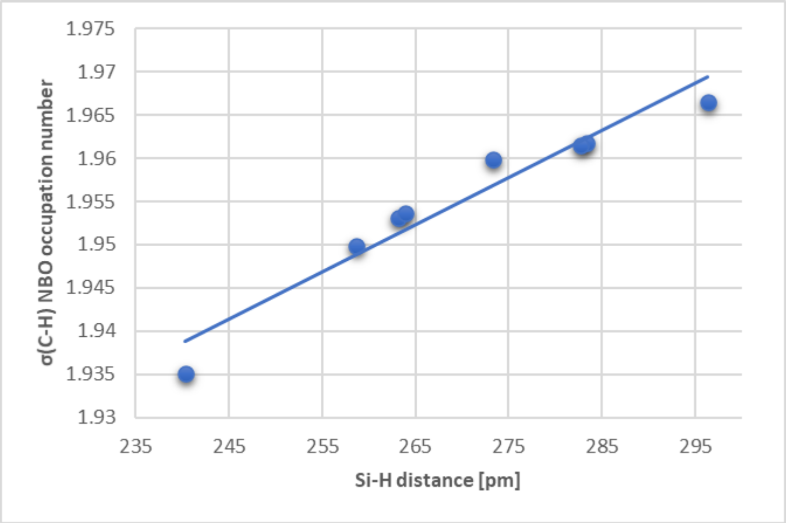

Figure S 41: Correlation of the σC-H NBO occupation number of the "interacting" methylene C-H bonds with the Si-H distance.

Further support for a C-H → Si interaction was provided by a bond critical point in the QTAIM topological analysis of the electron density distribution of the PBE0/def2-TZVPP wavefunction for [**2**$_{\mathbf{SCXRD\text{-}HAR}}$] (default settings in AIMALL). The respective molecular graph can be found in Figure S 42. It occurs between silicon and the closest H64 (see Table S 5). The bond critical point characteristic properties are in line with those reported for d-block metal agostic interactions.[40, 41] For the non-tilted methylene groups in the DFT-gas-phase optimized structure [**2**$_{\mathbf{PBE\text{-}Gas}}$], no bond critical point was found.

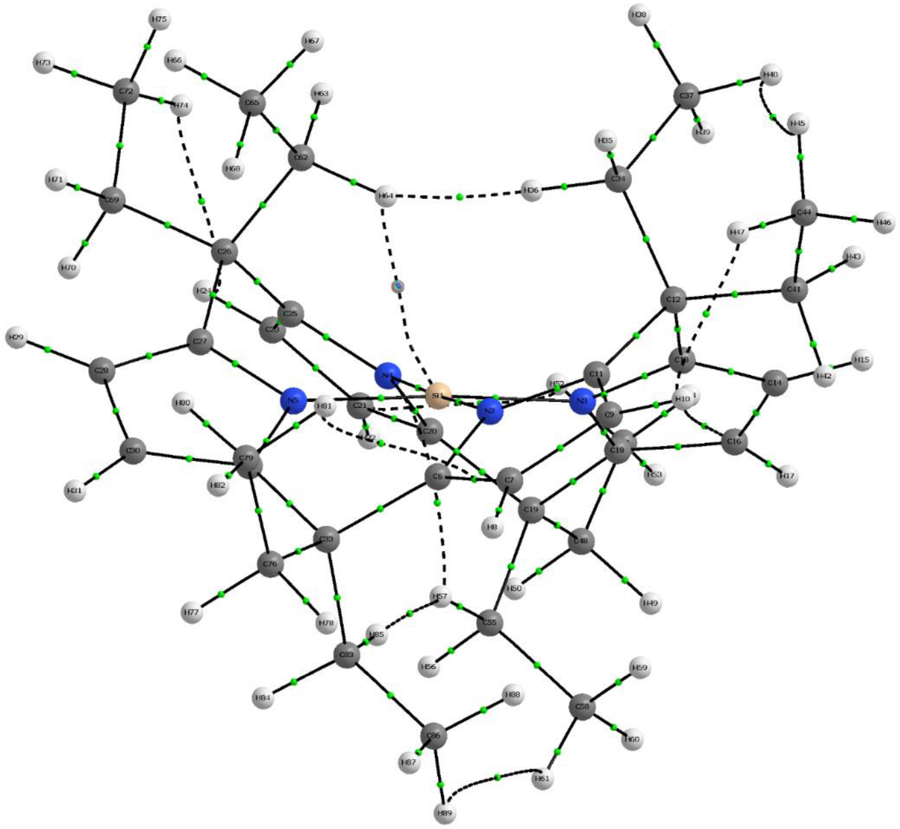

Figure S 42: Molecular graph obtain with AIMAll for the analysis of the PBE0/def2-TZVPP wavefunction of [$\mathbf{2}_{\mathrm{SCXRD\text{-}HAR}}$].

Table S 6: Second order perturbation energy between calix[4]pyrrol nitrogen atoms and silicon-centered acceptor orbitals, as obtained by NBO for the gas-phase PBEh-3c optimized structure [$\mathbf{2}_{\mathrm{PBE\text{-}Gas}}$] from the PBE0/def2-TZVPP electron density. The ligand and the silicon were considered as two individual fragments and the energies are shown for each Si-N bond. The natural electron configuration at silicon is s(0.57)p(1.05).

**Interaction**	N("sp2") (in-plane) → Si(3s-orbital)	N("sp2") (in-plane) → Si(3p_x-orbital)	N("sp2") (in-plane) → Si(3p_y-orbital)	N("p_z") (out-plane) → Si(3p_z-orbital)
**Second Order Perturbation Energy [kcal mol^{-1}] N1**	128	66	44	24
**N2**	127	43	68	26
**N3**	131	65	45	28
**N4**	130	41	68	30

Computational evaluation of the binding enthalpy of ethane to octa-protio calix[4]pyrrolato silane

To obtain an estimate of the “unrestricted” interaction energy of a hydrocarbon with a planar calix[4]pyrrolato silane, the model compound octa-protio calix[4]pyrrolato silane **4** and its ethane adduct were optimized at the highly accurate DLPNO-MP2/def2-TZVPP level of theory. The geometry optimization confirmed a planar structure of the free silane **4** and tightly bound ethane in the complex as the global minimum (distance CH-Si = 2.31 Å), and provided thermal correction parameters for the binding enthalpy. The final binding enthalpy was obtained by DLPNO-CCSD(T)/def2-QZVPP level of theory with the matching auxiliary basis sets and VeryTightSCF/tightPNO settings.[24, 42-45] Single-determinant dominated wavefunctions in the DLPNO-CCSD(T) computations were secured by evaluation of the T1 diagnostics (< 0.02).[46]

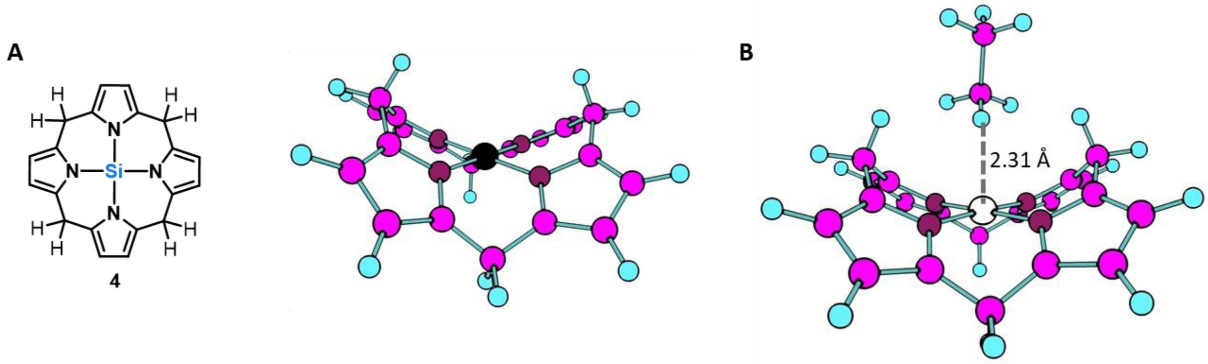

Figure S 43:**A** Lewis Structure and DLPNO-MP2/def2-TZVPP computed structure of model compound **4**. **B** DLPNO-MP2/def2-TZVPP computed structure of the ethane adduct of **4,** including the “agostic” Si-H bond distance.

Table S 7: Computed data for the ethane binding to octa-protio calix[4]pyrrolato silane **4**.

	**NIMAG**	**Enthalpy Correction [kJ mol^{-1}]**	**Entropy Correction T*S (298K) [kcal/mol^{-1}]**	**E DLPNO-CCSD(T)/def2-QZVPP [Hartree]**	**E [kJ mol^{-1}]**	**Enthalpy H [kJ mol^{-1}]**	**Gibbs free energy G [kJ mol^{-1}]**
**C_2H_6**	0	210.1	16.86	-79.6991	-209249.9	-209039.9	-209110.4
**HCxSi 4**	0	896.2	39.15	-1278.3795	-3356385.5	-3355489.3	-<br>3355653.2
**HCxSi_C_2H_6**	0	1110.6	45.37	-1358.0855	-3565653.5	-3564542.9	-<br>3564732.7
					**ΔE**	**ΔH**	**ΔG**
**C_2H_6-affinity**					**-18.0**	**-13.7**	**30.8**

Time-dependent density functional theory for computation of UV-Vis spectra for 2 and 3

The electronic transitions for **2** and **3** were computed by time-dependent density functional theory at the CAM-B3LYP/def2-TZVPP level of theory.[47] Obtained stick spectra of the first 20 electronic singlet transitions were convoluted with gaussian line-shape functions with a line width of 4000 nm. The computed spectrum and the comparison with the experimental spectrum of **2** and the computed spectrum of **3** can be found in Figure S 44. To account for the neglected solvation effects, all computed values were systematically shifted by +22 nm.

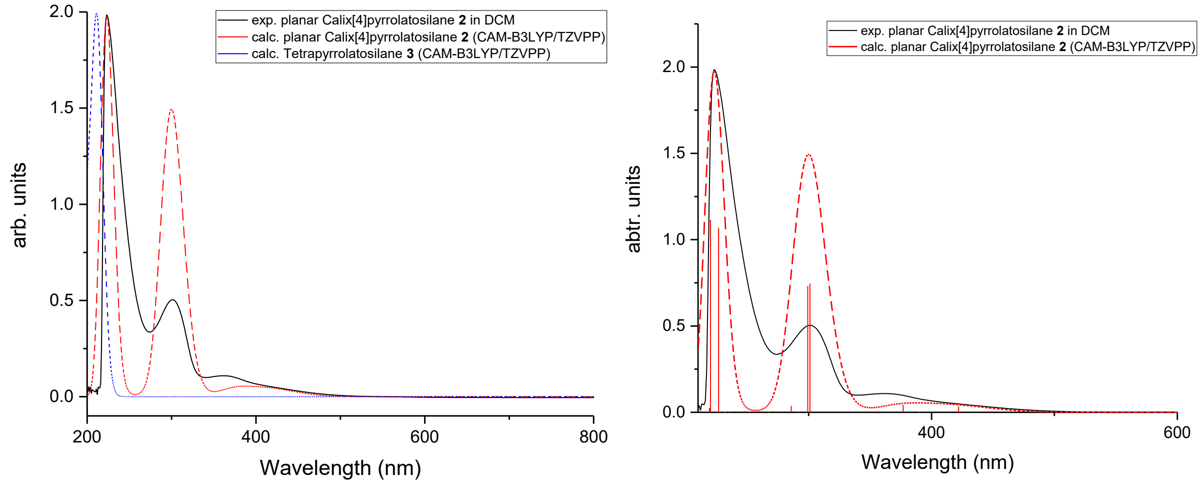

Figure S 44: Comparison of computed (CAM-B3LYP/def2-TZVPP) spectrum of **2** (red) and **3** (blue) and the experimental spectrum of **2** (black) in DCM.

To improve the qualitative description of the low energy electronic transitions, natural transition orbitals (NTO) were calculated.[48] The NTOs contributing to the four lowest energy transitions with significant oscillator strength can be found in Figure S 45. In all cases, the depicted pairs of NTOs cover participation >95%.

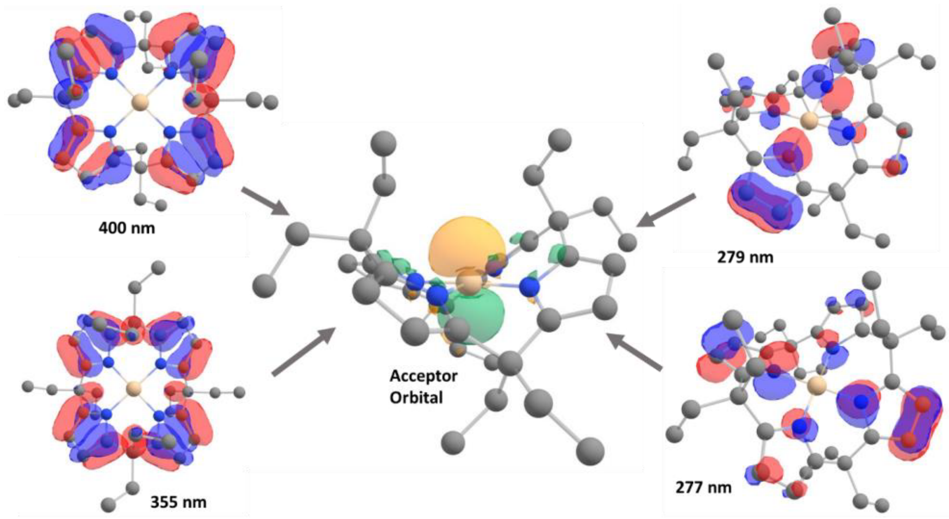

Figure S 45: Isodensity plot (0.06 au) for the involved natural transition orbitals for the electronic excitation at 400, 355, 279 and 277 nm.

As can be derived from the analysis of the NTOs, the lowest energy transitions at 400 and 355 nm occur by charge transfer of the anti-bonding and bonding combination of the local HOMOs of the pyrrole rings into the p_z-type orbital at silicon. The stronger transitions at 277 and 279 nm are of charge transfer of the HOMO-4 orbitals located at the pyrroles, which have a strong contribution of the pyrrole nitrogen electron lone-pairs, into the p_z-type orbital at silicon.

Computation of the three possible reaction pathways of 2 with phenylacetylene

The free energies for the transition states and final products for the two regioisomeric carbosilylation products (Prod_12 and Prod_21) between the model octamethyl-calix[4]pyrrolato silane and phenylacetylene, and the CH-activation product (Prod_CH) were obtained by at the PW6B95-D3(BJ)/def2-QZVPP//PBEh-3c/def2-mSVP level of theory. The corresponding reaction pathways are depicted in Figure S44. In agreement with the spectroscopic results, **Prod_12** is obtained as the kinetic product, whereas the thermodynamic **Prod_21** is not competing due to a high reaction barrier.

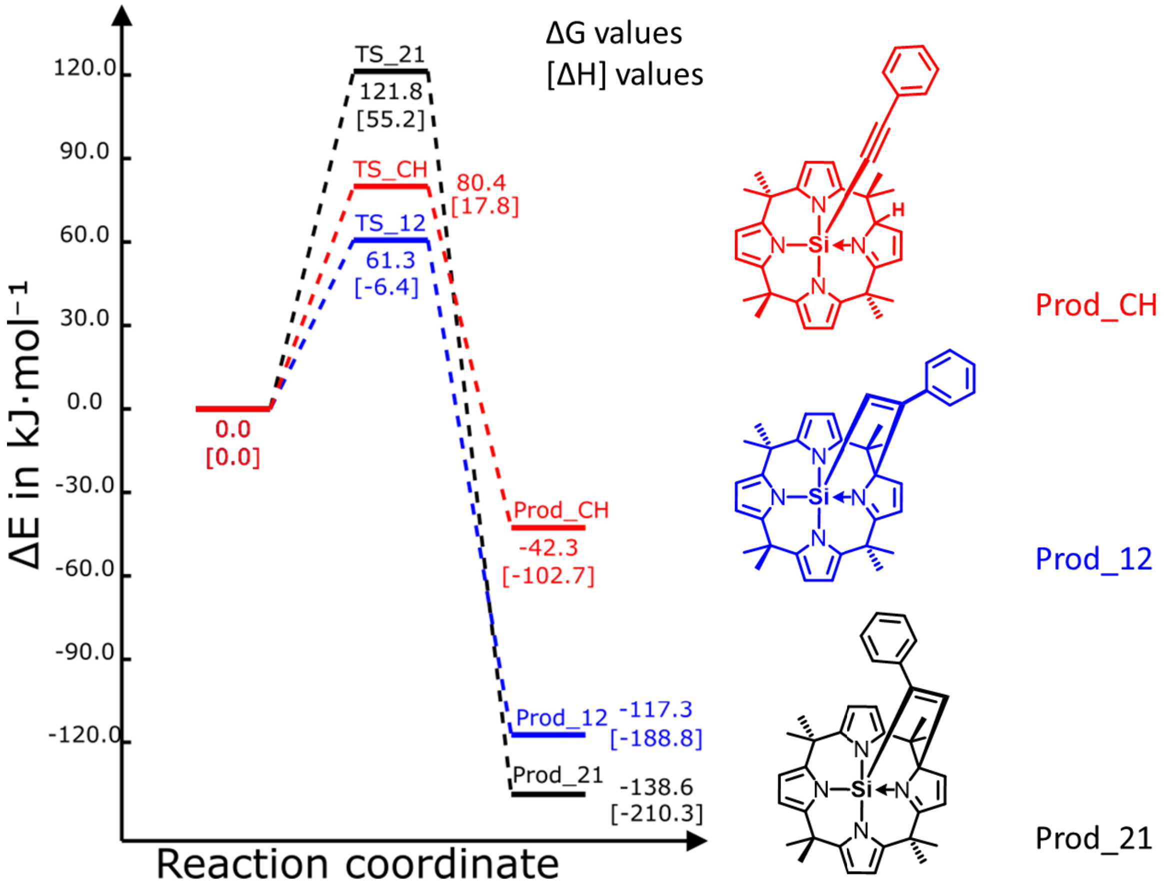

Figure S 46: Reaction pathways of the possible regio-isomeric products for the reaction between octamethyl-calix[4]pyrrolato silane and phenylacetylene. ΔG and [ΔH] values in kJ mol^{-1} obtained at PW6B95-D3(BJ)/def2-QZVPP//PBEh-3c/def2-mSVP.

Supplemental Experimental Procedures

Methods and Materials

Unless otherwise stated, all manipulations were carried out under a dry argon atmosphere using standard Schlenk techniques to prevent oxidation and hydrolysis of sensitive compounds. All solvents were rigorously dried using standard procedures, freshly degassed, and stored over molecular sieve (3 Å resp. 4 Å) before use. All glassware, syringes, magnetic stirring bars, and needles were thoroughly dried. The commercially available chemicals were used as received. All air-sensitive compounds were stored in a glove box (MBraun LABmaster dp, MB-20-G or Sylatech Glovebox) under N_2 atmosphere. The purity and identity of the compounds were confirmed by high-resolution multinuclear NMR spectroscopy, mass spectrometry, and, if possible, X-ray diffraction analysis. ^{1}H, ^{7}Li, ^{13}C{^{1}H} and ^{29}Si NMR spectra were collected with a Bruker DPX 200, Bruker Advance II 400, or Bruker 2 Advance III 600 NMR spectrometer and referenced to the solvent in use. Chemical shifts are reported as dimensionless δ values in ppm; coupling constants J are given in hertz (Hz). Electrospray ionization mass spectra were obtained with a Bruker ApexQe FT-ICR instrument. MALDI mass spectrometry was performed in a DFTB-Matrix on a Bruker AutoFlex Speed time-of-flight spectrometer. IR-Spectra was recorded on an Agilent Cary 630 spectrometer equipped with a diamond ATR unit processed with MicroLab PC and OriginPro 2017G 64Bit. *meso*-Octaethylcalix[4]pyrrole was prepared from 3-pentanone, pyrrole, and a catalytic amount of CH_3SO_3H in ethanol according to literature and purified via flash column chromatography.[49, 50] Deprotonation of the *meso*-octaethylcalix[4]pyrrole was performed in *n*-hexane, as reported in the literature.[51] Donor free Na[BArfF$_{20}$] was prepared by dissolving 1.00 eq [Ph_3C][B(C_6F_5)] in toluene followed by the addition of 1.00 eq 1M $NaBHEt_3$ toluene solution and stirring for 12 h. The organic phase was decanted, and the colorless residue was washed extensively with *n*-pentane and dried in vacuo. CV measurements were performed with an EmStat3+ Blue from PalmSens Compact Electrochemical Interfaces and processed with PSTrace 5.7 at 298 K. A glassy carbon electrode, with a working area of 0.07 cm^2, was used as a working electrode, a platinic wire as a counter electrode; and a silver wire served as a quasi reference electrode. As internal standard ferrocene was measured at the very end of each measurement. Recrystallized [N^{t}Bu$_4$][BArF$_{20}$] (0.1 M) was used as electrolyte. Elemental analysis was attempted, but yielded unsatisfying results due to extreme moisture and oxygen sensitivity of the compounds.

Synthesis of [Li(dme)][octa-ethylcalix[4]pyrrolato-chloridosilicate] - [Li(dme)][1]

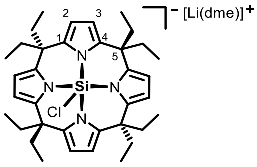

To a solution of 1.00 g *meso*-octaethyl-calix[4]pyrrole (1.84 mmol, 1.00 eq.) in 20 ml *n*-hexane, 4.68 ml 1.6 M *n*-BuLi (7.36 mmol, 4.00 eq.) in hexane was added dropwise at -78 °C and stirred for 20 min. After the solution reached room temperature (rt) it was refluxed for 12 h at 80 °C. Exhaustive deprotonation was verified by ^{1}H NMR spectroscopy in toluene-d$_8$. *n*-Hexane was removed under reduced pressure, and the deprotonated ligand was dissolved in 15 ml dimethoxyethane (DME). 785 mg $SiCl_4$ (4.62 mmol, 2.50 eq.) in 2 ml DME was added dropwise at rt, and the red solution was stirred for 10 min. A conversion to [Li(dme)][**1**] of 40 % was determined by ^{1}H NMR spectroscopy in acetonitrile-d$_3$, beside free *meso*-octaethyl-calix[4]pyrrole and undefined side products. The solvent was removed, and the solid was washed with 20 ml *n*-pentane. Due to the instability of [Li(dme)][**1**] in common solvents except for DME and acetonitrile (ACN), the solid was not further purified but converted directly into the PPh_4 salt. **^{1}H NMR** (ACN-d$_3$, 600 MHz, 295 K) δ[ppm]: 5.75 (d, $^3J_{HH}$ = 3.3 Hz, 4H, H3), 5.70 (d, $^3J_{HH}$ = 3.3 Hz, 4H, H2), 3.46 (s, 4H, $CH_{2\ dme}$), 3.29 (s, 6H,), 2.14 (q, $^3J_{HH}$ = 7.4 Hz, 4H, $CH_{2\ Ethyl}$), 2.01 (q, $^3J_{HH}$ = 7.4 Hz, 4H, $CH_{2\ Ethyl}$), 1.98 (q, 4H, $CH_{2\ Ethyl}$), 1.95 (q, 4H, $CH_{2\ Ethyl}$), 1.13 (t, $^3J_{HH}$ = 7.4 Hz, 6H, $CH_{3\ Ethyl}$), 0.93 (t, $^3J_{HH}$ = 7.4 Hz, 6H, $CH_{3\ Ethyl}$), 0.45 (t, $^3J_{HH}$ = 7.4 Hz, 6H, $CH_{3\ Ethyl}$), 0.26 (t, $^3J_{HH}$ = 7.4 Hz, 6H, $CH_{3\ Ethyl}$). **^{13}C{^{1}H} NMR** (ACN-d$_3$, 150 MHz, 295 K) δ[ppm]: 145.9 (C1/C4), 143.3 (C1/C4), 106.9 (C2), 102.7 (C3), 72.4 ($CH_{2\ dme}$), 58.8 ($CH_{3\ dme}$), 44.7 (C5), 39.1 ($CH_{2\ Ethyl}$), 37.0 (2C, $CH_{2\ Ethyl}$), 26.0 ($CH_{2\ Ethyl}$), 10.8 ($CH_{3\ Ethyl}$), 10.2 ($CH_{3\ Ethyl}$), 9.7 ($CH_{3\ Ethyl}$), 9.3 ($CH_{3\ Ethyl}$). **^{29}Si HMBC NMR** (ACN-d$_3$, 600 MHz, 295 K) δ[ppm]: −129.0.**^{7}Li NMR** (155 MHz, ACN-d$_3$) δ[ppm]: −1.88. **Mass spectrometry** [HR-ESI$^-$]: calc. 599.3336 m/z exp. 599.3377 m/z.

Synthesis of $[PPh_4]$[Ethylcalix[4]pyrrolato-chloridosilicate] - $[PPh_4]$[1]

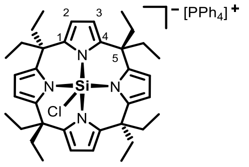

[Li(dme)][**1**] (509 mg, 0.73 mmol, 1.00 eq.) was dissolved in 15 ml acetonitrile and 277 mg PPh_4Cl (0.73 mmol, 1.00 eq.) was added, and the suspension was stirred for 1 h at room temperature. The solvent was removed under reduced pressure, and the solid was dissolved in 5 ml DCM and filtered over a glass frit. The solid was washed until discoloration with small portions of DCM. The solvent was removed from the filtrate, and the residue was washed three times with 50 ml *n*-pentane each. For a better purification, the suspension was put into a sonic bath for 5 min after every washing step. The *n*-pentane was removed by decanting and the solid dried under reduced pressure. The solid was washed three times with 20 ml toluene until no coloration of the toluene washing phase occurred. Last traces of DME were removed by dissolving the solid again in 15 ml DCM and precipitation with 60 ml *n*-pentane. The solvent was decanted, and the residual volatiles were removed in vacuo. Compound $[PPh_4]$[**1**] (0.628 g, 0.67 mmol) was obtained as a colorless solid in 91 % yield. Suitable crystals for SCXRD analysis were obtained from a saturated benzene solution at room temperature. **^{1}H NMR** (DCM-d_2, 400 MHz, 295 K) δ[ppm]: 7.86 (m, 4H, PPh_4^+), 7.67 (m, 8H, PPh_4^+), 7.57 (m, 8H, PPh_4^+), 5.75 (d, $^3J_{HH}$ = 3.2 Hz, 4H, H3), 5.67 (d, $^3J_{HH}$ = 3.2 Hz, 4H, H2), 2.10 (q, $^3J_{HH}$ = 7.4 Hz, 4H, $CH_{2\,Ethyl}$), 2.01 (q, $^3J_{HH}$ = 7.4 Hz, 4H, $CH_{2\,Ethyl}$), 1.99 (q, $^3J_{HH}$ = 7.4 Hz, 4H, $CH_{2\,Ethyl}$), 1.88 (q, $^3J_{HH}$ = 7.4 Hz, 4H, $CH_{2\,Ethyl}$), 1.12 (t, $^3J_{HH}$ = 7.4 Hz, 6H, $CH_{3\,Ethyl}$), 0.91 (t, $^3J_{HH}$ = 7.4 Hz, 6H, $CH_{3\,Ethyl}$), 0.47 (t, $^3J_{HH}$ = 7.4 Hz, 6H, $CH_{3\,Ethyl}$), 0.22 (t, $^3J_{HH}$ = 7.4 Hz, 6H, $CH_{3\,Ethyl}$). **^{13}C{^{1}H} NMR** (DCM-d_2, 100 MHz, 295 K) δ[ppm]: 145.8 (C1/C4), 143.0 (C1/C4), 136.1 (d, $^4J_{CP}$ = 3.1 Hz, CH, PPh_4^+), 134.8 (d, $^2J_{CP}$ = 10.3 Hz, CH, PPh_4^+), 131.0 (d, $^3J_{CP}$ = 12.9 Hz, CH, PPh_4^+), 117.9 (d, $^1J_{CP}$ = 89.8 Hz, C_q, PPh_4^+), 106.0 (C2), 101.9 (C3), 44.3 (C5), 40.6 ($CH_{2\,Ethyl}$), 39.3 ($CH_{2\,Ethyl}$), 36.4 ($CH_{2\,Ethyl}$), 25.4 ($CH_{2\,Ethyl}$), 10.5 ($CH_{3\,Ethyl}$), 10.0 ($CH_{3\,Ethyl}$), 9.3 ($CH_{3\,Ethyl}$), 9.0 ($CH_{3\,Ethyl}$). **^{29}Si HMBC NMR** (DCM-d_2, 400 MHz, 295 K) δ[ppm]: −129.0. **^{31}P NMR** (DCM-d_2, 161 MHz, 295 K) δ[ppm]: 23.22. **Mass spectrometry** [HR-ESI$^-$]: calc. 599.3336 m/z exp. 599.3377 m/z. **IR spectroscopy** ṽ[cm^{-1}]: 3099 (w) , 2958 (m), 2925 (m), 2868 (m), 1507 (w), 1454 (m), 1413 (m), 1374 (m), 1326 (w), 1247 (m), 1076 (s), 1009 (w), 970 (m), 857 (m), 765 (s), 706 (w).

Synthesis of [Ethylcalix[4]pyrrolato-silane] – 2

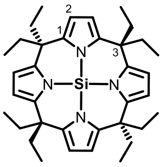

Freshly prepared donor free sodium tetrakis(pentafluorophenyl)borate (68.4 mg, 28.5 µmmol, 1.10 eq.) was added to a solution of $[PPh_4]$[**1**] (66.0 mg, 70.2 µmmol, 1.00 eq.) in 4 ml DCM at room temperature. The solution was stirred for 12 h while turning yellow, and the solvent was removed under reduced pressure. 10 ml *n*-hexane were added, and the solution was stirred for 2 h and filtered off. The solvent of the filtrate was removed, and the residue was dissolved in 10 ml *n*-hexane, stirred for 24 h and filtered. The clear yellow solution was stirred for another 24 h to drive the elimination of NaCl until completeness. The yellow solution was filtered again, and the solvent was removed. After drying in vacuo, the neutral silane **2** was obtained as a yellow solid 78 % yield (30.9 mg, 54.7 µmmol). Orange crystals of **2** were obtained by storing a concentrated solution of **2** in DCM for 7 days at -40 °C. At room temperature, slow decomposition in DCM-d_2 and C_6D_6 was observed over 4 days. Remark: The first step in this reaction is the cation exchange from PPh_4^+ to Na^+. The SCXRD of this intermediate $[Na]_4$[**1**]$_4$ was obtained by storing a concentrated solution of $[PPh_4]$[**1**] and $NaBArF_{20}$ in DCM for 5 days at room temperature without further workup. **^{1}H NMR** (DCM-d_2, 400 MHz, 295 K) δ[ppm]: 5.89 (s, 8H, H2), 2.04 (q, $^3J_{HH}$ = 7.4 Hz, 8H, $CH_{2\,Ethyl}$), 1.93 (q, $^3J_{HH}$ = 7.4 Hz, 8H, $CH_{2\,Ethyl}$), 1.10 (t, $^3J_{HH}$ = 7.4 Hz, 12H, $CH_{3\,Ethyl}$), 0.66 (t, $^3J_{HH}$ = 7.4 Hz, 12H, $CH_{3\,Ethyl}$). **^{13}C{^{1}H} NMR** (DCM-d_2, 100 MHz, 295 K) δ[ppm]: 143.9 (C1), 106.5 (C2), 45.9 ($CH_{2\,Ethyl}$), 42.4 (C3), 24.8 ($CH_{2\,Ethyl}$), 9.4 ($CH_{3\,Ethyl}$), 9.3 ($CH_{3\,Ethyl}$). **^{29}Si HMBC NMR** (DCM-d_2, 400 MHz, 295 K): −55.6 ppm. **Mass spectrometry**: [ESI$^-$]: $C_{36}H_{48}N_4Si{*}OH^-$: calc. 581.3675 m/z exp. 581.3761 m/z. [MALDI, DFTB-Matrix] [m/z]: $C_{36}H_{48}N_4Si{*}H_3O^+$: calc. 583.383 m/z exp. 583.397 m/z. **IR**

spectroscopy ṽ[cm^{-1}]: 3112 (w), 2963 (s), 2931 (s), 2872 (s), 2815 (w), 1344 (w), 1514 (m), 1455 (s), 1378 (m), 1364 (m), 1322 (w), 1297 (w), 1278 (w), 1234 (s), 1132 (s), 1087 (s), 973 (s), 953 (m), 926 (w), 857 (w), 736 (s), 713 (m), 681 (w).

Synthesis of [Ethylcalix[4]pyrrolato-silane-phenylacetylene] activation product

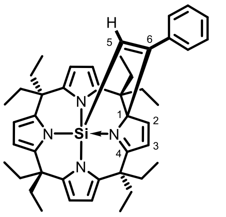

To a solution of **2** (15.0 mg, 26.5 µmol) in 0.5 ml DCM-d_2, phenylacetylene (0.1 mL) was added at room temperature. Quantitative conversion was observed by ^{1}H NMR spectroscopy. Isolation of the activation product was performed as follows: To a solution of [PPh_4][**1**] (70.0 mg, 74.5 µmol, 1.00 eq.) in 5 ml dichloromethane Na[$BArF_{20}$] (57.5 mg, 81.9 µmol, 1.10 eq.) and phenylacetylene (10.0 mg, 96.8 µmol, 1.30 eq.) were added and stirred for 16 h. All volatiles were removed *in vacuo* and the solid was stirred for 30 min in 5 ml *n*-hexane. The yellow solution was filtered and the solvent was removed under reduced pressure. This step was performed twice. The product was dried for 30 min *in vacuo*. The yellow phenylacetylene activation product (35.4 mg, 52.5 µmol) was obtained in 70 % yield. **^{1}H NMR** (DCM-d_2, 600 MHz, 295 K) δ[ppm]: 8.04 (d, $^{3}J_{HH}$ = 5.4 Hz, 1H, H2), 7.26 (m, 3H, H_{phenyl}), 7.19 (m, 2H, $^{3}J_{HH}$ = 5.4 Hz, 1H, H3), 6.07 (s, 1H, H_{alkyne}), 6.05 (d, $^{3}J_{HH}$ = 3.2 Hz, 1H, $H_{pyrrole}$), 6.00 (d, $^{3}J_{HH}$ = 3.2 Hz, 1H, $H_{pyrrole}$), 5.96 (m, 2H, $H_{pyrrole}$), 5.86 (d, $^{3}J_{HH}$ = 3.4 Hz, 1H, $H_{pyrrole}$), 5.78 (d, $^{3}J_{HH}$ = 3.4 Hz, 1H, $H_{pyrrole}$), 2.25 (m, 2H, H_{CH2}), 2.18 (m, 2H, H_{CH2}), 2.07 (m, 2H, H_{CH2}), 1.92 (m, 4H, H_{CH2}), 1.81 (m, 2H, H_{CH2}), 1.73 (m, 2H, H_{CH2}), 1.28 (m, 2H, H_{CH2}), 1.18 (t, $^{3}J_{HH}$ = 7.3 Hz, 3H, H_{CH3}), 1.15 (t, $^{3}J_{HH}$ = 7.3 Hz, 3H, H_{CH3}), 0.78 (t, $^{3}J_{HH}$ = 7.3 Hz, 3H, H_{CH3}), 0.73 (t, $^{3}J_{HH}$ = 7.3 Hz, 3H, H_{CH3}), 0.65 (t, $^{3}J_{HH}$ = 7.3 Hz, 3H, H_{CH3}), 0.61 (t, $^{3}J_{HH}$ = 7.3 Hz, 3H, H_{CH3}), 0.39 (t, $^{3}J_{HH}$ = 7.3 Hz, 3H, H_{CH3}), 0.35 (t, $^{3}J_{HH}$ = 7.3 Hz, 3H, H_{CH3}). **^{13}C{^{1}H} NMR** (DCM-d_2, 150 MHz, 295 K) δ[ppm]: 184.1 (C4), 158.4 (C2), 152.9 (C_q), 149.6 (C_q), 145.2 (C_q), 143.5 (C5), 143.0 (C_q), 142.8 (C_q), 141.1 (C6), 140.1 (C_q), 136.4 (C_q), 128.8 (2C, CH_{phenyl}), 128.5 (2C, CH_{phenyl}), 127.9 (CH_{phenyl}), 127.0 (C3), 111.1 (CH), 109.5 (CH), 108.7 (CH), 105.6 (CH), 105.2 (CH), 105.0 (CH), 95.1 (C1), 55.2 (C_q), 46.0 (C_q), 44.8 (C_q), 42.8 (C_q), 40.7 (CH_2), 37.1 (CH_2), 34.6 (CH_2), 31.3 (CH_2), 29.3 (CH_2), 26.2 (CH_2), 25.8 (CH_2), 23.3 (CH_2), 9.9 (CH_3), 9.8 (CH_3), 9.7 (CH_3), 9.6 (CH_3), 9.4 (CH_3), 9.3 (CH_3), 9.0 (CH_3), 8.0 (CH_3). **^{29}Si HMBC NMR** (DCM-d_2, 600 MHz, 295 K) δ[ppm]: −126.0 ppm. **Mass spectrometry**: [ESI^+]: $C_{44}H_{54}N_4Si*H^+$ calc. 667.4195 m/z exp. 667.4141 m/z.

Reduction of 2 with CoCp*$_2$ in dichloromethane

To a solution of **2** (10.0 mg, 17.7 µmol, 1.00 eq) in DCM-d_2 at room temperature, $CoCp_2^*$ was added stepwise (0.1, 0.2, 0.6 and 1.0 eqivalents), and the solution turned from yellow to light yellow, indicating the formation of chlorido silicate [$CoCp_2$*][**1**] as the major product (Figure S 15). Addition of an excess of $CoCp_2^*$ in one portion lead to the clean formation of [**1**]$^-$ without side products (Figure S 16). The identity of the chlorido silicate [**1**]$^-$ was verified by ^{1}H and ^{29}Si HMBC NMR spectroscopy, ESI-MS and by comparison with the other salts of [**1**]$^-$. Due to the rapid reaction, no radical intermediate could be detected by EPR-spectroscopy. The fate of the chloride atom donor was not inspected.

Supplemental References

1. Otwinowski, Z., and Minor, W. (1997). Processing of X-ray diffraction data collected in oscillation mode. Methods Enzymol. *276*, 307-326.
2. SAINT (2016) (Bruker AXS GmbH, Karlsruhe, Germany).
3. Sheldrick, G.M. (2004-2014) (SADABS, Bruker AXS GmbH, Karlsruhe, Germany).
4. Krause, L., Herbst-Irmer, R., Sheldrick, G.M., and Stalke, D. (2015). Comparison of silver and molybdenum microfocus X-ray sources for single-crystal structure determination. J. Appl. Crystallogr. *48*, 3-10.
5. Sheldrick, G.M. (2014-2018) (SHELXT, Program for Crystal Structure Solution, University of Göttingen, Germany).
6. Sheldrick, G.M. (2015). SHELXT - Integrated space-group and crystal-structure determination. Acta Crystallogr. A, 3-8.
7. Sheldrick, G.M. (2012-2018) (SHELXL-20xx, University of Göttingen and Bruker AXS GmbH, Karlsruhe, Germany).
8. Robinson, W., and Sheldrick, G.M. (1988). Crystallographic Computing 4, N.W. Isaaks, and M.R. Taylor, eds. (Ch. 22, IUCr and Oxford University Press, Oxford, UK).
9. Sheldrick, G.M. (2008). A short history ofSHELX. Acta Crystallogr. A *64*, 112-122.
10. Sheldrick, G.M. (2015). Crystal structure refinement withSHELXL. Acta Crystallogr. C *71*, 3-8.
11. Dolomanov, O.V., Bourhis, L.J., Gildea, R.J., Howard, J.A.K., and Puschmann, H. (2009). OLEX2: a complete structure solution, refinement and analysis program. J. Appl. Crystallogr. *42*, 339-341.
12. Capelli, S.C., Burgi, H.-B., Dittrich, B., Grabowsky, S., and Jayatilaka, D. (2014). Hirshfeld atom refinement. IUCrJ *1*, 361-379.
13. Woińska, M., Grabowsky, S., Dominiak, P.M., Woźniak, K., and Jayatilaka, D. (2016). Hydrogen atoms can be located accurately and precisely by x-ray crystallography. Sci. Adv. *2*, e1600192.
14. Kleemiss, F., Puschmann, H., Dolomanov, O., Bodensteiner, M., Peyerimhoff, N., Midgley, L., Bourhis, L., Genoni, A., Malaspina, L.A., Jayatilaka, D.*, et al.* (2020). Generalizing Non-Spherical Structure Refinement: Hirshfeld Atom Refinement in NoSpherA2. Chem. Sci., DOI: 10.1039/D1030SC05526C.
15. Neese, F. (2012). The ORCA program system. WIREs Comput. Mol. Sci. *2*, 73-78.
16. Andrienko, G.A. (https://www.chemcraftprog.com).
17. Grimme, S., Brandenburg, J.G., Bannwarth, C., and Hansen, A. (2015). Consistent structures and interactions by density functional theory with small atomic orbital basis sets. J. Chem. Phys. *143*, 054107.
18. Kruse, H., and Grimme, S. (2012). A geometrical correction for the inter- and intra-molecular basis set superposition error in Hartree-Fock and density functional theory calculations for large systems. J. Chem. Phys. *136*, 154101.
19. Grimme, S., Antony, J., Ehrlich, S., and Krieg, H. (2010). A consistent and accurate ab initio parametrization of density functional dispersion correction (DFT-D) for the 94 elements H-Pu. J. Chem. Phys. *132*, 154104.
20. Grimme, S., Ehrlich, S., and Goerigk, L. (2011). Effect of the damping function in dispersion corrected density functional theory. J. Comput. Chem. *32*, 1456-1465.
21. Sure, R., Brandenburg, J.G., and Grimme, S. (2016). Small Atomic Orbital Basis Set First-Principles Quantum Chemical Methods for Large Molecular and Periodic Systems: A Critical Analysis of Error Sources. ChemistryOpen *5*, 94-109.
22. Frenzel, A., Herbst-Irmer, R., Klingebiel, U., Noltemeyer, M., and Schäfer, M. (1995). Indolyl- und Pyrrolylsilane – Synthese und Kristallstruktur / Indolyl- and Pyrrolylsilanes – Syntheses and Crystal Structures. Z. Naturforsch. B *50*, 1658.
23. Zhao, Y., and Truhlar, D.G. (2005). Design of Density Functionals That Are Broadly Accurate for Thermochemistry, Thermochemical Kinetics, and Nonbonded Interactions. J. Phys. Chem. A *109*, 5656-5667.
24. Weigend, F., and Ahlrichs, R. (2005). Balanced basis sets of split valence, triple zeta valence and quadruple zeta valence quality for H to Rn: Design and assessment of accuracy. Phys. Chem. Chem. Phys. *7*, 3297-3305.
25. Stoychev, G.L., Auer, A.A., and Neese, F. (2017). Automatic Generation of Auxiliary Basis Sets. J. Chem. Theory Comput. *13*, 554-562.
26. Neese, F., Wennmohs, F., Hansen, A., and Becker, U. (2009). Efficient, approximate and parallel Hartree–Fock and hybrid DFT calculations. A 'chain-of-spheres' algorithm for the Hartree–Fock exchange. Chem. Phys. *356*, 98-109.

27. Neese, F. (2003). An improvement of the resolution of the identity approximation for the formation of the Coulomb matrix. J. Comput. Chem. *24*, 1740-1747.
28. Reed, A.E., Curtiss, L.A., and Weinhold, F. (1988). Intermolecular interactions from a natural bond orbital, donor-acceptor viewpoint. Chem. Rev. *88*, 899-926.
29. Glendening, E.D., Landis, C.R., and Weinhold, F. (2012). Natural bond orbital methods. Wiley Interdiscip. Rev. Comput. Mol. Sci *2*, 1-42.
30. Bader, R.F.W. (1991). A quantum theory of molecular structure and its applications. Chem. Rev. *91*, 893-928.
31. Adamo, C., and Barone, V. (1999). Toward reliable density functional methods without adjustable parameters: The PBE0 model. J. Chem. Phys. *110*, 6158-6170.
32. Ernzerhof, M., and Scuseria, G.E. (1999). Assessment of the Perdew–Burke–Ernzerhof exchange-correlation functional. J. Chem. Phys. *110*, 5029-5036.
33. Medvedev, M.G., Bushmarinov, I.S., Sun, J., Perdew, J.P., and Lyssenko, K.A. (2017). Density functional theory is straying from the path toward the exact functional. Science *355*, 49-52.
34. NBO 7.0. E. D. Glendening, J. K. Badenhoop, A. E. Reed, J. E. Carpenter, J. A. Bohmann, C. M. Morales, P. Karafiloglou, C. R. Landis, and F. Weinhold, Theoretical Chemistry Institute, University of Wisconsin, Madison, WI (2018)
35. AIMAll (Version 19.10.12), Todd A. Keith, TK Gristmill Software, Overland Park KS, USA, 2019 (aim.tkgristmill.com)
36. Thakur, T.S., and Desiraju, G.R. (2007). Theoretical investigation of C–H···M interactions in organometallic complexes: A natural bond orbital (NBO) study. J. Mol. Struc.-Theochem *810,* 143-154.
37. Scherer, W., and McGrady, G.S. (2004). Agostic Interactions in d0 Metal Alkyl Complexes. Angew. Chem. Int. Edit. *43,* 1782-1806.
38. Lu, Q., Neese, F., and Bistoni, G. (2018). Formation of Agostic Structures Driven by London Dispersion. Angew. Chem. Int. Edit. *57,* 4760-4764.
39. Lin, X., Wu, W., and Mo, Y. (2020). A theoretical perspective of the agostic effect in early transition metal compounds. Coordin. Chem. Rev. *419,* 213401.
40. Lein, M. (2009). Characterization of agostic interactions in theory and computation. Coordin. Chem. Rev. *253,* 625-634.
41. Tognetti, V., Joubert, L., Raucoules, R., De Bruin, T., and Adamo, C. (2012). Characterizing Agosticity Using the Quantum Theory of Atoms in Molecules: Bond Critical Points and Their Local Properties. J. Phys. Chem. A *116,* 5472-5479.
42. Neese, F., Hansen, A., and Liakos, D.G. (2009). Efficient and accurate approximations to the local coupled cluster singles doubles method using a truncated pair natural orbital basis. J Chem Phys *131.*
43. Riplinger, C., and Neese, F. (2013). An efficient and near linear scaling pair natural orbital based local coupled cluster method. J Chem Phys *138.*
44. Riplinger, C., Sandhoefer, B., Hansen, A., and Neese, F. (2013). Natural triple excitations in local coupled cluster calculations with pair natural orbitals. J Chem Phys *139.*
45. Weigend, F., Furche, F., and Ahlrichs, R. (2003). Gaussian basis sets of quadruple zeta valence quality for atoms H–Kr. J. Chem. Phys. *119,* 12753-12762.
46. Lee, T.J., and Taylor, P.R. (1989). A diagnostic for determining the quality of single-reference electron correlation methods. Int. J. Quantum Chem. *36,* 199-207.
47. Yanai, T., Tew, D.P., and Handy, N.C. (2004). A new hybrid exchange–correlation functional using the Coulomb-attenuating method (CAM-B3LYP). Chem. Phys. Lett. *393,* 51-57.
48. Martin, R.L. (2003). Natural transition orbitals. J. Chem. Phys. *118,* 4775-4777.
49. Jacoby, D., Floriani, C., Chiesi-Villa, A., and Rizzoli, C. (1991). The π and σ bonding modes of meso-octaethylporphyrinogen to transition metals: the X-ray structure of a meso-octaethylporphyrinogen–zirconiuml(IV) complex and of the parent meso-octaethylporphyrinogen ligand. J. Chem. Soc. Chem. Comm., 790-792.
50. Depraetere, S., Smet, M., and Dehaen, W. (1999). N-confused calix[4]pyrroles. Angew Chem Int Edit *38,* 3359-3361.
51. De Angelis, S., Solari, E., Floriani, C., Chiesi-Villa, A., and Rizzoli, C. (1994). Dalton communications. Solvent-dependent forms of lithiated 5,5,10,10,15,15,20,20-octaethylporphyrinogen in solution and in the solid state and reaction with tetrahydrofuran. J. Chem. Soc. Dalton, 2467-2469.
